# The genome of the venomous snail *Lautoconus ventricosus* sheds light on the origin of conotoxin diversity

**DOI:** 10.1093/gigascience/giab037

**Published:** 2021-05-25

**Authors:** José Ramón Pardos-Blas, Iker Irisarri, Samuel Abalde, Carlos M L Afonso, Manuel J Tenorio, Rafael Zardoya

**Affiliations:** Departamento de Biodiversidad y Biología Evolutiva, Museo Nacional de Ciencias Naturales (MNCN-CSIC), José Gutiérrez Abascal 2, 28006 Madrid, Spain; Departamento de Biodiversidad y Biología Evolutiva, Museo Nacional de Ciencias Naturales (MNCN-CSIC), José Gutiérrez Abascal 2, 28006 Madrid, Spain; Department of Applied Bioinformatics, Institute for Microbiology and Genetics, University of Goettingen, Goldschmidtstr. 1, D-37077 Goettingen, Germany; Campus Institute Data Science (CIDAS), Goettingen, Wilhelmsplatz 1, D-37073, Germany; Departamento de Biodiversidad y Biología Evolutiva, Museo Nacional de Ciencias Naturales (MNCN-CSIC), José Gutiérrez Abascal 2, 28006 Madrid, Spain; Department of Zoology, Swedish Museum of Natural History, Frescativägen 40, 11418 Stockholm, Sweden; Centre of Marine Sciences (CCMAR), Universidade do Algarve, Campus de Gambelas, 8005–139 Faro, Portugal; Departamento CMIM y Q. Inorgánica-INBIO, Facultad de Ciencias, Universidad de Cadiz, 11510 Puerto Real, Cádiz, Spain; Departamento de Biodiversidad y Biología Evolutiva, Museo Nacional de Ciencias Naturales (MNCN-CSIC), José Gutiérrez Abascal 2, 28006 Madrid, Spain

**Keywords:** Mediterranean cone snail, Lautoconus ventricosus, chromosome-level genome, venom gland transcriptome, conotoxin precursor genes, whole-genome duplication

## Abstract

**Background:**

Venoms are deadly weapons to subdue prey or deter predators that have evolved independently in many animal lineages. The genomes of venomous animals are essential to understand the evolutionary mechanisms involved in the origin and diversification of venoms.

**Results:**

Here, we report the chromosome-level genome of the venomous Mediterranean cone snail, *Lautoconus ventricosus* (Caenogastropoda: Conidae). The total size of the assembly is 3.59 Gb; it has high contiguity (N50 = 93.53 Mb) and 86.6 Mb of the genome assembled into the 35 largest scaffolds or pseudochromosomes. On the basis of venom gland transcriptomes, we annotated 262 complete genes encoding conotoxin precursors, hormones, and other venom-related proteins. These genes were scattered in the different pseudochromosomes and located within repetitive regions. The genes encoding conotoxin precursors were normally structured into 3 exons, which did not necessarily coincide with the 3 structural domains of the corresponding proteins. Additionally, we found evidence in the *L. ventricosus* genome for a past whole-genome duplication event by means of conserved gene synteny with the *Pomacea canaliculata* genome, the only one available at the chromosome level within Caenogastropoda. The whole-genome duplication event was further confirmed by the presence of a duplicated *hox* gene cluster. Key genes for gastropod biology including those encoding proteins related to development, shell formation, and sex were located in the genome.

**Conclusions:**

The new high-quality *L. ventricosus* genome should become a reference for assembling and analyzing new gastropod genomes and will contribute to future evolutionary genomic studies among venomous animals.

## Background

The use of venoms is one of the most sophisticated ways found in nature to efficiently subdue prey or deter predators [1, 2]. Even though the production of venom is energetically expensive, these deadly bioactive compounds confer a selective advantage, and thus their use has evolved recurrently in many distinct animal lineages such as jellyfish, centipedes, wasps, scorpions, spiders, cone snails, stonefish, and snakes [3, 4]. Snakes are undoubtedly the most dangerous to humans and are widely accepted as the main model system in venom research, having been the subject of pioneering research applying methodological advances [5] and formulating the postulation of hypotheses in the field [6–8].

Each venomous animal lineage represents an independent evolutionary experiment in which selective pressures have arrived at unique combinations of versatile venoms, whose compositions are dynamically adjusted at the genetic, transcriptional, and protein levels [4]. The comparison of these venomous animal lineages at the different levels within a phylogenetic framework should provide evolutionary insights on how the diversity of venoms is originated and maintained, as well as contribute to therapeutic advances [2]. In this regard, the powerful combination of high-throughput proteomics and transcriptomics is allowing the systematic cataloguing of the venom arsenals of numerous animal species beyond snakes (e.g., [9, 10]), including some previously neglected taxa [11]. These valuable data need to be complemented by genomic data to ensure gene completeness and homology prediction [12]. Moreover, identifying the ongoing evolutionary processes governing the genetic control of venom variation ultimately requires the sequencing of the genomes of various venomous animals to find common patterns and gain knowledge on how toxin-encoding genes are distributed within the different genomes and their exact copy number, exon/intron structure, conserved synteny to other genes, regulatory regions, or potential association to repetitive elements. However, the advance of comparative genomics of venomous animals still awaits the necessary impetus. Although several genomes of venomous animals are available, most were generated with short-read technology, which resulted in fragmented assemblies not amenable to answering most of the aforementioned questions [13–15]. One notable exception is the comparative analysis of the Hispaniolan solenodon genome that demonstrated the convergent origin of venoms in eulipotyphlan mammals [16]. Recently, the chromosome-level genome assembly of the Indian cobra *Naja naja* was reported [17]. The contiguity of this genome allowed the determination of the organization and localization of a set of 139 toxin-encoding genes classified into 33 gene families [17]. Genomes of 2 jellyfish have also been recently assembled at the chromosomal level [18] although not used to study venom evolution.

With >900 species, cone snails are a highly diverse natural group living preferentially in the intertidal zone of tropical and subtropical regions worldwide [19]. They are key marine predators that produce venom to prey on worms, snails, and fish, as well as to defend against predators [20]. The venom is a cocktail composed of hundreds of peptides named conotoxins, which are synthesized as precursors with a 3-domain structure: a conserved signal region (used to classify precursors into “superfamilies” [21]); a pro-peptide region involved in the processing of the precursor [22]; and a highly variable, cysteine-rich mature region, which is the functional toxin [23]. It has been proposed that the striking hyperdiversity of conotoxins has been generated through the combination of different mechanisms, including gene duplication, accelerated substitution rates, recombination, alternative splicing, differential expression, and post-translational modifications [24–28].

Here, we report *de novo* chromosome-level genome and transcriptome assemblies of the Mediterranean cone snail *Lautoconus ventricosus* (Gmelin, 1791), a vermivorous species that inhabits the Mediterranean Sea and nearby Atlantic coast. Previous attempts to sequence and assemble the genome of a cone snail using short-read technology were largely unsuccessful [14, 29, 30]. The high contiguity of the newly assembled genome (together with the comprehensive catalogue of transcripts encoding conotoxin precursors derived from the venom gland transcriptome) allowed us to determine the organization of the conotoxin genes in the genome and to shed light on the genomic basis of conotoxin diversity. Moreover, because few chromosome-level genomes are available for gastropods, the cone snail genome will be particularly useful for wider evolutionary genomic studies in mollusks. In this regard, we compared the *L. ventricosus* genome to that of the ampullariid *Pomacea canaliculata* [31], the only other caenogastropod genome assembled at the chromosomal level. This comparison revealed in the *L. ventricosus* genome, the presence of a past whole-genome duplication (WGD), which was previously hypothesized using chromosomal counts to have occurred in the ancestor of Neogastropoda and related families [32].

## Results and Discussion

### *De novo* sequencing, assembly, and annotation of the *L. ventricosus* genome

A high-quality assembly of the Mediterranean cone snail *L. ventricosus* was generated from PacBio, Chicago, and Dovetail Hi-C libraries. First, 192.6 Gb of long-read sequence data (54× coverage) were produced with PacBio Sequel II and assembled *de novo* into 46,042 contigs (N50 = 185.88 kb; the largest contig was 1.71 Mb). Little signature of potential exogenous DNA contamination was detected (Supplementary Fig. S1). In parallel, a total of 761 and 680 Gb of short-read sequence data were produced with Illumina HiSeq X from the Chicago and Hi-C libraries, respectively. Together, the Chicago library reads provided 5.25× physical coverage of the genome (1–100 kb pairs) and the Hi-C library reads provided 381.12× physical coverage of the genome (10–10,000 kb pairs).

**Figure 1:**
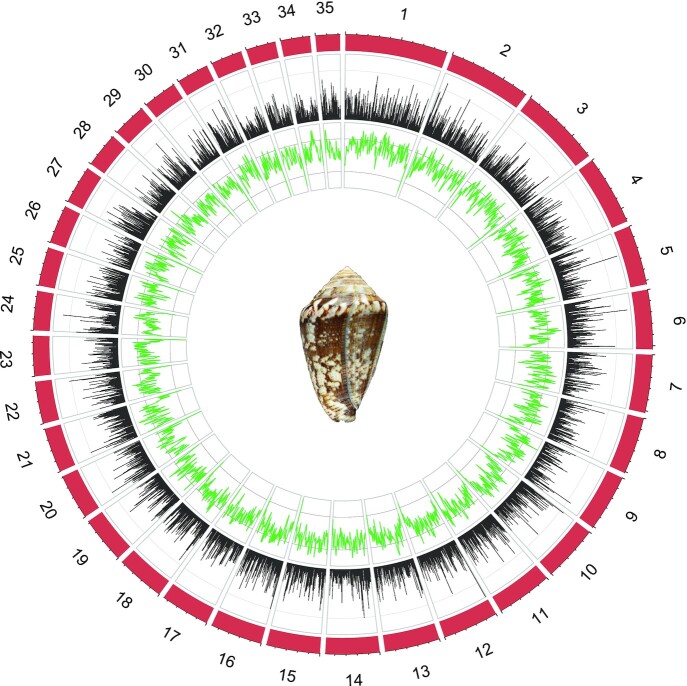
Genome organization. The 35 pseudochromosomes of the *L. ventricosus* genome are shown in red. In the inner rings, the distributions of genes (black; y-axis indicates percentage of genes per megabase, normalized to 40 genes) and of repetitive elements (green; y-axis indicates percentage of repetitive elements per megabase, normalized to 6,000 repetitive elements) are depicted.

A second assembly round using proximity ligation information led to 19,399 scaffolds, the largest having 184.22 Mb (Supplementary Table S1). The N50 was 93.52 Mb and 86.6% of the genome was assembled into the 35 largest scaffolds or pseudochromosomes (Fig. 1 and Supplementary Fig. S2). The total size of the assembly was 3.59 Gb, which represents 87.6% of the haploid genome size estimated by means of flow cytometry (4.1 Gb). Together with the cephalopod *Euprymna scolopes* (5.1 Gb [33]), they are the largest mollusk genomes thus far sequenced [34]. Within gastropods, it is twice the size of that of *Achatina immaculata* (1.75 Gb [35]) and ∼8 times larger than most gastropod genomes including those of *P. canaliculata* (446 Mb [31]), *Chrysomallon squamiferum* (444 Mb [36]), and *Lottia gigantea* (348 MB [37]). The obtained genome size is larger than the estimated 3.02 Gb for *Pionoconus consors* [29], 2.76 Gb for *Kioconus tribblei* [14], and 2.56 Gb for *Textilia bullata* [30] using *k*-mer frequency distribution and simulations. However, it matches the 3.60 Gb of the *Darioconus pennaceus* genome and is smaller than the 3.90 Gb of the *Lividoconus lividus* genome, which were estimated on the basis of fluorometric assays of sperm cells [38]. With regards to the haploid number of chromosomes in Conidae, it generally varies from n = 16 in *Pionoconus magus* [39] to n = 35 in *Virroconus coronatus* [40]. This range in chromosome numbers is common within gastropods [41]. For *L. ventricosus* (as its synonym *Conus mediterraneus*), the haploid number of chromosomes was estimated to be n = 36, although a few specimens had 34, 35, or 37 chromosomes [42]. Therefore, either our specimen had 35 chromosomes and the chromosome numbers vary among the Mediterranean populations or the scaffolding failed to reconstruct 1 chromosome.

The 35 assembled pseudochromosomes varied in size from 44 to 184 Mb (Supplementary Fig. S3). The overall G+C content of the genome was 43.78%, above the 29.74% inferred from the partial genome of *K. tribblei* [14] and the 33–40% generally reported for gastropods [31]. We could not estimate the heterozygosity of the genome assembly on the basis of the PacBio CLR sequence data owing to the error rate associated with the long reads [43]. Instead, we estimated 1.05–1.08% heterozygosity from transcriptome reads, which were obtained with the Illumina technology. This heterozygosity (restricted to coding regions) is similar to that estimated for the gastropod *Haliotis rufescens* and within the range estimated for different mollusks [44]. The repeat regions were homogenously distributed in the genome (Fig. 1) and occupied 53.36% of the genome (Class I transposable elements [TEs], 17.69%; Class II TEs, 11.42%; simple repeats, 10.29%), which is a high proportion compared with *P. canaliculata* (11.4% [31]) or *C. squamiferum* (25.2% [36]), but this variation could be in part due to differences in assembly and repeat annotation. A total of 32,675 protein-coding genes were predicted, adding up to 35.9 Mb (1% of the genome). This large number of protein-coding genes exceeds the average gene content reported for gastropods [31, 34–37] and is comparable to the gene content of cephalopods [45] and sponges [46]. Strikingly, the genome of the scallop *Pecten maximus* has been estimated to contain >67,000 protein-coding genes owing to extensive gene duplication events followed by little gene loss [47]. The genome assembly contained 792 single-copy (82%) and 28 duplicated (2.9%) complete genes, as well as 41 fragmented genes (4.3%) of the BUSCO Metazoan ortholog database (odb) 10 [48]. The completeness is similar to that reported for *Achatina fulica* (91.7% [34]) and lower than those of *C. squamiferum* (96.6% [36]) and *P. canaliculata* (98.9% [31]). The BUSCO metrics for the annotated gene models were much lower: complete single-copy, 31.2%; complete duplicated, 0.4%; fragmented, 21.7%. The main methodological limitation that may explain the missing loci would be the error rate associated with the PacBio CLR sequencing technology (15% [49]), which, despite being partially corrected by coverage, would hamper BLAST similarity searches.

### Genome distribution and structure of conotoxin precursor genes

The transcriptome of the venom gland of another *L. ventricosus* specimen was used to identify and annotate venom-related transcripts, i.e., those encoding conotoxin precursors, hormones, and proteins involved in the processing of conotoxins or in enhancing venom activity. A total of 289 different transcripts were identified using BLAST searches. Of these, 245 transcripts were assigned to 54 conotoxin precursor superfamilies on the basis of the divergence of the signal domain and the presence of different cysteine frameworks; 11 transcripts were classified into 9 hormone gene families; and 33 were assigned to 11 gene families encoding proteins related to venom synthesis or function (Supplementary Table S2 and File S1). These numbers are in agreement with those typically reported for other venom gland transcriptomes of cones [9, 29, 50–53]. Most (94%) transcripts were assembled with a complete open reading frame. As in other cone venom gland transcriptomes [9, 50, 52], O1, T, M, O2, and Conkunitzin superfamilies were the most diverse (Supplementary Table S2 and File S1).

The foot transcriptomes of another 2 specimens were generated for genome annotation (Supplementary Table S2 and File S1). Surprisingly, foot transcriptomes also contained transcripts encoding for conotoxin precursors. There have been reports of minor conotoxin expression outside the venom gland in the salivary gland of *Puncticulis pulicarius* [54] and in the radular sac of *Gastridium geographus* [20], but this is the first report of several conotoxin precursor transcripts in the foot. The 2 foot transcriptomes contained, respectively, a total of 35 and 49 conotoxin precursor, 3 and 1 hormone, and 25 and 19 other venom-related protein transcripts (Supplementary Table S2 and File S1). A total of 15–20% of the conotoxin precursor superfamilies detected in the venom gland were also co-expressed in the foot, as were 9–27% of the hormone families and 58–73% of the other venom-related protein families. Most conotoxin precursor superfamilies expressed in the foot had expression values that were lower by ∼1 order of magnitude than in the venom gland (Supplementary Table S3 and Fig. S4). For B2, I1, M, and Cver01 superfamilies, expression was up to 2 orders of magnitude lower whereas A and Q superfamilies showed similar expression levels in both tissues (Supplementary Table S3 and Fig. S4). The transcripts encoding insulin-related peptides 1, 3, and 4 were exclusively expressed in the venom gland, and the latter showed the highest expression levels. The transcripts encoding insulin-related peptide 2, Prohormone-4b, and the other venom-related proteins had 1 order of magnitude higher expression levels in the foot than in the venom gland (2 orders of magnitude for conoporin; Supplementary Table S3 and Fig. S4), indicating that these hormones and proteins may be endogenous, having a physiological function common to different tissues and not restricted to the venom gland. This could be the case of ferritin, which shows high expression in the distal section of the venom gland of *Chelyconus ermineus* [50] and was highly expressed in the foot of *L. ventricosus*. This is a protein that generally regulates the storage and release of iron and has been related to the incorporation of iron into the radula in some chitons [44] and limpets [55] and into the shell in the pearl oyster [56]. Altogether these results corroborate the specialized secretory function of the venom gland, which is expressing higher levels of conotoxin precursor and some insulin-related transcripts. At the same time, they also point to the presence of a basal (“leaky”) expression of those transcripts in the foot, which is not deleterious for the animal. As in other gastropods, the foot of the cone snail produces mucus, and low levels of conotoxins in the mucus could have an antimicrobial role, as has been demonstrated for conotoxins from *Ximeniconus ximenes* [57] and *Californiconus californicus* [58]. The detection of low expression levels of toxin genes in different tissues outside the venom gland has been demonstrated in snakes [59] and the platypus [60]. To explain the evolutionary origin of this pattern, it has been suggested that toxin genes could emerge either through gene duplication and adaptive neofunctionalization of physiological genes in the venom gland coupled with reduction of expression levels in other tissues [7, 59] or alternatively by subfunctionalization through neutral evolution and restriction to the venom gland [8].

**Figure 2:**
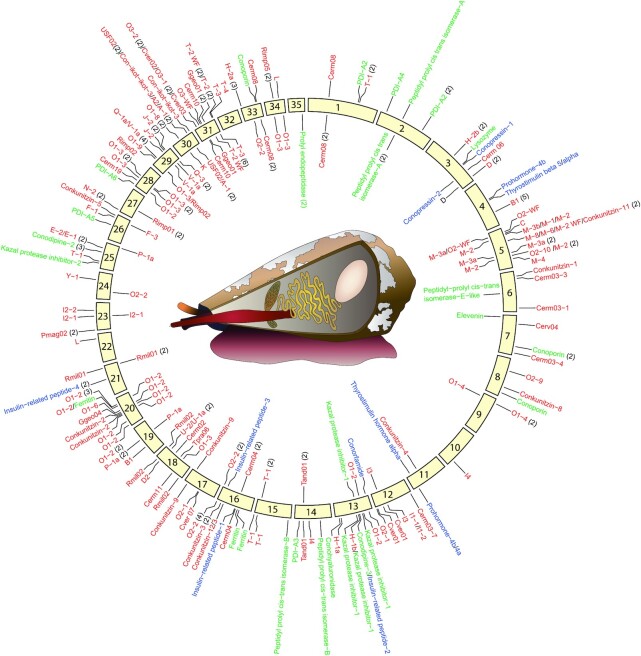
Conotoxin genes. The distribution of the conotoxin precursor (red), hormone (blue), and venom-related protein (green) genes in the 35 pseudochromosomes is shown. Genes closer than 2 Mb were clustered together and their number annotated in parentheses. A cone snail sketch (drawn by Lara de la Cita) highlighting (from left to right) the siphon (orange), proboscis (red), and radular sac (brown) and the duct (yellow) and bulb (white) of the venom gland is shown.

Venom-related transcripts were used to locate the corresponding genes in the pseudochromosomes of the genome (Fig. 2). First, BLASTN searches (1e^−5^) using the 289 transcripts of the *L. ventricosus* transcriptome as query were performed against the 35 pseudochromosomes (Supplementary Table S4, Fig. S5, and File S2). A total of 233 genes were found complete in the genome. Of these, 154 genes were located complete in the 35 pseudochromosomes and the remaining 79 genes were completed manually with hits located in smaller scaffolds, contigs, and raw reads (Supplementary Table S4, Fig. S5, and File S2). Of the 233 complete genes, 213 corresponded to transcripts of the *L. ventricosus* venom gland transcriptome (74%) and the remaining 20 genes were not expressed. The percentage of the transcriptome detected in the genome assembly is considerably lower than that expected according to general BUSCO results (89.2%). The extra 15% of transcripts of the transcriptome without a gene counterpart in the genome could be isoforms that could be produced naturally during expression or generated as artifacts during transcriptome assembly. To test whether other assemblers could improve the final transcriptome, we ran TransPi [61], a program that uses various assemblers and *k*-mers to generate a non-redundant consensus *de novo* transcriptome. However, the resulting *L. ventricosus* venom gland transcriptome did not significantly improve the BUSCO scores obtained using Trinity alone (not shown). It should also be noted that part of the discrepancy between number of conotoxin genes versus transcripts may be due to natural variation among individuals, which has been reported in *L. ventricosus*, at least among populations [62].

Furthermore, we searched for the presence of extra (non-expressed) venom-related genes in the genome by performing BLASTN searches using venom-related transcripts derived from the transcriptomes of closely related cone snail species [9] as query against the 35 pseudochromosomes (Supplementary Table S4, Fig. S5, and File S2). A total of 28 genes were found complete in the 35 pseudochromosomes, and 1 more was manually completed with an exon in 1 of the smaller scaffolds. These extra loci (together with the 20 non-expressed genes detected using *L. ventricosus* transcripts as query; see above) indicate that ≥17% of the venom-related genes found complete in the genome were not expressed in the transcriptome. This proportion of non-expressed precursors is lower than the 41% estimated in *K. tribblei* [14] and the 37–76% reported for several cone species based on exon capture data [63].

A total of 134 venom-related loci in the *L. ventricosus* genome represented incomplete genes. Of these, 62 loci corresponded to genes with >1 exon and the remaining 72 were single exons. These incomplete genes could correspond to any of the transcripts of the *L. ventricosus* transcriptome not assigned previously or to non-expressed genes; and it cannot be excluded that some of the single exons could represent false exon redundancies caused by long repeats during the assembly and scaffolding of PacBio CLR long reads [64].

Although venom-related genes were located throughout the genome, their distribution did not correlate with the size of pseudochromosomes (linear regression, *R*^2^ = 0.005; *P* = 0.67; Supplementary Fig. S6). Pseudochromosomes 5, 16, 18, 20, and 28–31 were particularly rich in conotoxin precursor genes; pseudochromosomes 10, 11, and 22 barely had 1 or 2; only pseudochromosomes 2, 32, and 35 lacked any of these genes at all (Fig. 2 and Supplementary Table S4). The genes were generally found in regions harboring similar Class I retrotransposons such as Gypsy, Penelope, or RTE elements, as well as Class II DNA transposons such as Tc1-Mariner (within <100 kb upstream and downstream). Genes encoding hormones were located in pseudochromosomes 3, 4, 11, 13, 16, and 21 (Fig. 2 and Supplementary Table S4). Genes encoding other venom-related proteins were found in pseudochromosomes 1–3, 6–8, 13–16, 20, 25, 26, 28, 33, and 35 (Fig. 2 and Supplementary Table S4). A scattered distribution of venom-related genes is also found in the genome of the Indian cobra, although in this case, some of the genes have experienced several rounds of tandem gene duplication and are organized in arrays within a pseudochromosome [17]. In the cone snail genome, potential arrays of B1 superfamily genes were found in pseudochromosome 4, of conkunitzin genes in pseudochromosome 16, of O1 superfamily genes in pseudochromosomes 20 and 28, and of I2 superfamily genes in pseudochromosome 23 (Fig. 2 and Supplementary Table S4).

The majority (62.6%) of the complete conotoxin precursor genes had 3 exons and 2 introns (Supplementary Table S4). This proportion is slightly lower than the reported 70% of conotoxin precursor genes having 3 exons based on exon-capture data across several cone snail species [63]. The structures of conotoxin precursor genes found in the *L. ventricosus* genome and those inferred from exon capture data show that genes encoding B1 and J superfamily peptides consistently have a single exon whereas other genes such as A and conodipine normally have 2 exons [63] (see also [14, 65]). Other venom-related genes typically have 11 (protein disulfide isomerases), 8 (lysozyme), 7 (conohyaluronidase), and 4 (kazal protease inhibitor, conoporin) exons (Supplementary Table S4). The boundaries of the first and second exons do not necessarily coincide with the boundaries of signal and pro-peptide domains, but the third exon generally encodes exclusively for the mature domain (Supplementary Fig. S7). This pattern is in agreement with results obtained on the basis of exon-capture data for the mature domain [63]. The average length of introns 1 and 2 was 5,000 bp (Supplementary Fig. S8), exceeding the 2,665 bp reported in *K. tribblei* [14].

### Whole-genome duplication

**Figure 3:**
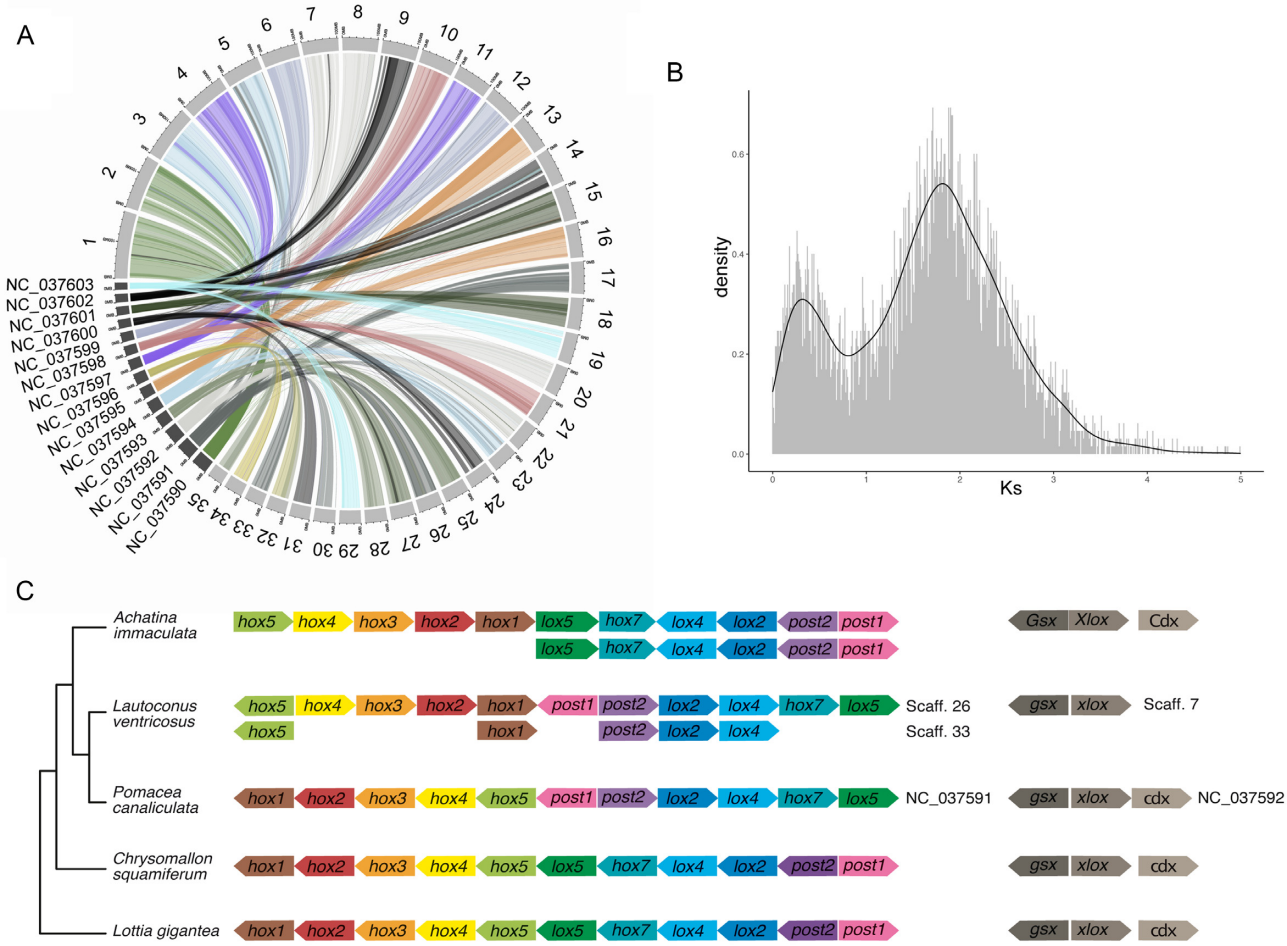
Conserved synteny and whole-genome duplication. (A) Conserved synteny between *L. ventricosus* and *P. canaliculata* derived from ortholog proteins. (B) Distribution of synonymous divergence (*Ks*) between pairs of paralogs in *L. ventricosus*. The second *Ks* peak indicates the similar divergence between paralogs after the whole-genome duplication. (C) Annotation of *hox* and *parahox* clusters in *L. ventricosus* and comparison with other available gastropod genomes within a phylogenetic framework.

Comparisons of homologous gene pairs between *P. canaliculata* [31] and *L. ventricosus* genomes at the chromosome level revealed a clear pattern of conserved macrosynteny in which every chromosome of *P. canaliculata* roughly corresponded to 2–4 chromosomes of *L. ventricosus* (Fig. 3A and Supplementary Fig. S9). This pattern supports the existence of an ancient WGD event during the evolutionary history of Caenogastropoda and explains the increase in chromosome number (14 vs 35) and genome size (446 Mb vs 3.59 Gb). In addition to the WGD, the occurrence of additional chromosomal fissions needs to be postulated. In this regard, several smaller microsyntenic regions throughout the genome were observed (Supplementary Fig. S9), suggesting a dynamic gene reorganization after the WGD. Moreover, the distribution of synonymous substitution rate (*Ks*) values between paralog pairs further supported a WGD event, evidenced by the presence of a second *Ks* peak, which would correspond to the divergence between paralogs from the 2 ancestrally duplicated subgenomes (Fig. 3B [66]).

The existence of a WGD event within Caenogastropoda was already predicted on the basis of chromosome count data [32]. The WGD event was inferred to have occurred within a clade including hypsogastropodan families with an anterior inhalant siphon as morphological synapomorphy [67–69]. Specifically, the WGD event would have occurred after the divergence of families Strombidae and Calyptraeidae, in the ancestor of a lineage containing Ranellidae, Cypraeidae, Capulidae, and the Neogastropoda (which includes Conidae [32]). As new chromosome-level genomes of Hypsogastropoda are assembled and new phylogenomic studies further resolve relationships within the group, it will be possible to precisely document this ancestral WGD event and clarify whether it might be associated with the high species diversification that occurred in Neogastropoda and allied families.

Hallinan and Lindberg [32] also postulated another WGD event in heterobranch gastropods. This WGD event occurred in the ancestor of Stylommatophora, an evolutionarily successful group of land snails and slugs, and thus might be associated with higher species diversification and even the water-to-land transition. The comparison of macrosynteny patterns between the genomes of *P. canaliculata* and 2 species of *Achatina* illustrates this WGD [35]. The genomes of the *Achatina* species have 31 chromosomes and sizes of 1.75–2.12 Gb [34, 35]. The macrosynteny relationships of this WGD indicate completely different evolutionary outcomes compared to the WGD event within Caenogastropoda [35], highlighting the role of contingency and the complexity of selective processes upon each WGD.

### Hox genes and other genes of interest for gastropod biology

A complete set of *hox* genes was located as a cluster in pseudochromosome 26 (Fig. 3C). The gene order in the *hox* cluster is similar to the one considered ancestral in gastropods and found in *L. gigantea* [37] and *C. squamiferum* [36] but includes differences affecting 2 regions: *hox1-hox5* and *lox5*-*post1* (Fig. 3C). According to the phylogeny, 2 equally parsimonious scenarios could render the observed pattern for the former region: (i) an inversion of *hox1-hox5* in the common ancestor of *A. immaculata, P. canaliculata*, and *L. ventricosus* followed by an inversion of the *hox5* gene in *L. ventricosus* and a reversal to the ancestral state in *P. canaliculata* (3 steps); and (ii) 2 independent inversions of *hox1-hox5* in *A. immaculata*, and of *hox1-hox4* in *L. ventricosus*, respectively, the latter followed by a translocation (3 steps). With regards to the *lox5*-*post1* region, an inversion is shared by *L. ventricosus* and *P. canaliculata*, indicating that it likely occurred in the common ancestor of Caenogastropoda.

The plesiomorphic state for *hox* expression in mollusks is represented by the staggered expression along the anterior-posterior body axis of 1 chiton [70]. Within Conchifera, temporal staggered expression is observed only during the early mid-stage trochophore larva of 1 scaphopod [71], in the embryo stage 19/20 of 1 cephalopod [72], and for anterior *hox* genes in the pre-torsional veliger of several gastropods [73]. This latter expression pattern is likely favored by gene co-linearity and subclustering of *hox1*-*5* genes as shown in pesudochromosome 26 of the *L. ventricosus* genome (and other gastropods [35, 37]). By contrast, in the other larval stages in cephalopods and gastropods, *hox* gene expression is not staggered along the anterior-posterior axis but occurs in distinct morphological structures [74].

A second *hox* cluster was located in pseudochromosome 33 (Fig. 3C). The presence of this second cluster further supports the presence of an ancestral WGD. It contains only 5 of the 11 *hox* genes present in gastropods. Because pseudochromosome 33 contained some gap regions, we searched for the missing genes in other pseudochromosome and in contigs not incorporated into scaffolds, but without success. Hence, we suggest that the missing genes were pseudogenized and eliminated after the WGD. In fact, *Achatina* shows a similar pattern with 1 complete and 1 partial *hox* cluster (Fig. 3C [35]).

With regards to the *parahox* gene cluster, it was only found in pseudochromosome 7 and contained *gsx* and *xlox* (also named *pdx*) genes but not the *cdx* gene, which was located neither in other pseudochromosomes nor in contigs not incorporated into scaffolds (Fig. 3C). Other gastropod genomes have the complete set of 3 *parahox* genes [31, 35–37]. As in *Achatina*, we found 1 *parahox* cluster, and thus the second cluster derived from both WGDs must have been secondarily lost in both species [35].

The genes involved in other important developmental pathways were also identified and located. The dorsal-ventral patterning of a protostome embryo is controlled by the dorsal expression of the decapentaplegic (*dpp*) gene and the ventral expression of the *chordin* and *noggin* genes [74]. Pseudochromosomes 4 and 11 had 1 copy each of the *dpp* gene (resulting from the WGD). The *chordin* gene was located in pseudochromosome 17 and the *noggin* gene has 2 inverted paralogs within 1 Mb distance in pseudochromosome 2. Left-right body asymmetry in gastropods is the result of larval torsion (rotation of the visceral mass, mantle, and shell by 180° with respect to the head and foot) and is governed by the expression first of the diaphanous-related formin (*ldia2*) gene [75] and later of *nodal* and *pitx* genes [76]. The *ldia2* gene was located in pseudochromosome 8; there is 1 copy of *nodal* in pseudochromosomes 1 and 2 (which result from the WGD); the *pitx* gene is in pseudochromosome 1. Stem cell proliferation, migration, and differentiation into tissues are the result of activation of various signaling proteins expressed by, e.g., hedgehog (*hh*) and *notch* genes [77]. One copy of the *hh* gene was located in pseudochromosome 26 and the *notch* gene was found in pseudochromosome 31. In gastropods the nitric oxide synthase controls the early stages in the development of shell gland, digestive gland, and kidney, as well as the induction of larval metamorphosis [78]. The gene encoding the nitric oxide synthase was located in pseudochromosome 25.

One of the most important features of a gastropod is the shell. Several genes including *engrailed* and *camlbp I* have been involved in the differentiation of a shell field distinct from the mantle tissue [79]. Two copies of the *engrailed* gene were located in pseudochromosome 26, and 1 each in pseudochromosomes 5, 7, and 33. The *camlbp I* gene was found in pseudochromosome 3. Although a large proportion of genes involved in generating shell structure are lineage-specific [80], some genes such as that encoding laminin [80] are commonly involved in the formation and biomineralization of the shell matrix across lineages. The genes encoding laminin subunits α, β, and γ were located in pseudochromosomes 6, 10, and 24, respectively. In the adult, the shell is often brightly colored owing to the presence of 3 types of pigments: carotenoids, tetrapyrroles, and melanins [81]. The biosynthesis of the melanin is controlled by the tyrosinase, an enzyme that catalyzes the oxidation of tyrosine into L-DOPA in the mantle [82], producing dark purple, brown, and black patterns in the pigmented shell layers of the shells of several mollusks (although apparently not in *Conus marmoreus* [83]). We identified and located the gene encoding tyrosinase in pseudochromosome 18.

As in other animals, sex determination is crucial in snails, which can have separate sexes or be hermaphrodite. No sex genes have been yet identified in gastropods. However, it is well documented that female snails of many gastropod species (particularly within the family Muricidae) can undergo masculinization when exposed to tributyltin (TBT), an environmental organic contaminant [84]. This process is called imposex, and although the exact mechanism of the endocrine disruption is not fully understood, it is clearly connected with the retinoid X receptor signaling pathway [85]. The exposure to TBT produces a local increase in the transcription levels of the *rxr* gene in the penis-forming field [84]. This gene was located in pseudochromosome 15.

Besides the mentioned key genes, we studied gene family expansions and contraction patterns in *L. ventricosus*. Comparisons of orthogroups among gastropods showed that patterns of expansion and contraction were more dynamic in terminal than internal branches (Supplementary Fig. S10). This pattern is likely the product of the sparse taxon sampling due to the few available gastropod genomes that hardly represent the vast gastropod diversity. More orthogroups expanded than contracted in all branches. Size change in the *L. ventricosus* lineage was significant for 443 orthogroups, of which 292 expanded and 151 contracted (Supplementary Table S5). A total of 231 (52%) of these orthogroups were of unknown function, although 168 rendered BLAST hits preferentially with other gastropods (Supplementary Table S5). Expanded orthogroups may represent cases of adaptation; of those with assigned function and ontology, several were related to chromatin and nucleic acid binding as well as cellular and metabolic processes; many to keratinization, calcification/shell formation, and mucus and adhesive protein secretion; and some related to ion transport, nervous system signaling, and hemostasis (Supplementary Table S5).

## Conclusions

Understanding the genetic basis of the evolutionary processes shaping the origin and diversification of venoms requires the comparison of venomous animal genomes, preferentially assembled at the chromosome level. Here, we provide a high-quality genome of a cone snail. There are >900 species of cone snails and this genome will serve as best reference for the assembly of other genomes within this group of marine venomous snails, opening the door to comparative analyses aimed at understanding the evolutionary origin and dynamics of conotoxin precursor gene families. Likewise, this resource will back up ongoing efforts in cataloguing toxin diversity through transcriptomic and proteomic analyses of cone snail venom glands and bolster the search for new drugs. In addition, it will be useful in characterizing the genetic consequences of a WGD event in the caenogastropod lineage.

During the review process of this article, the genome assembly of the vermivorous cone snail *Dendroconus betulinus* was reported [86], showing results highly congruent with our findings. The genome of this species was of similar size and assembled also into 35 major scaffolds. Up to 133 conotoxin precursor genes were identified and located in the different scaffolds [86]. As in the case of *L. ventricosus*, the ratio of conotoxin precursor genes and transcripts in *D. betulinus* was close to 1, and thus it was inferred that the high diversity of functional conotoxins is achieved at the post-translational level [86]. The assembly of 2 chromosome-level genomes of cone snails opens the door to fruitful comparative genomic studies aimed at further understanding the origin and evolution of conotoxin diversity.

## Methods

### Sampling

Adult specimens of *L. ventricosus* (NCBI:txid117992) were sampled in Olhão, Portugal. Once in resting stage, each individual was extracted from the shell with a sewing needle and dissected to obtain foot muscle, which was flash frozen in liquid nitrogen and stored at −80°C, as well as a piece of foot and the venom gland, which were preserved in RNAlater (Thermo Fisher Scientific, Waltham, MA, USA) and stored at −20°C.

### Flow cytometry

Flow cytometry was used to determine the haploid genome size of *L. ventricosus*. The genome of the German cockroach *Blattella germanica* (1C = 2.025 Gb [87]) was used as reference. Briefly, cells were isolated from the head of a cockroach and from the foot and proboscis of the cone snail [88], and incubated in lysis buffer LB01 [89] with 2% of tween, propidium iodide (50 μg/mL), and RNAse (40 μg/mL). After 10 minutes, the processed tissue was filtered using a nylon mesh of 20 μm. The DNA content of the diploid cells was determined through the relative G0/G1 peak positions of the stained nuclei using a Gallios flow cytometer (Beckman Coulter, Inc, Fullerton, CA, USA); the results were based on the average of 3 individuals, counting a minimum of 5,000 cells per individual.

### DNA extraction, library preparation, and sequencing

DNA isolation and genome sequencing, assembly, and annotation were carried out by Dovetail Genomics (Scotts Valley, CA, USA). High molecular weight (HMW) DNA was obtained from foot tissue stored at −80°C using Genomic-tip 20G (Qiagen, Toronto, ON, Canada) columns. DNA extractions were quantified using Qubit 2.0 Fluorometer (Life Technologies, Carlsbad, CA, USA) and their quality verified by gel electrophoresis. A total of 15 µg of HMW DNA from individual CV1492 (the shell was deposited as voucher in the MNCN collection under accession number MNCN 15.05/92196) was used to generate 4 PacBio SMRTbell libraries (∼20 kb). Sequencing was performed on 4 PacBio Sequel II Single Molecule, Real-Time (SMRT) cells. Sequencing yields were 52.2, 50.3, 45.0, and 45.1 Gb.

Three Chicago and 3 Hi-C libraries were prepared following [90] and [91], respectively. A total of 0.5 µg of HMW DNA from individual CV1495 (MNCN 15.05/92199) was used per library. Briefly, for Chicago libraries, HMW DNA (mean fragment length = 50 kb) was reconstituted into chromatin *in vitro* and fixed with formaldehyde. For Dovetail Hi-C libraries, chromatin was fixed in place with formaldehyde in the nucleus and then extracted. For both libraries, fixed chromatin was digested with DpnII, the 5′ overhangs filled in with biotinylated nucleotides, and free blunt ends were ligated. After ligation, crosslinks were reversed and the DNA purified and treated to remove biotin that was not internal to ligated fragments. The DNA was then sheared to ∼350 bp mean fragment size and sequencing libraries were generated using NEBNext Ultra enzymes and Illumina-compatible adapters. Biotin-containing fragments were isolated using streptavidin beads before PCR enrichment of each library. All 6 libraries were sequenced on an Illumina HiSeq X platform (paired-end, 2 × 151 bp). The read pairs produced for the Chicago libraries were 322, 162, and 277 Gb, and for the Dovetail Hi-C libraries were 145, 426, and 109 Gb.

### Genome assembly and scaffolding

Long reads sequenced in the 4 SMRT cells were *de novo* assembled using wtdgb2 [92]. This software computes the consensus haploid sequence of each contig and produces fewer false duplications than other assemblers [92], as tested in our genome assembly using Purge Haplotigs [93] and confirmed by the low number of duplicates in the BUSCO scores. The initial *de novo* assembly, shotgun long reads, Chicago library reads, and Dovetail Hi-C library reads were used as input data for HiRiSE^TM^, a software pipeline designed specifically for using proximity ligation data to scaffold genome assemblies [90]. An iterative analysis was conducted. First, Shotgun and Chicago library sequences were aligned to the draft input assembly using a modified SNAP read mapper [94]. The separations of Chicago read pairs mapped within draft scaffolds were analyzed by HiRiSE^TM^ to produce a likelihood model for genomic distance between read pairs, and the model was used to identify and break putative misjoins, to score prospective joins, and make joins above a threshold. After aligning and scaffolding Chicago data, Dovetail Hi-C library sequences were aligned and merged into scaffolds following the same method. After scaffolding, shotgun sequences were used to close gaps between contigs [90].

### RNA extraction, library preparation, and sequencing

The transcriptomes of the foot of individuals CV10 and CV19 (for wide gene annotation), as well as that of the venom gland of individual CV8 (for venom-related gene annotation), were determined. Each foot and venom gland tissue specimen was incubated independently in 300 µL of TRIzol LS reagent (Thermo Fisher Scientific, Waltham, MA, USA) and ground with ceramic beads in a Precellys Evolution tissue homogenizer. The solution was mixed with 60 µL of chloroform. After centrifugation (12,000*g* for 15 min at 4°C),  the aqueous phase was recovered and RNA precipitated in 1 volume of isopropanol and incubated overnight at −80 °C. The Direct-zol RNA miniprep kit (Zymo Research, Irvine, CA, USA) was used to purify 5–15 µg of total RNA following manufacturer's instructions.

Library construction and sequencing of the venom gland transcriptome was conducted at AllGenetics (Oleiros, Spain) in 2012 whereas foot transcriptomes were obtained at Sistemas Genómicos (Valencia, Spain) in 2016. Briefly, dual-indexed complementary DNA libraries (307–345 bp insert average size) were constructed for each sample using the TruSeq RNA Library Prep Kit v2 (Illumina, San Diego, CA). The quality and quantity of the libraries was determined with the TapeStation 4200, High Sensitivity assay, and by real-time PCR in LightCycler 480 (Roche), respectively. Libraries were split into 2 flowcells and sequenced in an Illumina HiSeq2000 (paired-end, 2 × 100 bp) platform.

### Transcriptome assembly

For each sample, RNA-seq raw reads were checked using FastQC (FastQC, RRID:SCR_014583) v0.10.1 [95]. Transcriptomes were assembled *de novo* using Trinity (Trinity, RRID:SCR_013048) v2.6.6 [96] with default parameters and the trimmomatic option activated. TransPi [61], which generates a consensus transcriptome assembly with different assemblers, was also used following the program instructions and using *k*-mer values of 25, 41, 53, and 75. Additionally, a reference-guided assembly of the venom gland transcriptome was performed. First, clean reads were mapped onto the final genome assembly with Hisat2 v2.2.0 [97]. Then, bam file outputs were sorted and used for a genome-guided assembly with Trinity 2.6.6 using the genome guided option, max_intron of 37,000, and all other parameters as default. Completeness of both assemblies was checked using BUSCO (BUSCO, RRID:SCR_015008) v4.0.6 [98] with the metazoa_odb10 gene set. The outputs of the 2 assemblies were merged and redundancy was eliminated with CD-HIT v4.5.4 [99] with default parameters to obtain the final transcriptome.

To estimate heterozygosity, we used GenomeScope (GenomeScope, RRID:SCR_017014) 2.0 [43]. The method is a *k*-mer–based statistical approach and owing to the error rate associated with long reads, PacBio raw data of the genome could not be used. Alternatively, Illumina pair-ended raw reads of the transcriptomes (CV8, CV10, CV19) were used to estimate heterozygosity. First, *k*-mer frequencies were estimated using jellyfish [100]. A range of *k*-mer sizes from 17 to 71 was analyzed. The different *k*-mer outputs were exported into *k*-mer count histogram files and uploaded into the GenomeScope 2.0 server. A *k*-mer size of 71 was selected as the best-fit model.

### Genome assembly quality evaluation

Quality assessment and general metrics of the final genome assembly were obtained with Quast (QUAST, RRID:SCR_001228) v5.0.2 [101]. An evaluation of coverage was conducted mapping subreads onto the final assembly using Minimap2 [102]. Potential sources of DNA contamination were checked with Blobtools (Blobtools, RRID:SCR_017618) v1.1 [103] using the NCBI entries of viruses, archaea, bacteria, fungi, nematodes, platyhelminthes, polychaetes, and human. NCBI entries for mollusks were used for the taxonomic identification of *L. ventricosus* contigs. A BLASTN search using the published mitogenome of *L. ventricosus* [104] as query was performed to detect and discard mitochondrial DNA. Completeness of the genome assembly was assessed with BUSCO v4.0.6 [98] in genome mode and using the metazoa_odb10 gene set.

### Transcript relative expression in venom gland versus foot

RNA-Seq clean reads were mapped with Bowtie2 (Bowtie 2, RRID:SCR_016368) [105] against the curated assembled transcripts and normalized in TPM (transcripts per kilobase million) using the function rsem-calculate-expression of the RSEM v1.2.31 package included in Trinity v2.6.6 [96]. TPMs derived from foot (CV10 and CV19) transcriptomes were combined and compared with those derived from the venom gland (CV8) transcriptome.

### Conotoxin precursor and other venom transcript annotation

The amino acid sequences of all conotoxin precursors and associated proteins of cone venoms available in GenBank release 236, Uniprot release 2020_02 (Uniprot Consortium 2017), and ConoServer release 02–04-2020 [23] were downloaded on 4 February 2020 to construct a custom reference database. Redundancy in database was eliminated using CDHIT v4.5.4 with a 95% identity threshold. Transcripts encoding conotoxin precursors and associated proteins were identified by BLASTX similarity searches of the transcripts against the above reference database (e-value of 1 × 10^−5^). TBLASTX similarity searches against the NCBI NR database and manual inspection were performed in order to discard false-positive hits (hits not corresponding to canonical conotoxins) or assembly artifacts (in low-coverage terminal positions and chimeras). Highly truncated (>55% of the estimated total length) peptide sequences were removed to produce the final working list of conotoxin precursors and associated proteins. The 3 domains of the predicted conotoxin precursors (signal, propeptide, and mature) and the cysteine frameworks of the mature functional peptides were identified using the Conoprec tool [23]. Assignment of precursors to different protein superfamilies was based on the 2 highest scoring full-length conotoxin precursor hits in the BLAST results, as well as taking into account the percentage of sequence identity (>70%) to the highly conserved signal region.

### Genome annotation

Reference libraries of repetitive sequences were generated *de novo* from the genome assembly using RepeatModeler (RepeatModeler, RRID:SCR_015027) v2.0.1 [106], RECON v1.08 [107], and RepeatScout (RepeatScout, RRID:SCR_014653) v1.0.6 [108]. The custom libraries were used to identify, quantify, and mask repeat elements with RepeatMasker (RepeatMasker, RRID:SCR_012954) 4.1.0 [109].

Gene predictions were generated using AUGUSTUS (Augustus, RRID:SCR_008417) v2.5.5 [110]. The coding sequences of the genomes of 3 gastropods, *Aplysia californica* (GCF_000002075.1)*, Biomphalaria glabrata* (GCA_000457365.1), and *Lottia gigantea* (GCA_000327385.1); 3 bivalves, *Crassostrea gigas* (GCA_902806645.1)*, Crassostrea virginica* (GCA_002 022 765.4), and *Mizuhopecten yessoensis* (GCA_002113885.2); and 1 cephalopod, *Octopus bimaculoides* (GCA_001194135.1) were used to train the *ab initio* model for *L. ventricosus*. Three rounds of prediction optimization were performed. The same coding sequences were also used to train an independent *ab initio* model with SNAP v2006-07-28 [111]. Newly generated RNA-sequencing reads from *L. ventricosus* and from the foot (SRX984185), mantle (SRX984179), nervous ganglia (SRX980532), and osphradium (SRX984173) transcriptomes of *P. consors* [29] were mapped onto the genome using STAR v2.7 [112]. Resulting bam files were used to generate intron hints with bam2hints in AUGUSTUS. The AUGUSTUS and SNAP models along with intron-exon boundary hints provided from RNA-Seq were used as input to MAKER (MAKER, RRID:SCR_005309) v3.01.01 pipeline [113] to predict for genes in the repeat-masked reference genome. To help guide the prediction process, Swiss-Prot peptide sequences from the UniProt database were downloaded and used in conjunction with the protein sequences from mollusks used for gene training as peptide evidence in the Maker pipeline. To help assess the quality of the gene prediction, AED (annotation edit distance) scores were generated for each of the predicted genes as part of the MAKER pipeline. If multiple models predicted by SNAP and AUGUSTUS overlapped, only the one with the lowest AED was retained in the final annotation set. Genes were further characterized for their putative function by performing a BLAST search of the peptide sequences against the UniProt database. Transfer RNAs (tRNAs) were predicted using the software tRNAscan-SE (tRNAscan-SE, RRID:SCR_010835) v 2.05 [114].

### Gene family manual annotation

#### Venom-related genes in the genome

A custom non-redundant database was constructed including the nucleotide sequences of the curated list of conotoxins, hormones, and other proteins derived from the transcriptome of *L. ventricosus* (see above) plus the nucleotide sequences of additional conotoxins, hormones, and related venom proteins derived from the transcriptomes of 13 closely related cone snail species from Cabo Verde and Senegal [9]. A BLASTN search against the genome assembly and a TBLASTN (e-value of 1 × 10^−5^) search of the genome assembly against the translated conotoxin database were performed. BLAST outputs were transformed to GFF3 file format and loaded into Geneious (Geneious, RRID:SCR_010519) v2020.1.2 [115]. Each hit was manually curated by adjusting intron-exon GT/AG junctions and by comparing exons with the original transcripts to detect any broken open reading frame and possible missing exons. Venom-related gene annotations are reported in a separate GFF3 file (Supplementary File S3).

#### *Hox* and *parahox* genes

Mollusk *hox* proteins available in NCBI and the HMM profile for the homeodomain (PFAM: PF00046) were fed into BITACORA v1.2.1 [116] to identify members of the *hox* gene family previously not detected in the automated annotation. In addition, the genome was searched using TBLASTN and all available mollusk *hox* and *parahox* proteins to identify any missing homolog. The identity of *hox* and *parahox* genes was confirmed upon multiple sequence alignment MAFFT einsi (MAFFT, RRID:SCR_011811) [117] and maximum likelihood inference under the BIC-selected best-fit model in IQTREE v1.6.12 [118]. *Hox* and *parahox* gene annotations are reported in a separate GFF3 file (Supplementary File S4).

#### Other genes of interest

Identification and location of genes involved in development, shell formation, color, and sex was conducted through TBLASTN searches (e-value of 1 × 10^−5^) of representative NCBI entries (mostly gastropod orthologues) of each gene against the 35 pseudochromosomes. Hits were converted to GFF3 files and loaded into Geneious v2020.1.2 [115] to manually reconstruct the exon-intron boundaries.

### Synteny and whole-genome duplication

Conserved synteny between *L. ventricosus* and *P. canaliculata* pseudochromosomes was inferred using pairs of 1:1 and 1:2 orthologs obtained with Orthofinder (OrthoFinder, RRID:SCR_017118) v2.3.11 [119]. Synteny plots were generated with the shinyCircos package [120]. To simplify plotting, short links <1 kb were filtered out and adjacent links (within 10 Mb) were merged using the bundlelinks tool [120]. The presence of WGD was also assessed using WGDdetector [121], which measures the synonymous rates of substitution (*Ks*) between pairs of paralogs. The *Ks* method assumes an L-shaped distribution of *Ks* for diploid species and additional peaks in *Ks* correspond to pairs of paralogs with similar synonymous divergences expected under a shared origin time by WGD. The *Ks* distances were plotted with the R package ggplot2 [122].

### Patterns of gene family evolution

We used CAFE (Computational Analysis of gene Family Evolution, RRID:SCR_018924) v5.0 [123] to infer expansion and contraction of gene families in the *L. ventricosus* genome. Orthogroups were inferred with Orthofinder v2.3.11 [119] using all annotated proteins from 6 gastropod genomes (*A. californica, B. glabrata, Elysia chlorotica, L. ventricosus, L. gigantea*, and *P. canaliculata*). A dated tree was built from the current consensus on gastropod phylogeny [124] and median divergence times from the timetree.org database [125]. Gene family expansion and contraction patterns were inferred for 11,990 orthogroups that were present at the tree root, assuming a global rate for gene family size change (λ) and a uniform gene family size distribution at the tree root. Those orthogroups containing *L. ventricosus* genes annotated as related to TEs were discarded from further study. The remaining orthogroups were functionally characterized using the automated genome annotation, as well as by a similarity search of their sequences against the NCBI NR database using diamond v0.9.9 [126] with an e-value threshold of 1e^−6^.

## Data Availability

Final assembly and original PacBio assembly, as well as annotation files, predicted transcript and protein sequences, and bioinformatics supporting information, were deposited in the GigaScience database GigaDB [127]. Additionally, assembly, PacBio subreads, and transcriptome raw data are available in NCBI and can be accessed with bioproject No. PRJNA678883. The final assembly can be accessed with JAFLJL000000000; PacBio subreads, with SRR13994261–SRR13994264; and RNA-sequencing raw reads, as follows: CV8: SRR13740844, CV10: SRR13757741, CV19: SRR13770976. The voucher (shell) of the specimen used for sequencing the genome is deposited in the MNCN collection under accession number MNCN 15.05/92196.

## Additional Files

**SupplementaryFigure S1**: DNA sources in the *L. ventricosus* genome. Potential sources of DNA contamination were checked with Blobtools v1.1 using the NCBI entries of viruses, archaea, bacteria, fungi, nematodes, platyhelminthes, polychaetes, and human. NCBI entries for mollusks were used for the taxonomic identification of *L. ventricosus* contigs.

**Supplementary Figure S2**: Link density histogram showing the 35 larger scaffolds of the *Lautoconus ventricosus* genome assembly. The x- and y-axes give the mapping positions of the first and second read in the read pair, respectively, grouped into bins. The color of each square gives the number of read pairs within that bin. White vertical and black horizontal lines denote borders between scaffolds. Scaffolds <1 Mb were excluded. Figure provided by Dovetail genomics.

**Supplementary Figure S3**: Pseudochromosome sizes. Histogram showing the sizes (in base pairs) of the 35 largest scaffolds in the *Lautoconus ventricosus* genome assembly.

**Supplementary Figure S4**: Relative expression of venom-related transcripts in the venom gland (CV8; blue) and the foot (CV10 and CV19; orange). Histograms show the relative expression, normalized in transcripts per million (TPM), of conotoxin precursor, hormone, and other venom-related protein transcripts.

**Supplementary Figure S5**: Annotation of venom-related genes in the 35 pseudochromosomes of the *L. ventricosus* genome assembly. A pie chart shows the number of venom-related genes identified on the basis of BLAST searches with the *L. ventricosus* venom gland transcriptome (CV8) and the venom gland transcriptomes of closely related cone species endemic to Cabo Verde [9] as queries (dark and light colors, respectively). The number of automatically and manually completed genes as well as of partial genes (with >1 exon) and single exons is shown. A second pie chart shows how several genes were manually completed with sequences from smaller scaffolds, contigs, and reads. A total of 12 genes were completed by forcing the combination of misassembled, scattered hits, i.e., distantly located (sometimes separated by other genes) or reciprocally inverted within a pseudochromosome.

**Supplementary Figure S6**: Distribution of complete venom-related genes in the 35 pseudochromosomes. The regression analysis indicates that the number of venom-related genes was independent of the length (in base pairs) of the pseudochromosome.

**Supplementary Figure S7**: Correspondence between 3-exon conotoxin precursor genes and protein domains. The boundaries of the 3 exons of the genes and the 3 domains (signal, propeptide, and mature) of the encoded protein were compared. An example in which these boundaries did not coincide is shown.

**SupplementaryFigure S8**: Intron lengths of 3-exon conotoxin precursor genes. Violin plots estimated using the package Seaborn (https://seaborn.pydata.org/), showing the probability density of the length (in base pairs) of introns 1 (dots in blue) and 2 (dots in red).

**Supplementary Figure S9**: Whole-genome duplication in the genome of *L. ventricosus*. Conserved synteny between each of the 14 *P. canaliculata* pseudochromosomes (red numbers) and the 35 *L. ventricosus* pseudochromosomes (black numbers) was inferred using pairs of 1:1 and 1:2 orthologs obtained with Orthofinder v2.3.11. Synteny plots were generated with shinyCircos.

**Supplementary Figure S10**: Significant expansion and contraction of gene families in gastropods. A dated tree was built from the current consensus on gastropod phylogeny [124] and median divergence times from the timetree.org database [125]. A total of 11,990 orthogroups were inferred with Orthofinder v2.3.11 using all annotated proteins from 6 gastropod genomes. Gene family significant expansions and contractions were inferred using CAFE v5.0. Those orthogroups containing genes annotated as related to transposable elements were discarded from further study. The remaining orthogroups were mapped onto the phylogeny.

**SupplementaryTable S1**: Assembly metrics for the *L. ventricosus* genome.

**SupplementaryTable S2**: Transcriptomes of the venom gland (CV8) and foot (CV10 and CV19) of *L. ventricosus*.

**Supplementary Table S3:**Relative expression (ratio of transcripts per million -TPMs- and %) of venom-related protein superfamily transcripts in the venom gland (VG) and the foot (F) of *L. ventricosus*.

**Supplementary Table S4**: Venom-related (conotoxin precursor, hormone, other) genes in the 35 pseudochromosomes of the *L. ventricosus* genome assembly.

**Supplementary Table S5:**Gene family dynamics (significant expansions and contractions) in th**e***L. ventricosus* genome.

**Supplementary File S1: Alignments of conotoxin precursors, hormones, and other venom-related proteins derived from the** *L. ventricosus* transcriptomes (CV8 from venom gland; CV10 and CV19 from foot) with homologues from closely-related cone snail species from West Africa (codes V_ and A_; Abalde et al, 2020) and from other cone species available in GenBank (the accession number follows the species name).

**Supplementary File S2**: Venom-related genes in the *L. ventricosus* genome. Annotation of conotoxin precursors, hormones, and other venom-related protein genes ordered by their location in the 35 pseudochromosomes. The nucleotide sequence of the gene (partitioned in exons) and the amino acid sequence of the derived protein are given. Coordinates of the gene sequence are provided (the sense of the arrow indicates forward or reverse transcription). Alignment with transcripts from the venom gland transcriptomes of *L. ventricosus* (CV8) and closely-related cone snail species from West Africa (codes V_ and A_; Abalde et al, 2020) are shown.

**Supplementary File S3**: Venom-related gene annotations reported in a GFF3 file.

**Supplementary File S4**: *Hox* and *parahox* gene annotations reported in a GFF3 file.

## Abbreviations

BLAST: Basic Local Alignment Search Tool; bp: base pairs; Gb: gigabase pairs; HMW: high molecular weight; kb: kilobase pairs; MAFFT: Multiple Alignment using Fast Fourier Transform; Mb: megabase pairs; NCBI: National Center for Biotechnology Information; SMRT: Single Molecule Real-Time; TBT: tributyltin; TE: transposable element; TPM: transcripts per million; WGD: whole-genome duplication.

## Competing Interests

The authors declare that they have no competing interests.

## Funding

This work was funded by the Spanish Ministry of Science and Innovation (CGL2016-75255-C2-1-P [AEI/FEDER, UE] and PID2019-103947GB-C22/AEI/10.13039/501100011033 to R.Z.; BES-2017–081195 to J.R.P.-B.; BES-2014–069575 to S.A.; IJCI-2016–29566 to I.I.). I.I. acknowledges the support from the European Research Council during the latest stages of the project (Grant Agreement No. 852725; ERC-StG “TerreStriAL” to Jan de Vries, University of Goettingen).

## Authors' Contributions

R.Z. conceived the study and designed the experiments and analyses. M.J.T. and C.M.L.A. obtained the individuals, performed sample dissections, and provided information on cone snail biology. J.R.P.-B. and S.A. worked on venom gland and foot comparative transcriptomics. J.R.P.-B. and I.I. performed genome analyses and manual gene annotations. R.Z. wrote the manuscript initial draft, and all authors read, revised, and approved the manuscript final version.

## Supplementary Material

giab037_GIGA-D-21-00040_Original_Submission

giab037_GIGA-D-21-00040_Revision_1

giab037_Response_to_Reviewer_Comments_Original_Submission

giab037_Reviewer_1_Report_Original_Submission SÃ©bastien Dutertre -- 3/8/2021 Reviewed

giab037_Reviewer_1_Report_Revision_1 SÃ©bastien Dutertre -- 4/1/2021 Reviewed

giab037_Reviewer_2_Report_Original_Submission Kevin Kocot -- 3/8/2021 Reviewed

giab037_Reviewer_2_Report_Revision_1 Kevin Kocot -- 4/14/2021 Reviewed

giab037_Supplemental_Files
