# Supplementary material for: The genome of the venomous snail Lautoconus ventricosus sheds light on the origin of conotoxin diversity
Source: Gigascience. 2021 May 25;10(5):giab037. doi: 10.1093/gigascience/giab037 (PMC8152183; doi:10.1093/gigascience/giab037)
Supplement: giab037_GIGA-D-21-00040_Revision_1 [file giab037_giga-d-21-00040_revision_1.pdf]

GigaScience

The genome of the venomous snail Lautoconus ventricosus sheds light on the origin of conotoxin diversity

--Manuscript Draft--

**Manuscript Number:**	GIGA-D-21-00040R1
**Full Title:**	The genome of the venomous snail Lautoconus ventricosus sheds light on the origin of conotoxin diversity
**Article Type:**	Research
**Funding Information:**	Ministerio de Ciencia e Innovación (PID2019-103947GB-C22/AEI/10.13039/501100011033) — Prof. Rafael Zardoya<br>Ministerio de Ciencia e Innovación (CGL2016-75255-C2-1-P [AEI/FEDER, UE]) — Prof. Rafael Zardoya<br>Ministerio de Ciencia e Innovación (BES-2017-081195) — Mr José Ramón Pardo-Blas<br>Ministerio de Ciencia e Innovación (BES-2014-069575) — Dr. Samuel Abalde<br>Ministerio de Ciencia e Innovación (IJCI-2016-29566) — Dr. Iker Irisarri<br>H2020 European Research Council (StG) (852725) — Not applicable
**Abstract:**	Background: Venoms are deadly weapons to subdue prey or deter predators that have evolved independently in many animal lineages. The genomes of venomous animals are essential to understand the evolutionary mechanisms involved in the origin and diversification of venoms. Results: Here, we report the chromosome-level genome of the venomous Mediterranean cone snail, Lautoconus ventricosus (Caenogastropoda: Conidae). The total size of the assembly is 3.59 Gb; it has high contiguity (N50= 93.53 Mb) and 86.6 of the genome assembled into the 35 largest scaffolds or pseudochromosomes. Based on venom gland transcriptomes, we annotated 262 complete genes encoding conotoxin precursors, hormones, and other venom-related proteins. These genes were scattered in the different pseudochromosomes and located within repetitive regions. The genes encoding conotoxin precursors were normally structured into three exons, which did not necessarily coincide with the three structural domains of the corresponding proteins. Additionally, we found evidence in the L. ventricosus genome for a past whole genome duplication event by means of conserved gene synteny with the Pomacea canaliculata genome, the only one available at the chromosome level within Caenogastropoda. The whole genome duplication event was further confirmed by the presence of a duplicated hox gene cluster. Key genes for gastropod biology including those encoding proteins related with development, shell formation, and sex were located in the genome. Conclusions: The new high-quality L. ventricosus genome should become a reference for assembling and analyzing new gastropod genomes and will contribute to future evolutionary genomic studies among venomous animals.
**Corresponding Author:**	Rafael Zardoya, PhD<br>Museo Nacional de Ciencias Naturales-CSIC<br>Madrid, Madrid SPAIN
**Corresponding Author Secondary Information:**	
**Corresponding Author's Institution:**	Museo Nacional de Ciencias Naturales-CSIC
**Corresponding Author's Secondary Institution:**	
**First Author:**	José Ramón Pardo-Blas
**First Author Secondary Information:**	
**Order of Authors:**	José Ramón Pardo-Blas

Powered by Editorial Manager® and ProduXion Manager® from Aries Systems Corporation

	Iker Irisarri
	Samuel Abalde
	Carlos Manuel Lourenço Afonso
	Manuel J. Tenorio
	Rafael Zardoya, PhD
**Order of Authors Secondary Information:**	
**Response to Reviewers:**	RESPONSE TO REVIEWERS<br><br>Editor<br>I'd encourage you to upload additional supporting data, such as software input/output files, "intermediate assemblies", annotations etc, as mentioned by the referee, to our FTP server. We will release the supporting data set with a stable Digital Object Identifier via our repository GigaDB, alongside the paper.<br><br>Following the suggestion of the reviewer we have uploaded in GigaDB, the original PacBio assembly and bioinformatics commands (the final assembly, annotation files, predicted transcript and protein sequences were already in GigaDB). We have also updated the Readme and md5sum files with the new data.<br><br><br>Reviewer #1<br>This manuscript, entitled « The genome of the venomous snail Lautoconus ventricosus sheds light on the origin of conotoxin diversity » by Pardos-Blas et al., provides a high quality chromosome-level genome of a Conidae. It is very unfortunate for the authors that another cone snail genome has been published the very next day I received this paper for review (Peng C et al., Cell Discovery 2021). In any case, while taking a bit of the novelty out, I see these two papers as complementary rather than directly competing. Here, the genome data were complemented with venom gland transcriptome data and overall, the authors have performed a detailed analysis of their genome and transcriptome data. I enjoyed reading this manuscript, which is very well written, and nicely illustrated. Congratulations to the authors for a remarkable achievement. I recommend publication as I only have some minor comments.<br><br>We thank the reviewer for the positive feedback. As the referee mentions, during the review process, the genome assembly of Dendroconus betulinus was published. We now acknowledge this paper after the conclusions and mention the congruency found between their and our results. The following paragraph was added:<br><br>"During the review process of this article, the genome assembly of the vermivorous cone snail Dendroconus betulinus was reported [76], showing results highly congruent with our findings. The genome of this species was of similar size and assembled also into 35 major scaffolds, and up to 133 conotoxin precursor genes were identified and located in the different scaffolds [76]. As in the case of L. ventricosus, the ratio of conotoxin precursor genes and transcripts in D. betulinus was close to one, and thus it was inferred that the high diversity of functional conotoxins is achieved at the post-translational level [76]. The assembly of two chromosome-level genomes of cone snails opens the door to fruitful comparative genomic studies aimed at further understanding the origin and evolution of conotoxin diversity."<br><br>- Just a general comment: I wonder why the authors did not use the same specimen for both genomics and transcriptomics ? Cone snail venoms are notoriously hypervariable, including significant intra-specific variations. This is particularly true for Conus ventricosus (as demonstrated by Romeo C et al., J Sep Sci, 2008, this should be cited), and therefore I am wondering how much of this variation may account for the discrepancy between the number of conotoxin genes vs transcripts. The authors may want to comment on this.<br><br>The venom gland transcriptomes were obtained from specimens captured in 2012 in Olhão, Portugal. At that time the possibility of sequencing the genome was not anticipated and the body was preserved in ethanol 100%, which does not render HMW DNA. The PacBio reads and the llumina/ HiC data were obtained from specimens

captured in 2017 also in Olhão, Portugal. We used large quantities of tissue (including the venom gland) to purify HMW DNA and during three years we solved many technical problems to obtain the genome. Focus on that, we neither preserve apart the venom glands nor used them to obtain transcriptomes, which should have been the best strategy, as we know now. The reviewer is right and not only conotoxins may vary among individuals (although many reported variations are not at the sequence level but at the post-translational modifications of the peptide) but also there are important levels of heterozigosity for all genes. It is possible that part of the discrepancy between the number of conotoxin genes versus transcripts may be due to inter-individual variation, which has been reported among L. ventricosus populations (Romeo et al., 2008). Now, we mention that possibility in the main text and also cite the suggested paper.

- I agree with the title that this work sheds light on conotoxin diversity, but to me, another unanswered question remains: the origin(s) of conotoxin genes. For hormone-like venom peptides (conoinsulin, conopressin…etc), duplication/neofunctionalization of the endogenous hormone gene can be put forward as the most likely event for their presence in the venom gland, but to my knowledge, nothing « endogenous » ressembles the disulfide-rich conotoxins. Where do they come from?

As we mention in the text "To explain the evolutionary origin … it has been suggested that toxin genes could emerge either through gene duplication and adaptive neofunctionalization of physiological genes in the venom gland coupled with reduction of expression levels in other tissues or alternatively by subfunctionalization through neutral evolution and restriction to the venom gland. This statement includes conotoxins and hormones. Given the fast evolutionary rates of conotoxins it is not surprising that it is very difficult to identify the original endogenous proteins that evolved into conotoxins, but the fact that their function is to inhibit ion channels in the neuromuscular system of the prey, point to an origin from (or a convergence to) neuropeptides.

- Interestingly, the authors find conotoxins genes in the foot transcriptome, although expressed at low levels. They comment page 3 that « this is the first report of conotoxin expression outside the venom gland in cone snails ». Not quite true, as Biggs JS et al. (Toxicon. 2008) have reported alpha-conotoxins specifically expressed in the salivary gland of Conus pulicarius, and more recently, Dutertre S et al. (Nat Comm, 2014) have reported conotoxin expression in the radular sac of Conus geographus. Nonetheless, this finding raises several questions. First, are those conotoxin transcripts the same as those found in the venom gland (Dutertre S et al. found different transcripts in the radular sac, possibly pseudogenes) ? Second, I understand that it is highly speculative, but could the authors discuss the putative role of these transcripts in the foot? They are expressed at low levels, but could they have an antimicrobial role (like many molluscs, the foot of cone snail produces mucus, and such antimicrobial agents have been found in other gastropods)? Finally, some hormone-like transcripts were found more highly expressed in the foot compared to the venom gland, could these represent the endogenous hormones?

The reviewer is right, and the expression of few conotoxin transcripts had been reported in other cone species. We now cite the two cases mentioned by the reviewer. In addition, we have added a Venn diagram to Supplementary table S2 to show the exact transcripts that were shared by all transcriptomes or by each of the foot transcriptomes and the venom gland transcriptome. Nevertheless, given the variability of the conotoxin precursors, in the main text we discuss comparisons at the superfamily level rather than transcript-by-transcript. Finally, following the suggestion of the reviewer, we now mention the possibility that the conotoxin transcripts in the foot may have antimicrobial activity as reported for some conotoxins found in other cone species (Figueroa-Montiel et al. 2018 and Bernáldez-Sarabia et al. 2019). As suggested by the reviewer, we now state: "these hormones and proteins may be endogenous, having a physiological function common to different tissues and not restricted to the venom gland".

Typos and other small errors:

- page 3 : I would invert the order (smallest to largest) of the sentence : « …from 184 to 44 Mb ».

- page 4, last para : « The gene structures of found… ».
- page 6, top left : « The comparison…illustrate… » should be « illustrates ».
- page 7, second para : « …have been involved IN the differenciation… ».
- page 7, third para : « …have separate sexes OR be hermaphrodite. »

All changed as suggested!

Reviewer #2
In this study, Ramón Pardos-Blas et al. report a chromosome-level geome assembly for the Mediterranean cone snail. This is an impressive achievement given the large (3.59 Gb) size of this genome. I think this genome will be a valuable resource for the molluscan genomics community, especially for those interested in gastropods (as only one high-quality caenogastropod genome is available) and venom evolution. I do have a number of mostly minor questions and comments that I think should be addressed.

We thank Kevin Kocot for his insightful feedback

It is stated that the genome annotations contain 89.2% of the BUSCO metazoan_odb10 genes but it is not stated what the BUSCO score of the scaffolded genome assembly itself is in the main text. This would be helpful in assessing the completeness of the gene models relative to the genome assembly. Likewise please also report all of the BUSCO metrics (e.g., percent fragmented and duplicated) for both the scaffolded genome and gene models in the main text.

We thank the reviewer for pointing this issue: Actually, we made a mistake here and what we were reporting was the BUSCO metric for the scaffolded genome and not for the gene models. We have corrected that and provided the metrics for the genome assembly and the gene models including percentages of complete duplicated and fragments. For the genome: complete single-copy, 82%; complete duplicated, 2.9%; fragmented, 4.3%. For the gene models: complete single-copy, 31.2%; complete duplicated, 0.4%; fragmented, 21.7%.

The somewhat low BUSCO score for the gene models is attributed to "likely due to the much larger genome size of L. ventricosus" (with respect to other gastropods with higher BUSCO scores). However, the rationale behind this statement is not explained.

This statement was deleted

On page 3, it is stated that "The main methodological limitation that may explain the missing loci would be the error rate associated to the PacBio CLR sequencing technology (15% [47]), which despite partially corrected by coverage, would hamper BLAST similarity searches." Was any attempt made to polish the genome with the short reads using e.g., Pilon or POLCA?

As explained in the materials and methods section, the assembly of the genome was performed based exclusively on the PacBio long reads from one individual. The Illumina reads from another individual were used for the Chicago and HiC scaffolding. Due to the heterozygosity between individuals, it was not possible to use these Illumina reads to polish the PacBio-based genome assembly.

What is the heterozygosity of the genome? This should be reported in the main text.

In order to estimate heterozygosity, we used GenomeScope 2.0. However, in the original paper of the software (Vurture et al., 2017), the authors warned: “Oxford Nanopore or Pacific Biosciences, which currently average 5–20% error, are not supported as an error will occur on average every 5–20 bp and thus infer with nearly every k-mer”. Alternatively, we used the Illumina raw reads of the transcriptomes (CV8, CV10, CV19) to estimate it. The final values of heterozygosity were 1.05-1.08%. This is now mentioned and discussed in the main text.

Was any attempt made to purge redundant haplotigs from the initial long-read assembly prior to scaffolding?

As explained in Materials & Methods, the long-read assembly was obtained using

Powered by Editorial Manager® and ProduXion Manager® from Aries Systems Corporation

wtdbg2. This software follows the Overlap Layout Consensus methodology, which computes the consensus haploid sequence of each contig. Wtdbg2 produces less false duplications than other assemblers (Ruan and Li, 2020), so no attempt to purge duplicates was made. Nonetheless, motivated by the suggestion of the reviewer, we ran Purge Haplotigs (Roach et al., 2018) to detect false duplications and obtained a single peak in the read-depth histogram indicating low levels of duplicates, which were also evidenced by the BUSCO results (2.9% duplicates).

On page 3 it is stated that "The percentage of complete venom-related loci detected is considerably lower than expected according to general BUSCO results (89.2%). The extra 15% of transcripts of the transcriptome without a gene counterpart in the genome could be isoforms that could be produced naturally during expression or generated as artifacts during transcriptome assembly." Perhaps the Oyster River Protocol or this (admittedly unpublished) approach should be tested?
https://www.biorxiv.org/content/10.1101/2021.02.18.431773v1

Following the suggestion of the reviewer, we ran TransPi (Rivera-Vicéns et al., 2021). This program uses various assemblers and k-mers to generate a non-redundant consensus de novo transcriptome. The resulting L. ventricosus venom gland transcriptome had BUSCO scores (C:69.0% [S:60.8%,D:8.2%], F:9.7%, M:21.3%, n:954) that were very similar to those obtained using Trinity alone (C:67.6% [S:57.5%,D:10.1%], F:12.5%, M:19.9%, n:954).

Conotoxin transcripts were detected in the foot tissue transcriptome. In the methods, please indicate if these RNA extractions were performed at the same time or not and how the transcriptome libraries were split between the two HiSeq 2000 flowcells. Is it possible there was laboratory contamination or 'bleed through' where some barcodes in the venom gland library were mis-read as barcodes for the foot library, resulting in some reads 'bleeding through' into the foot transcriptome data?

The processing of the venom gland and foot samples was done in different years, and therefore the 'bleeding through' suggested by the reviewer was impossible. The text now reads “Library construction and sequencing of the venom gland transcriptome was conducted at AllGenetics (Oleiros, Spain) in 2012 whereas foot transcriptomes were obtained at Sistemas Genómicos (Valencia, Spain) in 2016.”

I noticed ferritin is highly expressed in the foot. Any idea why?

The reviewer is right that ferritin shows high expression levels in the foot. In this regard, we have added the following comment to the text: “This is a protein that generally regulates the storage and release of iron, and has been related to the incorporation of iron into the radula in some chitons and limpets, and into the shell in the pearl oyster”

I would encourage the authors to make the initial PacBio assembly and as many of the exact commands used and key input/output files for the programs they ran available via e.g., Figshare or Dryad.

Following the suggestion of the reviewer we have uploaded in GigaDB, the original PacBio assembly and bioinformatics commands (the final assembly, annotation files, predicted transcript and protein sequences were already in GigaDB). We have also updated the Readme and md5sum files with the new data.

The methods state (on page 8) that "After scaffolding, shotgun sequences were used to close gaps between contigs." Not enough detail is provided here to assess what was done.

Powered by Editorial Manager® and ProduXion Manager® from Aries Systems Corporation

The procedure is explained in detail in Putnam et al. 2016. We now cite that reference at the end of the sentence. The authors say: “HiRise can use paired-end shotgun reads to close some of the gaps of unknown sequence created when scaffolds are joined based on Chicago read pairs. Groups of reads localized by SNAP alignment to the vicinity of each such gap are passed to “marauder,” the gap-closing module of meraculous, which returns a gap-closing sequence when a unique closure can be inferred by local k-mer walking.

The methods state (on page 9) "Only models that were predicted by both SNAP and AUGUSTUS were retained in the final annotation set." How was this ensured?

The reviewer is right and the sentence was misleading. We now state: “If multiple models predicted by SNAP and AUGUSTUS overlapped, only the one with the lowest AED was retained in the final annotation set”

The statement on the availability of supporting data is not clear on whether the predicted transcripts and proteins from the automated genome annotation are made available as fasta files. Please provide these as well.

We have modified the statement. “Final assembly, original PacBio assembly, as well as annotation files, predicted transcript and protein sequences, and bioinformatics supporting information were deposited in GigaDB. Additionally, Assembly, PacBio subreads and transcriptome raw data were deposited at NCBI under the bioproject number PRJNA678883. Final assembly (JAFLJL000000000); PacBio subreads (SRR13994261-SRR13994264); RNAseq raw reads (CV8: SRR13740844, CV10: SRR13757741, CV19: SRR13770976).”

Minor points:
Correct "preys" to "prey" in the abstract.

Corrected as suggested

Confirm that all instances of "L. ventricosus" are italicized.

Confirmed!

Page 2, L column, first paragraph: "The latter…" - Does this refer to snakes?

Now, it reads “The snakes…”

Page 2, R column, first paragraph: "newly long-read assembled genome" - this is a bit awkward as written.

“Long-read” was deleted

Page 4, L column, first full paragraph: Add space between "31" and "were"

Added!

Page 5, R column, third paragraph: Please clarify what is meant by "an evolutionary successful group of land snails and slugs" here. Pulmonata? All Heterobranchia?

We now mention that the group is Stylommatophora

Page 7, L column, second paragraph: The detection of the camlbp I gene is noted but its significance is not explained in detail.

Actually, its function is explained together with that of engrailed genes in a previous sentence: “Several genes including engrailed and camlbp I have been involved in the differentiation of a shell field distinct from the mantle tissue”

Page 7, L column, third paragraph: There is an English problem here: "sexes of be hermaphrodite"

	Changed to “sexes or be hermaphrodite”<br><br>Page 8, L column, RNA extraction, library preparation, and sequencing section: Correct "grinded" to "ground"<br><br>Changed as suggested<br><br>Page 8, R column, last section: I suggest changing "Genome automated annotation" to just "Genome annotation"<br><br>Changed as suggested<br><br>Figure 1: The caption does not explain the y-axis for the distributions of protein-coding genes and repetitive elements.<br><br>Now, we explain in the caption that the y-axis for the distributions of protein-coding genes and repetitive elements correspond to percentage of genes per megabase (normalized to 40 genes and to 6,000 repetitive elements).
Additional Information:	
Question	Response
Are you submitting this manuscript to a special series or article collection?	No
**Experimental design and statistics**<br><br>Full details of the experimental design and statistical methods used should be given in the Methods section, as detailed in our Minimum Standards Reporting Checklist. Information essential to interpreting the data presented should be made available in the figure legends.<br><br>Have you included all the information requested in your manuscript?	Yes
**Resources**<br><br>A description of all resources used, including antibodies, cell lines, animals and software tools, with enough information to allow them to be uniquely identified, should be included in the Methods section. Authors are strongly encouraged to cite Research Resource Identifiers (RRIDs) for antibodies, model organisms and tools, where possible.<br><br>Have you included the information requested as detailed in our Minimum	Yes

Standards Reporting Checklist?	
**Availability of data and materials**<br><br>All datasets and code on which the conclusions of the paper rely must be either included in your submission or deposited in publicly available repositories (where available and ethically appropriate), referencing such data using a unique identifier in the references and in the “Availability of Data and Materials” section of your manuscript.<br><br>Have you have met the above requirement as detailed in our Minimum Standards Reporting Checklist?	Yes

Manuscript
Click here to access/download;Manuscript;ventricosusForProduction.docx

The genome of the venomous snail *Lautoconus ventricosus* sheds light on the origin of conotoxin diversity

José Ramón Pardos-Blas[1] https://orcid.org/0000-0001-7139-3153, Iker Irisarri[1,2,3] https://orcid.org/0000-0002-3628-1137, Samuel Abalde[1,4] https://orcid.org/0000-0001-7790-0603 , Carlos M. L. Afonso[5] https://orcid.org/0000-0002-7827-7806, Manuel J. Tenorio[6] https://orcid.org/0000-0003-4088-4958, and Rafael Zardoya[1]* https://orcid.org/0000-0001-6212-9502

[1]Departamento de Biodiversidad y Biología Evolutiva, Museo Nacional de Ciencias Naturales (MNCN-CSIC), José Gutiérrez Abascal 2, 28006, Madrid, Spain;

[2]Department of Applied Bioinformatics, Institute for Microbiology and Genetics, University of Goettingen, Goldschmidtstr. 1, D-37077, Goettingen, Germany;

[3]Campus Institute Data Science (CIDAS), Goettingen, Germany;

[4]Department of Zoology, Swedish Museum of Natural History, Frescativägen 40, 114 18 Stockholm, Sweden;

[5]Centre of Marine Sciences (CCMAR), Universidade do Algarve, Campus de Gambelas, 8005-139 Faro, Portugal;

[6]Departamento CMIM y Q. Inorgánica-INBIO, Facultad de Ciencias, Universidad de Cadiz; 11510 Puerto Real, Cádiz, Spain.

*Corresponding author: rafaz@mncn.csic.es

Abstract

Background: Venoms are deadly weapons to subdue prey or deter predators that have evolved independently in many animal lineages. The genomes of venomous animals are essential to understand the evolutionary mechanisms involved in the origin and diversification of venoms. **Results**: Here, we report the chromosome-level genome of the venomous Mediterranean cone snail, *Lautoconus ventricosus* (Caenogastropoda: Conidae). The total size of the assembly is 3.59 Gb; it has high contiguity (N50= 93.53 Mb) and 86.6 of the genome assembled into the 35 largest scaffolds or pseudochromosomes. Based on venom gland transcriptomes, we annotated 262 complete genes encoding conotoxin precursors, hormones, and other venom-related proteins. These genes were scattered in the different pseudochromosomes and located within repetitive regions. The genes encoding conotoxin precursors were normally structured into three exons, which did not necessarily coincide with the three structural domains of the corresponding proteins. Additionally, we found evidence in the *L. ventricosus* genome for a past whole genome duplication event by means of conserved gene synteny with the *Pomacea canaliculata* genome, the only one available at the chromosome level within Caenogastropoda. The whole genome duplication event was further confirmed by the presence of a duplicated *hox* gene cluster. Key genes for gastropod biology including those encoding proteins related with development, shell formation, and sex were located in the genome. **Conclusions**: The new high-quality *L. ventricosus* genome should become a reference for assembling and analyzing new gastropod genomes and will contribute to future evolutionary genomic studies among venomous animals.

Key words: Mediterranean cone snail; *Lautoconus ventricosus*; chromosome-level genome; venom gland transcriptome; conotoxin precursor genes; whole genome duplication

Background

The use of venoms is one of the most sophisticated ways found in nature to efficiently subdue preys or deter predators [1, 2]. Even though the production of venoms is energetically expensive, these deadly bioactive compounds confer a selective advantage, and thus their use has evolved recurrently in many distinct animal lineages such as jellyfish, centipedes, wasps, scorpions, spiders, cone snails, stonefish, and snakes [3, 4]. The snakes are undoubtedly the most dangerous to humans, and are widely accepted as the main model system in venom research, having pioneered the application of methodological advances [5] and dominated the postulation of hypotheses in the field [6-8].

Each venomous animal lineage represents an independent evolutionary experiment in which selective pressures have arrived at a unique combination of versatile venoms, whose compositions are dynamically adjusted at the genetic, transcriptional and protein levels [4]. The comparison of these venomous animal lineages at the different levels within a phylogenetic framework should provide evolutionary insights on how the diversity of venoms is originated and maintained as well as contribute to therapeutic advances [2]. In this regard, the powerful combination of high-throughput proteomics and transcriptomics is allowing the systematic cataloguing of the venom arsenals of numerous animal species beyond snakes (e.g., [9, 10]), including some previously neglected taxa [11]. These valuable data need to be complemented with genomic data to ensure gene completeness and homology prediction [12]. Moreover, identifying the ongoing evolutionary processes governing the genetic control of venom variation ultimately requires the sequencing of the genomes of various venomous animals to find common patterns and gain knowledge on how toxin-encoding genes are distributed within the different genomes, their exact copy number, exon/intron structure, conserved synteny to other genes, regulatory regions, or potential association to repetitive

elements. However, the advance of comparative genomics of venomous animals still awaits the necessary impetus. Although several genomes of venomous animals are available, the great majority was generated with short-read technology, which resulted in fragmented assemblies not amenable to answer most of the above-mentioned questions [13-15]. One notable exception is the comparative analysis of the Hispaniolan solenodon genome that demonstrated the convergent origin of venoms in eulipotyphlan mammals [16]. Recently, the chromosome-level genome assembly of the Indian cobra *Naja naja* was reported [17]. The contiguity of this genome allowed determining the organization and localization of a set of 139 toxin-encoding genes classified into 33 gene families [17]. Genomes of two jellyfish have been also recently assembled at the chromosomal level [18], although not used to study venom evolution.

With >900 species, cone snails are a highly diverse natural group living preferentially in the intertidal zone of tropical and subtropical regions worldwide [19]. They are key marine predators that produce venom to prey on worms, snails, and fish, as well as to defend against predators [20]. The venom is a cocktail composed of hundreds of peptides named conotoxins, which are synthesized as precursors with a three-domain structure: a conserved signal region (used to classify precursors into "superfamilies" [21]; a pro-peptide region involved in the processing of the precursor [22]; and a highly variable, cysteine-rich mature region, which is the functional toxin [23]. It has been proposed that the striking hyperdiversity of conotoxins has been generated through the combination of different mechanisms, including gene duplication, accelerated substitution rates, recombination, alternative splicing, differential expression, and post-translational modifications [24-28].

Here, we report on the *de novo* chromosome-level genome and transcriptome assemblies of the Mediterranean cone snail *Lautoconus ventricosus* (Gmelin, 1791), a vermivorous

species that inhabits the Mediterranean Sea and nearby Atlantic coast. Previous attempts to sequence and assemble the genome of a cone snail using short-read technology were largely unsuccessful [14, 29, 30]. The high contiguity of the newly assembled genome (together with the comprehensive catalogue of transcripts encoding conotoxin precursors derived from the venom gland transcriptome) allowed us to determine the organization of the conotoxin genes in the genome and to shed light on the genomic basis of conotoxin diversity. Moreover, because few chromosome-level genomes are available for gastropods, the cone snail genome will be particularly useful for wider evolutionary genomic studies in mollusks. In this regard, we compared the *L. ventricosus* genome to that of the ampullariid *Pomacea canaliculata* [31], the only other caenogastropod genome assembled at the chromosomal level. This comparison revealed in the *L. ventricosus* genome, the presence of a past whole genome duplication (WGD), which was previously hypothesized using chromosomal counts to have occurred in the ancestor of Neogastropoda and related families [32].

Results and discussion

De novo sequencing, assembly and annotation of the *L. ventricosus* genome

A high-quality assembly of the Mediterranean cone snail *L. ventricosus* was generated from PacBio, Chicago, and Dovetail Hi-C libraries. First, 192.6 Gb of long read sequence data (54x coverage) were produced with PacBio Sequel II and assembled *de novo* into 46,042 contigs (N50=185.88 kb; the largest contig was 1.71 Mb). Little signature of potential exogenous DNA contamination was detected (Supplementary Fig. S1). In parallel, a total of 761 and 680 Gb of short read sequence data were produced with Illumina HiSeq X from the Chicago and Hi-C libraries, respectively. Together, the Chicago library reads provided 5.25x physical

coverage of the genome (1-100 kb pairs) and the Hi-C library reads provided 381.12x physical coverage of the genome (10-10,000 kb pairs).

A second assembly round using proximity ligation information led to 19,399 scaffolds, the largest having 184.22 Mb (Supplementary Table S1). The N50 was 93.52 Mb and 86.6% of the genome was assembled into the 35 largest scaffolds or pseudochromosomes (Fig. 1 and Supplementary Fig. S2). The total size of the assembly was 3.59 Gb, which represents 87.6% of the haploid genome size estimated by flow cytometry (4.1 Gb). Together with the cephalopod *Euprymna scolopes* (5.1 Gb [33]), they are the largest mollusk genomes thus far sequenced [34]. Within gastropods, it is twice the size of that of *Achatina immaculata* (1.75 Gb [35]) and about eight times larger than most gastropod genomes including those of *P. canaliculata* (446 Mb [31]), *Chrysomallon squamiferum* (444 Mb [36]), and *Lottia gigantea* (348 MB [37]). The obtained genome size is above the estimated 3.02 Gb for *Pionoconus consors* [29], 2.76 Gb for *Kioconus tribblei* [14], and 2.56 Gb for *Textilia bullata* [30] using k-mer frequency distribution and simulations. However, it matches the 3.60 Gb of the *Darioconus pennaceus* genome and is below the 3.90 Gb of the *Lividoconus lividus* genome, which were estimated based on fluorometric assays of sperm cells [38]. With regards to the haploid number of chromosomes in Conidae, it generally varies from n=16 in *Pionoconus magus* [39] to n=35 in *Virroconus coronatus* [40]. This range in chromosome numbers is common within gastropods [41]. For *L. ventricosus* (as its synonym *Conus mediterraneus*), the haploid number of chromosomes was estimated to be n=36, although few specimens had 34, 35, or 37 chromosomes [42]. Therefore, either our specimen had 35 chromosomes and the chromosome numbers vary along the Mediterranean populations or the scaffolding failed to reconstruct one chromosome.

The 35 assembled pseudochromosomes varied in size from 44 to 184 Mb (Supplementary Fig. S3). The overall G+C content of the genome was 43.78%, above the 29.74% inferred from the partial genome of *Kioconus tribblei* [14] and the 33-40% generally reported for gastropods [31]. We could not estimate the heterozygosity of the genome assembly based on the PacBio CLR sequence data due to the error rate associated to the long reads [43]. Instead, we estimated 1.05-1.08% heterozygosity from transcriptome reads, which were obtained with the Illumina technology. This heterozygosity (restricted to coding regions) is similar to that estimated for the gastropod *Haliotis rufescens* and within the range estimated for different mollusks [44]. The repeat regions were homogenously distributed in the genome (Fig. 1) and occupied 53.36% of the genome (Class I TEs, 17.69%; Class II TEs, 11.42%; Simple repeats, 10.29%), which is a high proportion compared with *P. canaliculata* (11.4% [31]) or *C. squamiferum* (25.2% [36]), but this variation could be in part due to differences in assembly and repeat annotation. A total of 32,675 protein-coding genes were predicted, adding up to 35.9 Mb (1% of the genome). This large number of protein-coding genes is above the average gene content reported for gastropods [31, 34-37] and comparable to the gene content of cephalopods [45] and sponges [46]. Strikingly, the genome of the scallop *Pecten maximus* has been estimated to contain >67,000 protein-coding genes due to extensive gene duplication events followed by little gene loss [47]. The genome assembly contained 792 single-copy (82%) and 28 duplicated (2.9%) complete genes, as well as 41 fragmented genes (4.3%) of the BUSCO Metazoan ortholog database (odb) 10 [48]. The completeness is similar to that reported for *Achatina fulica* (91.7% [34]) and lower than those of *C. squamiferum* (96.6% [36]) and *P. canaliculata* (98.9% [31]). The BUSCO metrics for the annotated gene models were much lower: complete single-copy, 31.2%; complete duplicated, 0.4%; fragmented, 21.7%. The main methodological limitation that may explain the missing loci would be the

error rate associated to the PacBio CLR sequencing technology (15% [49]), which despite partially corrected by coverage, would hamper BLAST similarity searches.

Genome distribution and structure of conotoxin precursor genes

The transcriptome of the venom gland of another *L. ventricosus* specimen was used to identify and annotate venom-related transcripts i.e., those encoding conotoxin precursors, hormones, and proteins involved in the processing of conotoxins or in enhancing venom activity. A total of 289 different transcripts were identified using BLAST searches. Of these, 245 transcripts were assigned to 54 conotoxin precursor superfamilies based on the divergence of the signal domain and the presence of different cysteine frameworks; 11 transcripts were classified into nine hormone gene families; and 33 were assigned to 11 gene families encoding proteins related to venom synthesis or function (Supplementary Table S2 and File S1). These numbers are in agreement with those typically reported for other venom gland transcriptomes of cones [9, 29, 50-53]. Most (94%) transcripts were assembled with a complete open reading frame. As in other cone venom gland transcriptomes [9, 50, 52], O1, T, M, O2, and Conkunitzin superfamilies were the most diverse (Supplementary Table S2 and File S1).

The foot transcriptomes of another two specimens were generated for genome annotation (Supplementary Table S2 and File S1). Surprisingly, foot transcriptomes also contained transcripts encoding for conotoxin precursors. There have been reports of minor conotoxin expression outside the venom gland in the salivary gland of *Puncticulis pulicarius* [54] and in the radular sac of *Gastridium geographus* [20], but this is the first report of several conotoxin precursor transcripts in the foot. The two foot transcriptomes contained respectively a total of 35 and 49 conotoxin precursor, 3 and 1 hormone, and 25 and 19 other venom-related protein

transcripts (Supplementary Table S2 and File S1). A total of 15-20% of the conotoxin precursor superfamilies detected in the venom gland were also co-expressed in the foot; 9-27% of the hormone families; and 58-73% of other venom-related protein families. Most conotoxin precursor superfamilies expressed in the foot had lower expression values about one order of magnitude than in the venom gland (Supplementary Table S3 and Fig. S4). For B2, I1, M, and Cver01 superfamilies, expression was up to two orders of magnitude lower whereas A and Q superfamilies showed similar expression levels in both tissues (Supplementary Table S3 and Fig. S4). The transcripts encoding insulin related peptides 1, 3, and 4 were exclusively expressed in the venom gland, and the latter showed the highest expression levels. The transcripts encoding insulin related peptide 2, Prohormone-4b, and the other venom-related proteins had one order of magnitude higher expression levels in the foot than in the venom gland (two orders of magnitude for conoporin; Supplementary Table S3 and Fig. S4), indicating that these hormones and proteins may be endogenous, having a physiological function common to different tissues and not restricted to the venom gland. This could be the case of ferritin, which shows high expression in the distal section of the venom gland of *Chelyconus ermineus* [50], and was highly expressed in the foot of *L. ventricosus*. This is a protein that generally regulates the storage and release of iron, and has been related to the incorporation of iron into the radula in some chitons [44] and limpets [55], and into the shell in the pearl oyster [56]. Altogether these results corroborate the specialized secretory function of the venom gland, which is expressing higher levels of conotoxin precursor and some insulin-related transcripts. At the same time, they also point to the presence of a basal ("leaky") expression of those transcripts in the foot, which is not deleterious for the animal. As in other gastropods, the foot of the cone snail produces mucus and low levels of conotoxins in the mucus could have an antimicrobial role, as has been

demonstrated for conotoxins from *Ximeniconus ximenes* [57] and *Californiconus californicus* [58]. The detection of low expression levels of toxin genes in different tissues outside the venom gland has been demonstrated in snakes [59] and the platypus [60]. To explain the evolutionary origin of this pattern, it has been suggested that toxin genes could emerge either through gene duplication and adaptive neofunctionalization of physiological genes in the venom gland coupled with reduction of expression levels in other tissues [7, 59] or alternatively by subfunctionalization through neutral evolution and restriction to the venom gland [8].

Venom-related transcripts were used to locate the corresponding genes in the pseudochromosomes of the genome (Fig. 2). First, BLASTN searches (1e-5) using the 289 transcripts of the *L. ventricosus* transcriptome as query were performed against the 35 pseudochromosomes (Supplementary Table S4, Fig. S5 and File S2). A total of 233 genes were found complete in the genome. Of these, 154 genes were located complete in the 35 pseudochromosomes and the remaining 79 genes were completed manually with hits located in smaller scaffolds, contigs, and raw reads (Supplementary Table S4, Fig. S5 and File S2). Of the 233 complete genes, 213 corresponded to transcripts of the *L. ventricosus* venom gland transcriptome (74%) and the remaining 20 genes were not expressed. The percentage of the transcriptome detected in the genome assembly is considerably lower than the expected according to general BUSCO results (89.2%). The extra 15% of transcripts of the transcriptome without a gene counterpart in the genome could be isoforms that could be produced naturally during expression or generated as artifacts during transcriptome assembly. To test whether other assemblers could improve the final transcriptome, we run TransPi [61], a program which uses various assemblers and k-mers to generate a non-redundant consensus *de novo* transcriptome. However, the resulting *L. ventricosus* venom gland transcriptome did

not significantly improve the BUSCO scores obtained using Trinity alone (not shown). It should be also noted that part of the discrepancy between number of conotoxin genes versus transcripts may be due to natural variation among individuals, which has been reported in *L. ventricosus*, at least among populations [62].

Furthermore, we searched for the presence of extra (non-expressed) venom-related genes in the genome by performing BLASTN searches using venom-related transcripts derived from the transcriptomes of closely-related cone snail species [9] as query against the 35 pseudochromosomes (Supplementary Table S4, Fig. S5 and File S2). A total of 28 genes were found complete in the 35 pseudochromosomes and one more was manually completed with an exon in one of the smaller scaffolds. These extra loci (together with the 20 non-expressed genes detected using L. ventricosus transcripts as query; see above) indicate that at least 17% of the venom-related genes found complete in the genome were not expressed in the transcriptome. This proportion of non-expressed precursors is lower than the 41% estimated in *Kioconus tribblei* [14] and the 37-76% reported for several cone species based on exon capture data [63].

A total of 134 loci in the *L. ventricosus* genome represented incomplete genes. Of these, 62 loci corresponded to genes with more than one exon and the remaining 72 were single exons. These incomplete genes could correspond to any of the transcripts of the *L. ventricosus* transcriptome not assigned previously or to non-expressed genes; and it cannot be excluded that some of the single exons could represent false exon redundancies caused by long repeats during the assembly and scaffolding of PacBio CLR long reads [64].

Although venom-related genes were located throughout the genome, their distribution did not correlate with the size of pseudochromosomes (linear regression, R^2=0.005; p = 0.67; Supplementary Fig. S6). Pseudochromosomes 5, 16, 18, 20, and 28-31 were particularly rich

in conotoxin precursor genes; pseudochromosomes 10, 11, and 22 barely had one or two; only pseudochromosomes 2, 32, and 35 lacked any of these genes at all (Fig. 2 and Supplementary Table S4). The genes were generally found in regions harboring similar Class I retrotransposons like Gypsy, Penelope or RTE elements as well as Class II DNA transposons like Tc1-Mariner (within < 100 kb upstream and downstream). Genes encoding hormones were located in pseudochromosomes 3, 4, 11, 13, 16, and 21 (Fig. 2 and Supplementary Table S4). Genes encoding other venom-related proteins were found in pseudochromosomes 1-3, 6-8. 13-16, 20, 25, 26, 28, 33, and 35 (Fig. 2 and Supplementary Table S4). A scattered distribution of venom-related genes is also found in the genome of the Indian cobra, although in this case, some of the genes have experienced several rounds of tandem gene duplication and are organized in arrays within a pseudochromosome [17]. In the cone snail genome, potential arrays of B1 superfamily genes were found in pseudochromosome 4; of conkunitzin genes in pseudochromosome 16; of O1 superfamily genes in pseudochromosomes 20 and 28; and of I2 superfamily genes in pseudochromosome 23 (Fig. 2 and Supplementary Table S4).

The majority (62.6%) of the complete conotoxin precursor genes had three exons and two introns (Supplementary Table S4). This proportion is slightly lower to the reported 70% of conotoxin precursor genes having three exons based on exon-capture data across several cone snail species [63]. The structures of conotoxin precursor genes found in the *L. ventricosus* genome and those inferred from exon capture data show that genes encoding B1 and J superfamily peptides consistently have a single exon whereas other genes such as A and conodipine normally have two exons [63] (see also [14, 65]). Other venom-related genes typically have 11 (protein disulfide isomerases), 8 (Lysozyme), 7 (conohyaluronidase), and 4 (kazal protease inhibitor, conoporin) exons (Supplementary Table S4). The boundaries of the first and second exons do not necessarily coincide with the boundaries of signal and pro-

peptide domains but the third exon generally encodes exclusively for the mature domain (Supplementary Fig. S7). This pattern is in agreement with results obtained based on exon-capture data for the mature domain [63]. The average length of introns 1 and 2 was 5,000 bp (Supplementary Fig. S8), above the 2,665 bp reported in *Kioconus tribblei* [14].

Whole genome duplication

Comparisons of homologous gene pairs between *P. canaliculata* [31] and *L. ventricosus* genomes at the chromosome level revealed a clear pattern of conserved macrosynteny in which every chromosome of *P. canaliculata* roughly corresponded to two to four chromosomes of *L. ventricosus* (Fig. 3A; Supplementary Fig. S9). This pattern supports the existence of an ancient WGD event during the evolutionary history of Caenogastropoda and explains the increase in chromosome number (14 *versus* 35) and genome size (446 Mb *versus* 3.59 Gb). In addition to the WGD, the occurrence of additional chromosomal fissions needs to be postulated. In this regard, several smaller microsyntenic regions throughout the genome were observed (Supplementary Fig. S9), suggesting a dynamic gene reorganization post-WGD. Moreover, the distribution of synonymous substitution rate (*Ks*) values between paralog pairs further supported a WGD event evidenced by the presence of a second *Ks* peak, which would correspond to the divergence between paralogs from the two ancestrally duplicated sub-genomes (Fig. 3B [66]).

The existence of a WGD event within Caenogastropoda was already predicted based on chromosome count data [32]. The WGD event was inferred to have occurred within a clade including hypsogastropodan families with an anterior inhalant siphon as morphological synapomorphy [67-69]. Specifically, the WGD event would have occurred after the divergence of families Strombidae and Calyptraeidae, in the ancestor of a lineage containing

Ranellidae, Cypraeidae, Capulidae and the Neogastropoda (which includes Conidae [32]). As new chromosome-level genomes of Hypsogastropoda are assembled and new phylogenomic studies further resolve relationships within the group, it will be possible to precisely document this ancestral WGD event and clarify whether it might be associated with the high species diversification occurred in Neogastropoda and allied families.

Hallinan and Lindberg [32] also postulated another WGD event in heterobranch gastropods. This WGD event occurred in the ancestor of Stylommatophora, an evolutionary successful group of land snails and slugs, and thus might be associated with higher species diversification and even the water-to-land transition. The comparison of macrosynteny patterns between the genomes of *P. canaliculata* and two species of *Achatina* illustrates this WGD [35]. The genomes of the *Achatina* species have 31 chromosomes and sizes of 1.75 - 2.12 Gb [34, 35]. The macrosynteny relationships of this WGD indicate completely different evolutionary outcomes compare to the WGD event within Caenogastropoda [35], highlighting the role of contingency and the complexity of selective processes upon each WGD.

Hox genes and other genes of interest for gastropod biology

A complete set of *hox* genes was located as a cluster in pseudochromosome 26 (Fig. 3C). The gene order in the *hox* cluster is similar to the one considered ancestral in gastropods and found in *L. gigantea* [37] and *C. squamiferum* [36], but includes differences affecting two regions: *hox1-hox5* and *lox5-post1* (Fig. 3C). According to the phylogeny, two equally parsimonious scenarios could render the observed pattern for the former region: 1) an inversion of *hox1-hox5* in the common ancestor of *A. immaculata*, *P. canaliculata*, and *L. ventricosus* followed by an inversion of the *hox5* gene in *L. ventricosus* and a reversal to the ancestral state in *P. canaliculata* (three steps); and 2) two independent inversions of *hox1-hox5* in *A. immaculata,*

and of *hox1-hox4* in *L. ventricosus*, respectively, the latter followed by a translocation (three steps). With regards to the *lox5-post1* region, an inversion is shared by *L. ventricosus* and *P. canaliculata*, indicating that it likely occurred in the common ancestor of Caenogastropoda.

The plesiomorphic state for *hox* expression in mollusks is represented by the staggered expression along the anterior-posterior body axis of one chiton [70]. Within Conchifera, temporal staggered expression is observed only during the early mid-stage trochophore larva of one scaphopod [71], in the embryo stage 19/20 of one cephalopod [72], and for anterior *hox* genes in the pre-torsional veliger of several gastropods [73]. This latter expression pattern is likely favored by gene co-linearity and sub-clustering of *hox1-5* genes as shown in pesudochromosome 26 of the *L. ventricosus* genome (and other gastropods [35, 37]). By contrast, in the other larval stages in cephalopods and gastropods, *hox* gene expression is not staggered along the anterior-posterior axis but occurs in distinct morphological structures [74].

A second *hox* cluster was located in pseudochromosome 33 (Fig. 3C). The presence of this second cluster further supports the presence of an ancestral WGD. It contains only five out of the 11 *hox* genes present in gastropods. Because pseudochromosome 33 contained some gap regions, we searched for the missing genes in other pseudochromosome and in contigs not incorporated into scaffolds, but without success. Hence, we suggest that the missing genes were pseudogenized and eliminated after the WGD. In fact, *Achatina* shows a similar pattern with one complete and one partial *hox* cluster (Fig. 3C [35]).

With regards to the *parahox* gene cluster, it was only found in pseudochromosome 7 and contained *gsx* and *xlox* (also named *pdx*) genes but not the *cdx* gene, which was neither located in other pseudochromosomes nor in contigs not incorporated into scaffolds (Fig. 3C). Other gastropod genomes have the complete set of three *parahox* genes [31, 35-37]. As in

Achatina, we found one *parahox* cluster, and thus the second cluster derived from both WGDs must have been secondarily lost in both species [35].

The genes involved in other important developmental pathways were also identified and located. The dorsal-ventral patterning of a protostome embryo is controlled by the dorsal expression of the decapentaplegic (*dpp*) gene and the ventral expression of the *chordin* and *noggin* genes [74]. Pseudochromosomes 4 and 11 had one copy each of the *dpp* gene (resulting from the WGD). The *chordin* gene was located in pseudochromosome 17 and the noggin gene has two inverted paralogs within 1 Mb distance in pseudochromosome 2. Left-right body asymmetry in gastropods is the result of larval torsion (rotation of the visceral mass, mantle, and shell by 180° with respect to the head and foot) and is governed by the expression first of the diaphanous-related formin (*ldia2*) gene [75] and later of *nodal* and *pitx* genes [76]. The *ldia2* gene was located in pseudochromosome 8; there is one copy of *nodal* in pseudochromosomes 1 and 2 (which result from the WGD); the *pitx* gene is in pseudochromosome 1. Stem cell proliferation, migration and differentiation into tissues are the result of activation of various signaling proteins expressed by e.g., hedgehog (*hh*) and *notch* genes [77]. One copy of the *hh* gene was located in pseudochromosome 26 and the *notch* gene was found in pseudochromosome 31. The Nitric Oxide synthase controls in gastropods the early stages in the development of shell gland, digestive gland, and kidney, as well as the induction of larval metamorphosis [78]. The gene encoding the Nitric Oxide synthase was located in pseudochromosome 25.

One of the most important features of a gastropod is the shell. Several genes including *engrailed* and *camlbp I* have been involved in the differentiation of a shell field distinct from the mantle tissue [79]. Two copies of the *engrailed* gene were located in pseudochromosome 26, and one each in pseudochromosomes 5, 7, and 33. The *camlbp I* gene was found in

pseudochromosome 3. Although a large proportion of genes involved in generating shell structure are lineage-specific [80], some genes such as that encoding laminin [80] is commonly involved in the formation and biomineralization of the shell matrix across lineages. The genes encoding laminin subunits alpha, beta, and gamma were located in pseudochromosomes 6, 10, and 24, respectively. In the adult, the shell is often brightly colored due to the presence of three types of pigments: carotenoids, tetrapyrroles, and melanins [81]. The biosynthesis of the melanin is controlled by the tyrosinase, an enzyme that catalyzes the oxidation of tyrosine into L-DOPA in the mantle [82], producing dark purple, brown and black patterns in the pigmented sell layers of the shells of several mollusks (although apparently not in *Conus marmoreus* [83]). We identified and located the gene encoding tyrosinase in pseudochromosome 18.

As in other animals, sex determination is crucial in snails, which can have separate sexes or be hermaphrodite. No sex genes have been yet identified in gastropods. However, it is well documented that female snails of many gastropod species (particularly within the family Muricidae) can undergo masculinization when exposed to tributyltin (TBT), an environmental organic contaminant [84]. This process is called imposex, and although the exact mechanism of the endocrine disruption is not fully understood, it is clearly connected with the retinoid X receptor signaling pathway [85]. The exposure to TBT produces a local increase in the transcription levels of the *rxr* gene in the penis-forming field [84]. This gene was located in pseudochromosome 15.

Besides the mentioned key genes, we studied gene family expansions and contraction patterns in *L. ventricosus*. Comparisons of orthogroups among gastropods showed that patterns of expansion and contraction were more dynamic in terminal than internal branches (Supplementary Fig. S10). This pattern is likely the product of the sparse taxon

sampling due to the few available gastropod genomes that hardly represent the vast gastropod diversity. More orthogroups expanded than contracted in all branches. Size change in the *L. ventricosus* lineage was significant for 443 orthogroups, of which 292 expanded and 151 contracted (Supplementary Table S5). A total of 231 (52%) of these orthogroups were of unknown function, although 168 rendered BLAST hits preferentially with other gastropods (Supplementary Table S5). Expanded orthogroups may represent cases of adaptation; of those with assigned function and ontology, several were related to chromatin and nucleic acid binding as well as cellular and metabolic processes; many to keratinization, calcification/ shell formation, and mucus and adhesive protein secretion; and some related to ion transport, nervous system signaling, and hemostasis (Supplementary Table S5).

Conclusions

Understanding the genetic basis of the evolutionary processes shaping the origin and diversification of venoms requires the comparison of venomous animal genomes, preferentially assembled at the chromosome-level. Here, we provide a high-quality genome of a cone snail. There are >900 species of cone snails and this genome will serve as best reference for the assembly of other genomes within this group of marine venomous snails, opening the door to comparative analyses aimed at understanding the evolutionary origin and dynamics of conotoxin precursor gene families. Likewise, this resource will back up ongoing efforts in cataloguing toxin diversity through transcriptomic and proteomic analyses of cone snail venom glands and bolster the search for new drugs. In addition, it will be useful in characterizing the genetic consequences of a WGD event in the caenogastropod lineage.

During the review process of this article, the genome assembly of the vermivorous cone snail *Dendroconus betulinus* was reported [86], showing results highly congruent with our

findings. The genome of this species was of similar size and assembled also into 35 major scaffolds. Up to 133 conotoxin precursor genes were identified and located in the different scaffolds [86]. As in the case of *L. ventricosus,* the ratio of conotoxin precursor genes and transcripts in *D. betulinus* was close to one, and thus it was inferred that the high diversity of functional conotoxins is achieved at the post-translational level [86]. The assembly of two chromosome-level genomes of cone snails opens the door to fruitful comparative genomic studies aimed at further understanding the origin and evolution of conotoxin diversity.

Methods

Sampling

Adult specimens of *L. ventricosus* (NCBI:txid117992)were sampled in Olhão, Portugal. Once in resting stage, each individual was extracted from the shell with a sewing needle and dissected to obtain foot muscle, which was flash frozen in liquid nitrogen and stored at -80ºC, as well as a piece of foot and the venom gland, which were preserved in RNAlater (Thermo Fisher Scientific, Waltham, MA, USA) and stored at -20ºC.

Flow cytometry

Flow cytometry was used to determine the haploid genome size of *L. ventricosus*. The genome of the German cockroach *Blattella germanica* (1C = 2.025 Gb, [87]) was used as reference. Briefly, cells were isolated from the head of a cockroach and from the foot and proboscis of the cone snail [88], and incubated in lysis buffer LB01 [89] with 2% of tween, propidium iodide (50 μg/mL), and RNAse (40 μg/mL). After 10 minutes, the processed tissue was filtered using a nylon mesh of 20 μm. The DNA content of the diploid cells was determined through the relative G0/G1 peak positions of the stained nuclei using a Gallios

flow cytometer (Beckman Coulter, Inc, Fullerton, CA); the results were based on the average of three individuals, counting a minimum of 5,000 cells per individual.

DNA extraction, library preparation and sequencing

DNA isolation and genome sequencing, assembly and annotation were carried out by Dovetail Genomics (Scotts Valley, CA, USA). High molecular weight (HMW) DNA was obtained from foot tissue stored at -80ºC using Genomic-tip 20G (Qiagen, Toronto, Canada) columns. DNA extractions were quantified using Qubit 2.0 Fluorometer (Life Technologies, Carlsbad, CA, USA) and their quality verified by gel electrophoresis. A total of 15 µg of HMW DNA from individual CV1492 (the shell was deposited as voucher in the MNCN collection under accession number MNCN 15.05/92196) was used to generate four PacBio SMRTbell libraries (~20kb). Sequencing was performed on four PacBio Sequel II SMRT cells. Sequencing yields were 52.2, 50.3, 45.0 and 45.1 Gb.

Three Chicago and three Hi-C libraries were prepared following [90] and [91], respectively. A total of 0.5 µg of HMW DNA from individual CV1495 (MNCN 15.05/92199) was used per library. Briefly, for Chicago libraries, HMW DNA (mean fragment length = 50 kb) was reconstituted into chromatin *in vitro* and fixed with formaldehyde. For Dovetail Hi-C libraries, chromatin was fixed in place with formaldehyde in the nucleus and then extracted. For both libraries, fixed chromatin was digested with DpnII, the 5' overhangs filled in with biotinylated nucleotides, and free blunt ends were ligated. After ligation, crosslinks were reversed and the DNA purified and treated to remove biotin that was not internal to ligated fragments. The DNA was then sheared to ~350 bp mean fragment size and sequencing libraries were generated using NEBNext Ultra enzymes and Illumina-compatible adapters. Biotin-containing fragments were isolated using streptavidin beads before PCR enrichment of

each library. All six libraries were sequenced on an Illumina HiSeq X platform (paired-end, 2x151 bp). The read pairs produced for the Chicago libraries were 322, 162, and 277 Gb, and for the Dovetail HiC libraries were 145, 426, and 109 Gb.

Genome assembly and scaffolding

Long reads sequenced in the four SMRT cells were *de novo* assembled using wtdgb2 [92]. This software computes the consensus haploid sequence of each contig and produces less false duplications than other assemblers [92], as tested in our genome assembly using Purge Haplotigs [93] and confirmed by the low number of duplicates in the BUSCO scores. The initial *de novo* assembly, shotgun long-reads, Chicago library reads, and Dovetail Hi-C library reads were used as input data for HiRiSE^{TM}, a software pipeline designed specifically for using proximity ligation data to scaffold genome assemblies [90]. An iterative analysis was conducted. First, Shotgun and Chicago library sequences were aligned to the draft input assembly using a modified SNAP read mapper [94]. The separations of Chicago read pairs mapped within draft scaffolds were analyzed by HiRiSE^{TM} to produce a likelihood model for genomic distance between read pairs, and the model was used to identify and break putative misjoins, to score prospective joins, and make joins above a threshold. After aligning and scaffolding Chicago data, Dovetail Hi-C library sequences were aligned and merged into scaffolds following the same method. After scaffolding, shotgun sequences were used to close gaps between contigs [90].

RNA extraction, library preparation and sequencing

The transcriptomes of the foot of individuals CV10 and CV19 (for wide gene annotation), as well as that of the venom gland of individual CV8 (for venom-related gene annotation) were

determined. Each foot and venom gland tissue was incubated independently in 300 µl of TRIzol LS reagent (Thermo Fisher Scientific, Waltham, MA, USA) and ground with ceramic beads in a Precellys Evolution tissue homogenizer. The solution was mixed with 60 µl of chloroform. After centrifugation (12,000 x g for 15 min at 4ºC), the aqueous phase was recovered and RNA precipitated in one volume of isopropanol and incubated overnight at -80 ºC. The Direct-zol RNA miniprep kit (Zymo Research, Irvine, CA, USA) was used to purify 5–15 µg of total RNA following manufacturer's instructions.

Library construction and sequencing of the venom gland transcriptome was conducted at AllGenetics (Oleiros, Spain) in 2012 whereas foot transcriptomes were obtained at Sistemas Genómicos (Valencia, Spain) in 2016. Briefly, dual-indexed cDNA libraries (307–345 bp insert average size) were constructed for each sample using the TruSeq RNA Library Prep Kit v2 (Illumina, San Diego). The quality and quantity of the libraries was determined with the TapeStation 4200, High Sensitivity assay, and by real-time PCR in LightCycler 480 (Roche), respectively. Libraries were split into two flowcells and sequenced in an Illumina HiSeq2000 (paired-end, 2x100bp) platform.

Transcriptome assembly

For each sample, RNA-seq raw reads were checked using FastQC (FastQC, RRID:SCR_014583) v0.10.1 [95]. Transcriptomes were assembled *de novo* using Trinity (Trinity, RRID:SCR_013048) v2.6.6 [96] with default parameters and the trimmomatic option activated. TransPi [61], which generates a consensus transcriptome assembly with different assemblers was also used following the program instructions and using k-mer of 25, 41, 53 and 75. Additionally, a reference-guided assembly of the venom gland transcriptome was performed. First, clean reads were mapped onto the final genome assembly with Hisat2 v2.2.0

[97]. Then, bam file outputs were sorted and used for a genome-guided assembly with Trinity 2.6.6 using the genome guided option, max_intron of 37,000 and all other parameters as default. Completeness of both assemblies was checked using BUSCO (BUSCO, RRID:SCR_015008) v4.0.6 [98] with the metazoa_odb10 gene set. The outputs of the two assemblies were merged and redundancy was eliminated with CD-HIT v4.5.4 [99] with default parameters to obtain the final transcriptome.

In order to estimate heterozygosity, we used GenomeScope (GenomeScope, RRID:SCR_017014) 2.0 [43]. The method is a k-mer based statistical approach and due to the error rate associated to the long reads, PacBio raw data of the genome could not be used. Alternatively, Illumina pair-ended raw reads of the transcriptomes (CV8, CV10, CV19) were used to estimate heterozygosity. First, k-mer frequencies were estimated using jellyfish [100]. A range of k-mer sizes from 17 to 71 was analyzed. The different k-mer outputs were exported into K-mer count histogram files and uploaded into the GenomeScope 2.0 server. A k-mer size of 71 was selected as the best-fit model.

Genome assembly quality evaluation

Quality assessment and general metrics of the final genome assembly were obtained with Quast (QUAST, RRID:SCR_001228) v5.0.2 [101]. An evaluation of coverage was conducted mapping subreads onto the final assembly using Minimap2 [102]. Potential sources of DNA contamination were checked with Blobtools (Blobtools, RRID:SCR_017618) v1.1 [103] using the NCBI entries of viruses, archaea, bacteria, fungi, nematodes, platyhelminthes, polychaetes and human. NCBI entries for mollusks were used for the taxonomic identification of *L. ventricosus* contigs. A BLASTN search using the published mitogenome of *L. ventricosus* [104] as query was performed to detect and discard mitochondrial DNA.

Completeness of the genome assembly was assessed with BUSCO v4.0.6 **[98]** in genome mode and using the metazoa_odb10 gene set.

Transcript relative expression in venom gland *versus* foot

RNA-Seq clean reads were mapped with Bowtie2 (Bowtie 2, RRID:SCR_016368) [105] against the curated assembled transcripts and normalized in TPM (transcripts per kilobase million) using the function rsem-calculate-expression of the RSEM v1.2.31 package included in Trinity v2.6.6 [96]. TPMs derived from foot (CV10 and CV19) transcriptomes were combined and compared to those derived from the venom gland (CV8) transcriptome.

Conotoxin precursor and other venom transcript annotation: The amino acid sequences of all conotoxin precursors and associated proteins of cone venoms available in GenBank release 236, Uniprot release 2020_02 (Uniprot Consortium 2017), and ConoServer release 02-04-2020 [23] were downloaded in February 4th, 2020 to construct a custom reference database. Redundancy in database was eliminated using CDHIT v4.5.4 with a 95% identity threshold. Transcripts encoding conotoxin precursors and associated proteins were identified by BLASTX similarity searches of the transcripts against the above reference database (E-value of 1 x 10^{-5}). TBLASTX similarity searches against the NCBI NR database and manual inspection were performed in order to discard false positives (hits not corresponding to canonical conotoxins) or assembly artifacts (in low coverage terminal positions and chimaeras). Highly truncated (>55% of the estimated total length) peptide sequences were removed to produce the final working list of conotoxin precursors and associated proteins. The three domains of the predicted conotoxin precursors (signal, propeptide, and mature) and the cysteine frameworks of the mature functional peptides were identified using the Conoprec

tool [23]. Assignment of precursors to different protein superfamilies was based on the two highest scoring full-length conotoxin precursor hits in the BLAST results as well as taking into account the percentage of sequence identity (>70%) to the highly conserved signal region.

Genome annotation

Reference libraries of repetitive sequences were generated *de novo* from the genome assembly using RepeatModeler (RepeatModeler, RRID:SCR_015027) v2.0.1 [106], RECON v1.08 [107], and RepeatScout (RepeatScout, RRID:SCR_014653) v1.0.6 [108]. The custom libraries were used to identify, quantify, and mask repeat elements with RepeatMasker (RepeatMasker, RRID:SCR_012954) 4.1.0 [109].

Gene predictions were generated using AUGUSTUS (Augustus, RRID:SCR_008417) v2.5.5 [110]. The coding sequences of the genomes of three gastropods, *Aplysia californica* (GCF_000002075.1), *Biomphalaria glabrata* (GCA_000457365.1), and *Lottia gigantea* (GCA_000327385.1); three bivalves, *Crassostrea gigas* (GCA_902806645.1), *Crassostrea virginica* (GCA_002022765.4), and *Mizuhopecten yessoensis* (GCA_002113885.2); and one cephalopod, *Octopus bimaculoides* (GCA_001194135.1) were used to train the *ab initio* model for *L. ventricosus*. Three rounds of prediction optimization were done. The same coding sequences were also used to train an independent *ab initio* model with SNAP v2006-07-28 [111]. Newly generated RNA-seq reads from *L. ventricosus* and from the foot (SRX984185), mantle (SRX984179), nervous ganglia (SRX980532), and osphradium (SRX984173) transcriptomes of *P. consors* [29] were mapped onto the genome using STAR v2.7 [112]. Resulting bam files were used to generate intron hints with bam2hints in AUGUSTUS. The AUGUSTUS and SNAP models along with intron-exon boundary hints

provided from RNA-Seq were used as input to MAKER (MAKER, RRID:SCR_005309) v3.01.01 pipeline [113] to predict for genes in the repeat-masked reference genome. To help guide the prediction process, Swiss-Prot peptide sequences from the UniProt database were downloaded and used in conjunction with the protein sequences from mollusks used for gene training as peptide evidence in the Maker pipeline. To help assess the quality of the gene prediction, AED (annotation edit distance) scores were generated for each of the predicted genes as part of the MAKER pipeline. If multiple models predicted by SNAP and AUGUSTUS overlapped, only the one with the lowest AED was retained in the final annotation set. Genes were further characterized for their putative function by performing a BLAST search of the peptide sequences against the UniProt database. tRNA were predicted using the software tRNAscan-SE (tRNAscan-SE, RRID:SCR_010835) v 2.05 [114].

Gene family manual annotation

Venom-related genes in the genome: a custom non-redundant database was constructed including the nucleotide sequences of the curated list of conotoxins, hormones and other proteins derived from the transcriptome of *L. ventricosus* (see above) plus the nucleotide sequences of additional conotoxins, hormones and related venom proteins derived from the transcriptomes of 13 closely related cone snail species from Cabo Verde and Senegal [9]. A BLASTN search against the genome assembly and a TBLASTN (e-value of 1 x 10^{-5}) search of the genome assembly against the translated conotoxin database were performed. BLAST outputs were transformed to GFF3 file format and loaded into Geneious (Geneious, RRID:SCR_010519) v2020.1.2 [115]. Each hit was manually curated by adjusting intron-exon GT/AG junctions and by comparing exons with the original transcripts to detect any

broken ORF and possible missing exons. Venom-related gene annotations are reported in a separate GFF3 file (Supplementary file S3).

***Hox* and *parahox* genes**: mollusk *hox* proteins available in NCBI and the HMM profile for the homeodomain (PFAM: PF00046) were fed into BITACORA v1.2.1 [116] in order to identify members of the *hox* gene family previously not detected in the automated annotation. In addition, the genome was searched using TBLASTN and all available mollusk *hox* and *parahox* proteins in order to identify any missing homolog. The identity of *hox* and *parahox* genes was confirmed upon multiple sequence alignment MAFFT einsi (MAFFT, RRID:SCR_011811) [117]) and maximum likelihood inference under the BIC-selected best-fit model in IQTREE v1.6.12 [118]. *Hox* and *parahox* gene annotations are reported in a separate GFF3 file (Supplementary file S4).

Other genes: identification and location of genes involved in development, shell formation, color, and sex was conducted through TBLASTN searches (e-value of 1×10^{-5}) of representative NCBI entries (mostly gastropod orthologues) of each gene against the 35 pseudochromosomes. Hits were converted to GFF3 files and loaded into Geneious v2020.1.2 [115] to manually reconstruct the exon-intron boundaries.

Synteny and whole genome duplication

Conserved synteny between *L. ventricosus* and *P. canaliculata* pseudochromosomes was inferred using pairs of 1:1 and 1:2 orthologs obtained with Orthofinder (OrthoFinder, RRID:SCR_017118) v2.3.11 [119]. Synteny plots were generated with the shinyCircos package [120]. To simplify plotting, short links < 1Kb were filtered out and adjacent links (within 10 Mb) were merged using the bundlelinks tool [120]. The presence of WGD was

also assessed using WGDdetector [121], which measures the synonymous rates of substitution (*Ks*) between pairs of paralogs. The *Ks* method assumes a L-shaped distribution of *Ks* for diploid species and additional peaks in *Ks* correspond to pairs of paralogs with similar synonymous divergences expected under a shared origin time by WGD. The *Ks* distances were plotted with the R package ggplot2 [122].

Patterns of gene family evolution.

We used CAFÉ (Computational Analysis of gene Family Evolution, RRID:SCR_018924) v5.0 [123] to infer expansion and contraction of gene families in the *L. ventricosus* genome. Orthogroups were inferred with Orthofinder v2.3.11 [119] using all annotated proteins from six gastropod genomes (*Aplysia californica*, *Biomphalaria glabrata*, *Elysia chlorotica, Lautoconus ventricosus*, *Lottia gigantea*, and *Pomacea canaliculata*). A dated tree was built from the current consensus on gastropod phylogeny [124] and median divergence times from the timetree.org database [125]. Gene family expansion and contraction patterns were inferred for 11,990 orthogroups that were present at the tree root, assuming a global rate for gene family size change (lambda) and a uniform gene family size distribution at the tree root. Those orthogroups containing *L. ventricosus* genes annotated as related to transposable elements were discarded from further study. The remaining orthogroups were functionally characterized using the automated genome annotation as well as by a similarity search of their sequences against the NCBI NR database using diamond v0.9.9 [126] with an e-value threshold of 1e-6.

Availability of supporting data

Final assembly, original PacBio assembly, as well as annotation files, predicted transcript and protein sequences, and bioinformatics supporting information were deposited in the GigaScience database GigaDB [127]. Additionally, assembly, PacBio subreads and transcriptome raw data were deposited at NCBI under the bioproject number PRJNA678883. Final assembly (JAFLJL000000000); PacBio subreads (SRR13994261-SRR13994264); RNAseq raw reads (CV8: SRR13740844, CV10: SRR13757741, CV19: SRR13770976)

Additional files

Supplementary Files S1-S3, Tables S1−S5 and Figs. S1− S10 are available as additional files.

Competing interests

The authors declare no competing interests.

Author contributions

RZ conceived the study and designed the experiments and analyses. MJT and CMLA obtained the individuals, performed sample dissections, and provided information on cone snail biology. JRPB and SA worked on venom gland and foot comparative transcriptomics. JRPB and II performed genome analyses and manual gene annotations. RZ wrote the manuscript initial draft and all authors read, revise, and approved the manuscript final version.

Acknowledgements

We are grateful to Joel Vizueta, Alejandro Sánchez, and Julio Rozas for advice with genome assembly analysis and providing access to Hercules computer cluster. We thank Paula Escuer for flow cytometry analyses. We thank Shaune Hall and other staff members at Dovetail

Genomics for their assistance. We are indebted to David Osca, who participated in field sampling and cone snail dissections and to Lara de la Cita for the illustrated picture of the internal structure of a cone. This work was funded by the Spanish Ministry of Science and Innovation (CGL2016-75255-C2-1-P [AEI/FEDER, UE] and PID2019-103947GB-C22/AEI/10.13039/501100011033 to R.Z.; BES-2017-081195 to J.R.P.-B.; BES-2014-069575 to S.A.; IJCI-2016-29566 to II). II acknowledges the support from the European Research Council during the latest stages of the project (Grant Agreement No. 852725; ERC-StG 'TerreStriAL' to Jan de Vries, University of Goettingen).

References

1. Dutertre S, Modica MV, Holford M, Sunagar K. Diversity and evolution of animal venoms: neglected targets, ecological interactions, future perspectives. Laussane: Frontiers Media SA; 2020.
2. Holford M, Daly M, King GF, Norton RS. Venoms to the rescue. Science. **2018**;361:842.
3. Arbuckle K. Evolutionary context of venom in animals. In: Malhotra A, editor. Evolution of venomous animals and their toxins. Dordrecht: Springer Netherlands; 2017. p. 3-31.
4. Casewell NR, Wüster W, Vonk FJ, Harrison RA, Fry BG. Complex cocktails: the evolutionary novelty of venoms. Trends Ecol Evol. **2013**;28:219-29.
5. Post Y, Puschhof J, Beumer J, Kerkkamp HM, de Bakker MAG, Slagboom J, et al. Snake venom gland organoids. Cell. **2020**;180:233-47.e21.

6. Fry BG, Roelants K, Champagne DE, Scheib H, Tyndall JDA, King GF, et al. The toxicogenomic multiverse: convergent recruitment of proteins into animal venoms. Annu Rev Genomics Hum. **2009**;10:483-511.
7. Reyes-Velasco J, Card DC, Andrew AL, Shaney KJ, Adams RH, Schield DR, et al. Expression of venom gene homologs in diverse python tissues suggests a new model for the evolution of snake venom. Mol Biol Evol. **2014**;32:173-83.
8. Hargreaves AD, Swain MT, Hegarty MJ, Logan DW, Mulley JF. Restriction and recruitment—gene duplication and the origin and evolution of snake venom toxins. Genome Biol Evol. **2014**;6:2088-95.
9. Abalde S, Tenorio MJ, Afonso CML, Zardoya R. Comparative transcriptomics of the venoms of continental and insular radiations of West African cones. Proc R Soc Biol Sci Ser B. **2020**;287:20200794.
10. Koua D, Mary R, Ebou A, Barrachina C, El Koulali K, Cazals G, et al. Proteotranscriptomic insights into the venom composition of the wolf spider *Lycosa tarantula*. Toxins. **2020**;12:501.
11. von Reumont BM. Studying smaller and neglected organisms in modern evolutionary venomics implementing RNASeq (transcriptomics)-a critical guide. Toxins. **2018**;10:292.
12. Drukewitz SH, von Reumont BM. The significance of comparative genomics in modern evolutionary venomics. Front Ecol Evol. **2019**;7:163.
13. Schwager EE, Sharma PP, Clarke T, Leite DJ, Wierschin T, Pechmann M, et al. The house spider genome reveals an ancient whole-genome duplication during arachnid evolution. BMC Biol. **2017**;15:62.

14. Barghi N, Concepcion GP, Olivera BM, Lluisma AO. Structural features of conopeptide genes inferred from partial sequences of the *Conus tribblei* genome. Mol Genet Genom. **2016**;291:411-22.

15. Cao Z, Yu Y, Wu Y, Hao P, Di Z, He Y, et al. The genome of *Mesobuthus martensii* reveals a unique adaptation model of arthropods. Nat Commun. **2013**;4:2602.

16. Casewell NR, Petras D, Card DC, Suranse V, Mychajliw AM, Richards D, et al. Solenodon genome reveals convergent evolution of venom in eulipotyphlan mammals. Proc Natl Acad Sci USA. **2019**;116:25745.

17. Suryamohan K, Krishnankutty SP, Guillory J, Jevit M, Schröder MS, Wu M, et al. The Indian cobra reference genome and transcriptome enables comprehensive identification of venom toxins. Nat Genet. **2020**;52:106-17.

18. Nong W, Cao J, Li Y, Qu Z, Sun J, Swale T, et al. Jellyfish genomes reveal distinct homeobox gene clusters and conservation of small RNA processing. Nat Commun. **2020**;11:3051.

19. Tucker JK, Tenorio MJ. Illustrated catalog of the living cone shells. MdM Publishing; 2013.

20. Dutertre S, Jin A-H, Vetter I, Hamilton B, Sunagar K, Lavergne V, et al. Evolution of separate predation- and defence-evoked venoms in carnivorous cone snails. Nat Commun. **2014**;5:3521.

21. Robinson SD, Norton RS. Conotoxin gene superfamilies. Mar Drugs. **2014**;12:6058-101.

22. Buczek O, Bulaj G, Olivera BM. Conotoxins and the posttranslational modification of secreted gene products. Cell Mol Life Sci. **2005**;62:3067-79.

23. Kaas Q, Westermann JC, Craik DJ. Conopeptide characterization and classifications: an analysis using ConoServer. Toxicon. **2010**;55:1491-509.

24. Dutertre S, Jin AH, Kaas Q, Jones A, Alewood PF, Lewis RJ. Deep venomics reveals the mechanism for expanded peptide diversity in cone snail venom. Mol Cell Proteom. **2013**;12:312-29.

25. Chang D, Duda TF. Extensive and continuous duplication facilitates rapid evolution and diversification of gene families. Mol Biol Evol. **2012**;29:2019-29.

26. Conticello SG, Gilad Y, Avidan N, Ben-Asher E, Levy Z, Fainzilber M. Mechanisms for evolving hypervariability: the case of conopeptides. Mol Biol Evol. **2001**;18:120-31.

27. Lu A, Yang L, Xu S, Wang C. Various conotoxin diversifications revealed by a venomic study of *Conus flavidus*. Mol Cell Proteom. **2014**;13:105-18.

28. Wu Y, Wang L, Zhou M, You Y, Zhu X, Qiang Y, et al. Molecular evolution and diversity of *Conus* peptide toxins, as revealed by gene structure and intron sequence analyses. PLoS ONE. **2013**;8:e82495.

29. Andreson R, Roosaare M, Kaplinski L, Laht S, Kõressaar T, Lepamets M, et al. Gene content of the fish-hunting cone snail *Conus consors*. bioRxiv. **2019**;

30. Hu H, Bandyopadhyay PK, Olivera BM, Yandell M. Characterization of the *Conus bullatus* genome and its venom-duct transcriptome. BMC Genomics. **2011**;12:60.

31. Liu C, Zhang Y, Ren Y, Wang H, Li S, Jiang F, et al. The genome of the golden apple snail *Pomacea canaliculata* provides insight into stress tolerance and invasive adaptation. GigaScience. **2018**;7

32. Hallinan NM, Lindberg DR. Comparative analysis of chromosome counts infers three paleopolyploidies in the Mollusca. Genome Biol Evol. **2011**;3:1150-63.

33. Belcaid M, Casaburi G, McAnulty SJ, Schmidbaur H, Suria AM, Moriano-Gutierrez S, et al. Symbiotic organs shaped by distinct modes of genome evolution in cephalopods. Proc Natl Acad Sci USA. **2019**;116:3030.

34. Guo Y, Zhang Y, Liu Q, Huang Y, Mao G, Yue Z, et al. A chromosomal-level genome assembly for the giant African snail *Achatina fulica*. GigaScience. **2019**;8

35. Liu C, Ren Y, Li Z, Hu Q, Yin L, Wang H, et al. Giant African snail genomes provide insights into molluscan whole-genome duplication and aquatic–terrestrial transition. Mol Ecol Res. **2020**;21

36. Sun J, Chen C, Miyamoto N, Li R, Sigwart JD, Xu T, et al. The Scaly-foot snail genome and implications for the origins of biomineralised armour. Nat Commun. **2020**;11:1657.

37. Simakov O, Marletaz F, Cho S-J, Edsinger-Gonzales E, Havlak P, Hellsten U, et al. Insights into bilaterian evolution from three spiralian genomes. Nature. **2013**;493:526-31.

38. Hinegardner R. Cellular DNA content of the Mollusca. Comp Biochem Physiol A. **1974**;47:447-60.

39. Dalet JT, Saloma CP, Olivera BM, Heralde FM. Karyological analysis and FISH physical mapping of 18S rDNA genes, (GATA)n centromeric and (TTAGGG)n telomeric sequences in *Conus magus* Linnaeus, 1758. J Molluscan Stud. **2015**;81:274-89.

40. Ebied AM, Hassan HA, Abu-Almaaty AH, Yaseen AE. Cytogenetic studies on metaphase chromosomes of eight gastropod species of orders Mesogastropoda and Neogastropoda from the Red Sea (Prosobranchia-Mollusca). J Egypt Ger Soc Zool. **2000**;33:317–36.

41. Thiriot-Quiévreux C. Advances in chromosomal studies of gastropod molluscs. J Molluscan Stud. **2003**;69:187-202.

42. Vitturi R, Catalano E. Spermatocyte chromosomes in 7 species of the sub-class Prosobranchia (Mollusca, Gasteropoda). Biol Zentbl. **1984**;103:69–76.

43. Vurture GW, Sedlazeck FJ, Nattestad M, Underwood CJ, Fang H, Gurtowski J, et al. GenomeScope: fast reference-free genome profiling from short reads. Bioinformatics (Oxford, England). **2017**;33:2202-4.

44. Varney RM, Speiser DI, McDougall C, Degnan BM, Kocot KM. The Iron-responsive genome of the chiton *Acanthopleura granulata*. Genome Biol Evol. **2021**;13

45. Albertin CB, Simakov O, Mitros T, Wang ZY, Pungor JR, Edsinger-Gonzales E, et al. The octopus genome and the evolution of cephalopod neural and morphological novelties. Nature. **2015**;524:220-4.

46. Kenny NJ, Francis WR, Rivera-Vicéns RE, Juravel K, de Mendoza A, Díez-Vives C, et al. Tracing animal genomic evolution with the chromosomal-level assembly of the freshwater sponge *Ephydatia muelleri*. Nat Commun. **2020**;11:3676.

47. Kenny NJ, McCarthy SA, Dudchenko O, James K, Betteridge E, Corton C, et al. The gene-rich genome of the scallop *Pecten maximus*. GigaScience. **2020**;9

48. Waterhouse RM, Seppey M, Simão FA, Manni M, Ioannidis P, Klioutchnikov G, et al. BUSCO applications from quality assessments to gene prediction and phylogenomics. Mol Biol Evol. **2018**;35:543-8.

49. Laehnemann D, Borkhardt A, McHardy AC. Denoising DNA deep sequencing data—high-throughput sequencing errors and their correction. Brief Bioinform. **2016**;17:154-79.

50. Abalde S, Tenorio MJ, Afonso CML, Zardoya R. Conotoxin diversity in *Chelyconus ermineus* (Born, 1778) and the convergent origin of piscivory in the Atlantic and Indo-Pacific cones. Genome Biol Evol. **2018**;10:2643-62.

51. Li Q, Barghi N, Lu A, Fedosov AE, Bandyopadhyay PK, Lluisma AO, et al. Divergence of the venom exogene repertoire in two sister species of *Turriconus*. Genome Biol Evol. **2017**;9:2211-25.

52. Pardos-Blas JR, Irisarri I, Abalde S, Tenorio MJ, Zardoya R. Conotoxin diversity in the venom gland transcriptome of the Magician's cone, *Pionoconus magus*. Mar Drugs. **2019**;17:553.

53. Peng C, Yao G, Gao B-M, Fan C-X, Bian C, Wang J, et al. High-throughput identification of novel conotoxins from the Chinese tubular cone snail (*Conus betulinus*) by multi-transcriptome sequencing. GigaScience. **2016**;5:17.

54. Biggs JS, Olivera BM, Kantor YI. Alpha-conopeptides specifically expressed in the salivary gland of *Conus pulicarius*. Toxicon. **2008**;52:101-5.

55. Shaw JA, Macey DJ, Brooker LR. Radula synthesis by three species of iron mineralizing molluscs: production rate and elemental demand. J Mar Biol Assoc U K. **2008**;88:597-601.

56. Zhang Y, Meng Q, Jiang T, Wang H, Xie L, Zhang R. A novel ferritin subunit involved in shell formation from the pearl oyster (Pinctada fucata). Comp Biochem Physiol B Comp Biochem. **2003**;135:43-54.

57. Figueroa-Montiel A, Bernáldez J, Jiménez S, Ueberhide B, González LJ, Licea-Navarro A. Antimycobacterial activity: a new pharmacological target for conotoxins found in the first reported conotoxin from *Conasprella ximenes*. Toxins. **2018**;10

58. Bernáldez-Sarabia J, Figueroa-Montiel A, Dueñas S, Cervantes-Luévano K, Beltrán JA, Ortiz E, et al. The diversified O-superfamily in *Californiconus californicus* presents a conotoxin with antimycobacterial activity. Toxins. **2019**;11

59. Junqueira-de-Azevedo ILM, Bastos CMV, Ho PL, Luna MS, Yamanouye N, Casewell NR. Venom-related transcripts from *Bothrops jararaca* tissues provide novel molecular insights into the production and evolution of snake venom. Mol Biol Evol. **2015**;32:754-66.

60. Whittington CM, Belov K. Platypus venom genes expressed in non-venom tissues. Aust J Zool. **2009**;57:199-202.

61. Rivera-Vicéns RE, Garcia Escudero C, Conci N, Eitel M, Wörheide G. TransPi – a comprehensive traanscriptome analysis pipeline for *de novo* transcriptome assembly. bioRxiv. **2021**:2021.02.18.431773.

62. Romeo C, Di Francesco L, Oliverio M, Palazzo P, Massilia GR, Ascenzi P, et al. Conus ventricosus venom peptides profiling by HPLC-MS: A new insight in the intraspecific variation. Journal of Separation Science. **2008**;31:488-98.

63. Phuong MA, Mahardika GN. Targeted sequencing of venom genes from cone snail genomes improves understanding of conotoxin molecular evolution. Mol Biol Evol. **2018**;35:1210-24.

64. Lang D, Zhang S, Ren P, Liang F, Sun Z, Meng G, et al. Comparison of the two up-to-date sequencing technologies for genome assembly: HiFi reads of Pacific Biosciences Sequel II system and ultralong reads of Oxford Nanopore. GigaScience. **2020**;9

65. Yuan D-D, Han Y-H, Wang C-G, Chi C-W. From the identification of gene organization of α conotoxins to the cloning of novel toxins. Toxicon. **2007**;49:1135-49.

66. Maere S, De Bodt S, Raes J, Casneuf T, Van Montagu M, Kuiper M, et al. Modeling gene and genome duplications in eukaryotes. Proc Natl Acad Sci USA. **2005**;102:5454.

67. Ponder WF, Colgan DJ, Healy JM, Nützel A, Simone LRL, Strong EE. Caenogastropoda. In: Ponder WF and Lindberg DL, editors. Molluscan phylogeny and evolution. Berkeley: University of California Press; 2008. p. 331-83.

68. Osca D, Templado J, Zardoya R. Caenogastropoda mitogenomics. Mol Phylogenet Evol. **2015**;93:118-28.

69. Cunha TJ, Giribet G. A congruent topology for deep gastropod relationships. Proc R Soc Biol Sci Ser B. **2019**;286:20182776.

70. Fritsch M, Wollesen T, de Oliveira AL, Wanninger A. Unexpected co-linearity of *hox* gene expression in an aculiferan mollusk. BMC Evol Biol. **2015**;15:151.

71. Wollesen T, Rodríguez Monje SV, Luiz de Oliveira A, Wanninger A. Staggered *hox* expression is more widespread among molluscs than previously appreciated. Proceedings of the Royal Society B: Biological Sciences. **2018**;285:20181513.

72. Lee PN, Callaerts P, de Couet HG, Martindale MQ. Cephalopod *hox* genes and the origin of morphological novelties. Nature. **2003**;424:1061-5.

73. Samadi L, Steiner G. Expression of *hox* genes during the larval development of the snail, *Gibbula varia* (L.)—further evidence of non-colinearity in molluscs. Dev Genes Evol. **2010**;220:161-72.

74. Wanninger A, Wollesen T. The evolution of molluscs. Biol Rev Camb Philos Soc. **2019**;94:102-15.

75. Davison A, McDowell Gary S, Holden Jennifer M, Johnson Harriet F, Koutsovoulos Georgios D, Liu MM, et al. Formin is associated with left-right asymmetry in the pond snail and the frog. Curr Biol. **2016**;26:654-60.

76. Grande C, Patel NH. *Nodal* signalling is involved in left–right asymmetry in snails. Nature. **2009**;457:1007-11.

77. De Oliveira AL, Wollesen T, Kristof A, Scherholz M, Redl E, Todt C, et al. Comparative transcriptomics enlarges the toolkit of known developmental genes in mollusks. BMC Genomics. **2016**;17:905.

78. Truchado-Garcia M, Caccavale F, Grande C, D'Aniello S. Expression pattern of Nitric Oxide Synthase during development of the marine gastropod mollusc, *Crepidula fornicata*. Genes. **2021**;12:314.

79. Jackson DJ, Wörheide G, Degnan BM. Dynamic expression of ancient and novel molluscan shell genes during ecological transitions. BMC Evol Biol. **2007**;7:160.

80. Aguilera F, McDougall C, Degnan BM. Co-option and *de novo* gene evolution underlie molluscan shell diversity. Mol Biol Evol. **2017**;34:779-92.

81. Williams ST. Molluscan shell colour. Biol Rev. **2017**;92:1039-58.

82. Nagai K, Yano M, Morimoto K, Miyamoto H. Tyrosinase localization in mollusc shells. Comp Biochem Physiol B Biochem Mol Biol. **2007**;146:207-14.

83. Affenzeller S, Wolkenstein K, Frauendorf H, Jackson DJ. Eumelanin and pheomelanin pigmentation in mollusc shells may be less common than expected: insights from mass spectrometry. Front Zool. **2019**;16:47.

84. Abidli S, Castro LFC, Lahbib Y, Reis-Henriques MA, Trigui El Menif N, Santos MM. Imposex development in Hexaplex trunculus (Gastropoda: Caenogastropoda) involves

changes in the transcription levels of the retinoid X receptor (RXR). Chemosphere. **2013**;93:1161-7.

85. Castro LFC, Lima D, Machado A, Melo C, Hiromori Y, Nishikawa J, et al. Imposex induction is mediated through the Retinoid X Receptor signalling pathway in the neogastropod *Nucella lapillus*. Aquat Toxicol. **2007**;85:57-66.

86. Peng C, Huang Y, Bian C, Li J, Liu J, Zhang K, et al. The first Conus genome assembly reveals a primary genetic central dogma of conopeptides in C. betulinus. Cell Discovery. **2021**;7:11.

87. Sánchez-Herrero JF, Frías-López C, Escuer P, Hinojosa-Alvarez S, Arnedo MA, Sánchez-Gracia A, et al. The draft genome sequence of the spider Dysdera silvatica (Araneae, Dysderidae): A valuable resource for functional and evolutionary genomic studies in chelicerates. GigaScience. **2019**;8

88. Hare EE, Johnston JS. Genome Size Determination Using Flow Cytometry of Propidium Iodide-Stained Nuclei. In: Orgogozo V and Rockman MV, editors. Molecular Methods for Evolutionary Genetics. Totowa, NJ: Humana Press; 2011. p. 3-12.

89. Dpooležel J, Binarová P, Lcretti S. Analysis of Nuclear DNA content in plant cells by Flow cytometry. Biologia Plantarum. **1989**;31:113-20.

90. Putnam NH, O'Connell BL, Stites JC, Rice BJ, Blanchette M, Calef R, et al. Chromosome-scale shotgun assembly using an *in vitro* method for long-range linkage. Genome Res. **2016**;26:342-50.

91. Lieberman-Aiden E, van Berkum NL, Williams L, Imakaev M, Ragoczy T, Telling A, et al. Comprehensive mapping of long-range interactions reveals folding principles of the Human genome. Science. **2009**;326:289.

92. Ruan J, Li H. Fast and accurate long-read assembly with wtdbg2. Nat Methods. **2020**;17:155-8.

93. Roach MJ, Schmidt SA, Borneman AR. Purge Haplotigs: allelic contig reassignment for third-gen diploid genome assemblies. BMC Bioinformatics. **2018**;19:460.

94. Zaharia M, Bolosky WJ, Curtis K, Fox A, Patterson D, Shenker S, et al. Faster and More Accurate Sequence Alignment with SNAP. arXiv. **2011**;1111.5572v1

95. Andrews S. FastQC. available at http://wwwbioinformaticsbabrahamacuk/projects/fastqc/. **2010**;

96. Grabherr MG, Haas BJ, Yassour M, Levin JZ, Thompson DA, Amit I, et al. Full-length transcriptome assembly from RNA-Seq data without a reference genome. Nat Biotechnol. **2011**;29:644-52.

97. Kim D, Paggi JM, Park C, Bennett C, Salzberg SL. Graph-based genome alignment and genotyping with HISAT2 and HISAT-genotype. Nat Biotechnol. **2019**;37:907-15.

98. Seppey M, Manni M, Zdobnov EM. BUSCO: assessing genome assembly and annotation completeness. In: Kollmar M, editor. Gene prediction: methods and protocols. New York, NY: Springer New York; 2019. p. 227-45.

99. Li W, Godzik A. Cd-hit: a fast program for clustering and comparing large sets of protein or nucleotide sequences. Bioinformatics. **2006**;22:1658-9.

100. Marçais G, Kingsford C. A fast, lock-free approach for efficient parallel counting of occurrences of k-mers. Bioinformatics. **2011**;27:764-70.

101. Gurevich A, Saveliev V, Vyahhi N, Tesler G. QUAST: quality assessment tool for genome assemblies. Bioinformatics. **2013**;29:1072-5.

102. Li H. Minimap2: pairwise alignment for nucleotide sequences. Bioinformatics. **2018**;34:3094-100.

103. Laetsch DR, Blaxter ML. BlobTools: Interrogation of genome assemblies. F1000Res. **2017**;6:1287.

104. Uribe JE, Puillandre N, Zardoya R. Beyond *Conus*: phylogenetic relationships of Conidae based on complete mitochondrial genomes. Mol Phylogenet Evol. **2017**;107:142-51.

105. Langmead B, Salzberg SL. Fast gapped-read alignment with Bowtie 2. Nat Methods. **2012**;9:357.

106. Flynn JM, Hubley R, Goubert C, Rosen J, Clark AG, Feschotte C, et al. RepeatModeler2 for automated genomic discovery of transposable element families. Proc Natl Acad Sci USA. **2020**;117:9451.

107. Bao Z, Eddy SR. Automated de novo identification of repeat sequence families in sequenced genomes. Genome Res. **2002**;12:1269-76.

108. Price AL, Jones NC, Pevzner PA. *De novo* identification of repeat families in large genomes. Bioinformatics. **2005**;21:i351-i8.

109. Smit AFA, Hubley R, Green P. RepeatMasker Open-4.0. available at http://wwwrepeatmaskerorg. **2013**;

110. Stanke M, Morgenstern B. AUGUSTUS: a web server for gene prediction in eukaryotes that allows user-defined constraints. Nucleic Acids Res. **2005**;33:W465-W7.

111. Korf I. Gene finding in novel genomes. BMC Bioinformatics. **2004**;5:59.

112. Dobin A, Davis CA, Schlesinger F, Drenkow J, Zaleski C, Jha S, et al. STAR: ultrafast universal RNA-seq aligner. Bioinformatics. **2013**;29:15-21.

113. Holt C, Yandell M. MAKER2: an annotation pipeline and genome-database management tool for second-generation genome projects. BMC Bioinformatics. **2011**;12:491.

114. Chan PP, Lowe TM. tRNAscan-SE: Searching for tRNA Genes in Genomic Sequences. Methods Mol Biol. **2019**;1962:1-14.

115. Geneious: https://www.geneious.com. (2020).

116. Vizueta J, Sánchez-Gracia A, Rozas J. Bitacora: a comprehensive tool for the identification and annotation of gene families in genome assemblies. Mol Ecol Res. **2020**;20:1445-52.

117. Katoh K, Standley DM. MAFFT multiple sequence alignment software version 7: improvements in performance and usability. Mol Biol Evol. **2013**;30:772-80.

118. Nguyen L-T, Schmidt HA, von Haeseler A, Minh BQ. IQ-TREE: a fast and effective stochastic algorithm for estimating maximum-likelihood phylogenies. Mol Biol Evol. **2015**;32:268-74.

119. Emms DM, Kelly S. OrthoFinder: phylogenetic orthology inference for comparative genomics. Genome Biol. **2019**;20:238.

120. Yu Y, Ouyang Y, Yao W. shinyCircos: an R/Shiny application for interactive creation of Circos plot. Bioinformatics. **2018**;34:1229-31.

121. Yang Y, Li Y, Chen Q, Sun Y, Lu Z. WGDdetector: a pipeline for detecting whole genome duplication events using the genome or transcriptome annotations. BMC Bioinformatics. **2019**;20:75.

122. Wickham H. Ggplot2: elegant graphics for data analysis. New York: Springer-Verlag; 2016.

123. Mendes FK, Vanderpool D, Fulton B, Hahn MW. CAFE 5 models variation in evolutionary rates among gene families. Bioinformatics. **2020**;btaa1022

124. Ponder WF, Lindberg DR. Biology and evolution of the Mollusca. Boca Raton, FL. : CRC press; 2019.

125. Kumar S, Stecher G, Suleski M, Hedges SB. TimeTree: a resource for timelines, timetrees, and divergence times. Mol Biol Evol. **2017**;34:1812-9.

126. Buchfink B, Xie C, Huson DH. Fast and sensitive protein alignment using DIAMOND. Nat Methods. **2015**;12:59-60.

127. Pardos-Blas JR, Irisarri I, Abalde S, Afonso CML, Tenorio MJ, Zardoya R. Supporting data for "The genome of the venomous snail Lautoconus ventricosus sheds light on the origin of conotoxin diversity". GigaScience Database 2021. http://dx.doi.org/10.5524/100892

Legends to figures

Figure 1. Genome organization. The 35 pseudochromosomes of the *L. ventricosus* genome are shown in red. In the inner circles, the distributions of protein-coding genes (black; y-axis indicates percentage of genes per megabase, normalized to 40 genes) and of repetitive elements (green; y-axis indicates percentage of repetitive elements per megabase, normalized to 6,000 repetitive elements) are depicted.

Figure 2. Conotoxin genes. The distribution of the conotoxin precursor (red), hormone (blue), and venom-related protein (green) genes in the 35 pseudochromosomes is shown. Genes closer than 2 Mb were cluster together and their number annotated in brackets. A cone snail sketch (drawn by Lara de la Cita) highlighting (from left to right) the siphon (orange), proboscis (red), radular sac (brown), and the duct (yellow) and bulb (white) of the venom gland is shown.

Figure 3. Conserved synteny and whole genome duplication. A) Conserved synteny between *L. ventricosus* and *Pomacea canaliculata* derived from ortholog proteins. B) Distribution of synonymous divergence (*Ks*) between pairs of paralogs in *L. ventricosus*. The second *Ks* peak indicates the similar divergence between paralogs after the whole genome duplication. C) Annotation of *hox* and *parahox* clusters in *L. ventricosus* and comparison with other available gastropod genomes within a phylogenetic framework.

Figure 1

Click here to access/download;Figure;Fig1.pdf

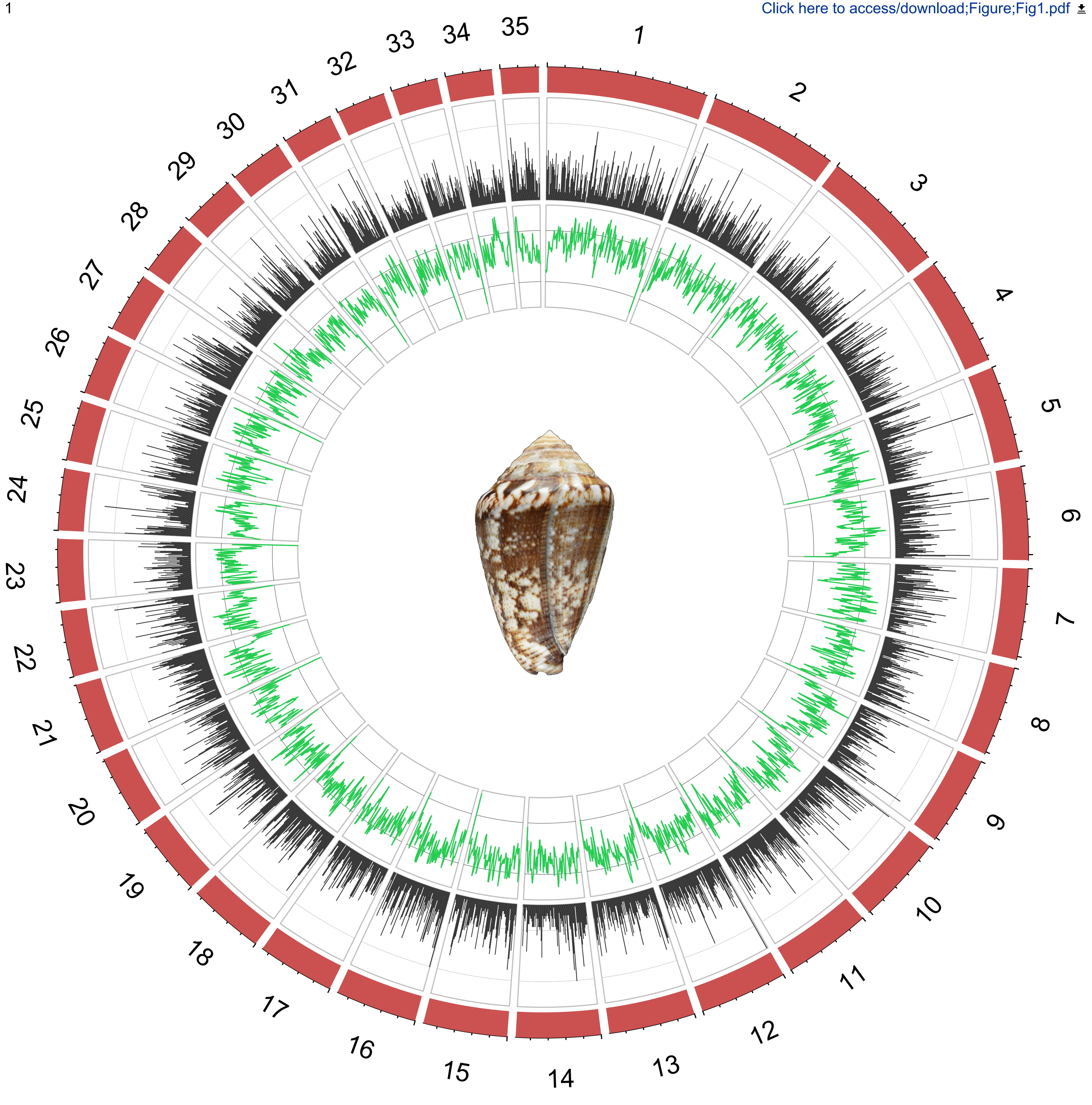

Figure 2

Click here to access/download;Figure;Fig2.pdf

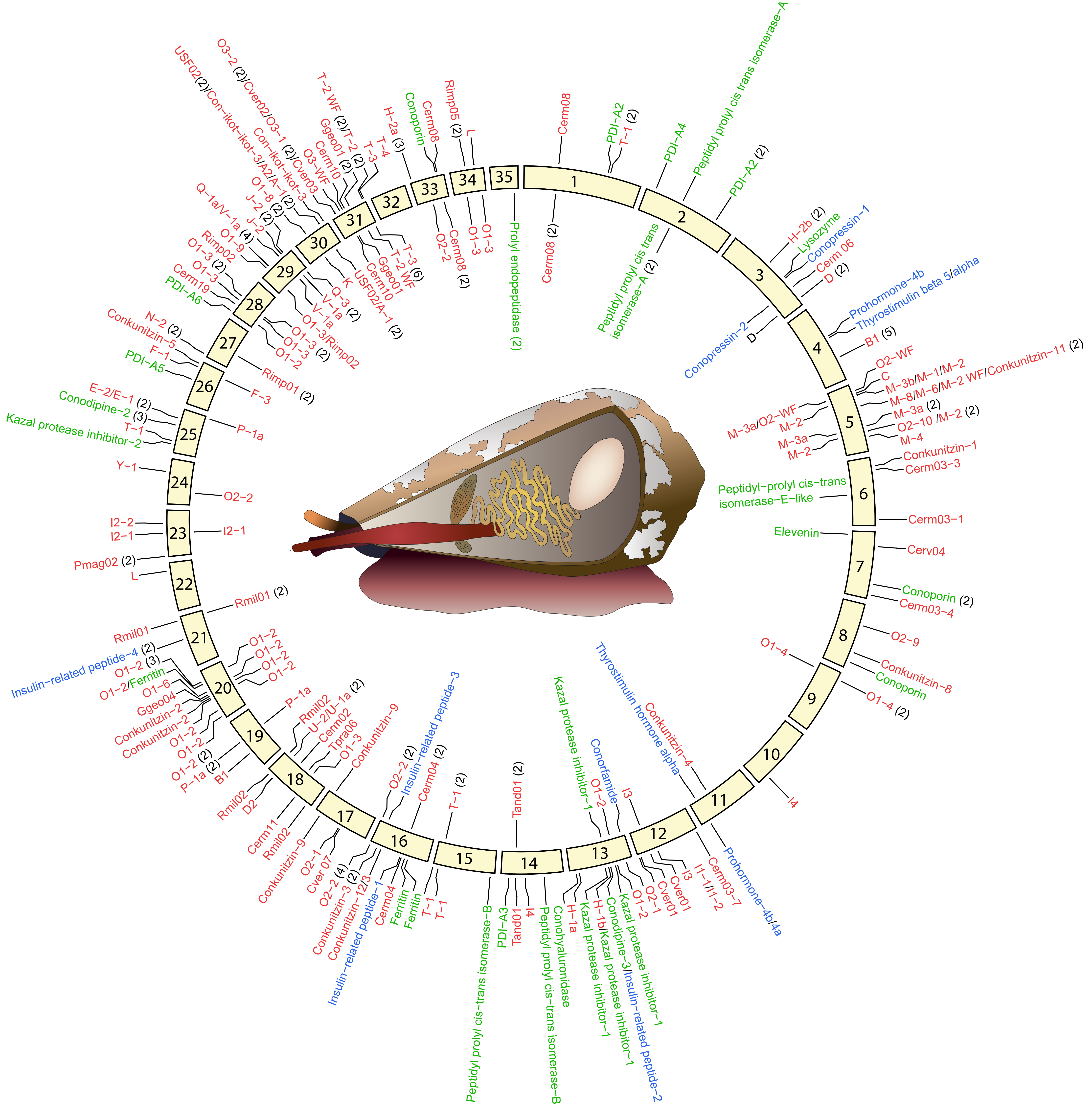

Figure 3

Click here to access/download;Figure;Fig3.pdf

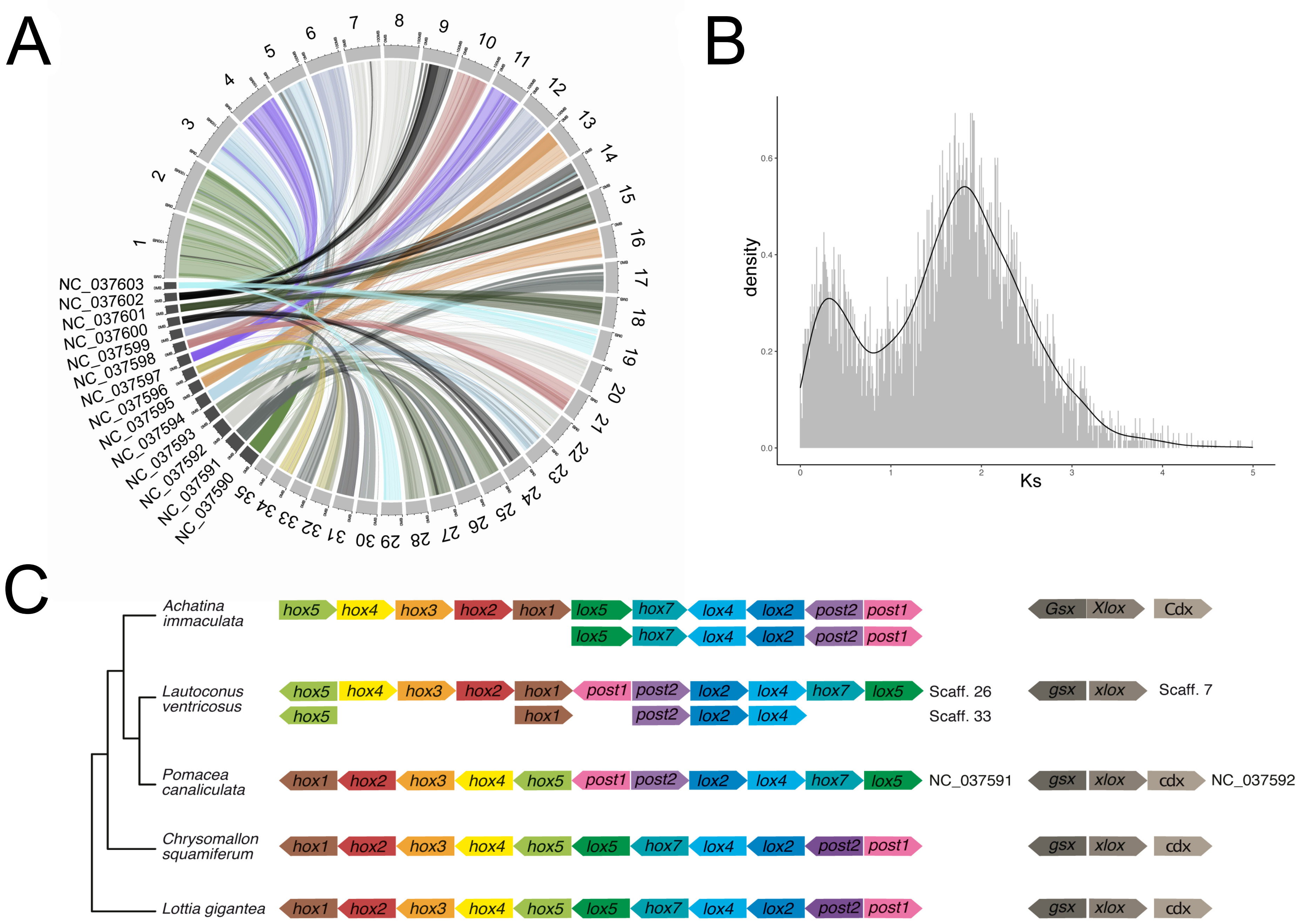

Click here to access/download
Supplementary Material
Legends to Supplementary Figures.docx

Click here to access/download
Supplementary Material
Supp FigS1.pdf

Click here to access/download
Supplementary Material
Supp FigS2.pdf

Supp FigS3

Click here to access/download

Supplementary Material

Supp FigS3.pdf

Supp FigS4

Click here to access/download
Supplementary Material
Supp FigS4.pdf

Click here to access/download
Supplementary Material
Supp FigS5.pdf

Supp FigS6

Click here to access/download
Supplementary Material
Supp FigS6.pdf

Supp FigS7

Click here to access/download
Supplementary Material
Supp FigS7.pdf

Supp FigS8

Click here to access/download
Supplementary Material
Supp FigS8.pdf

Supp FigS9

Click here to access/download
Supplementary Material
Supp figS9.pdf

Supp FigS10

Click here to access/download
Supplementary Material
Supp figS10.pdf

Click here to access/download
Supplementary Material
Supp TableS1.xlsx

Click here to access/download
Supplementary Material
Supp TableS2 new.xlsx

Click here to access/download
Supplementary Material
Supp TableS3.xlsx

Click here to access/download
Supplementary Material
Supp TableS4.xlsx

Supp TableS5

Click here to access/download
Supplementary Material
Supp TableS5.xlsx

Click here to access/download
Supplementary Material
Supp File S1.txt

Supp FileS2

Click here to access/download
Supplementary Material
Supp File S2.txt

Supp FileS3

Click here to access/download
Supplementary Material
Supp File S3.gff3

Supp FileS4

Click here to access/download
Supplementary Material
Supp File S4.txt
