# Supplementary material for: The genome of the venomous snail Lautoconus ventricosus sheds light on the origin of conotoxin diversity
Source: Gigascience. 2021 May 25;10(5):giab037. doi: 10.1093/gigascience/giab037 (PMC8152183; doi:10.1093/gigascience/giab037)
Supplement: giab037_Supplemental_Files [file giab037_supplemental_files.zip › Legends to Supplementary Figures.docx]

Legends to Supplementary Figures

Fig. S1. DNA sources in the *Lautoconus ventricosus* genome. Potential sources of DNA contamination were checked with Blobtools v1.1 using the NCBI entries of viruses, archaea, bacteria, fungi, nematodes, platyhelminthes, polychaetes and human. NCBI entries for mollusks were used for the taxonomic identification of *L. ventricosus* contigs.

Fig. S2. Link density histogram showing the 35 larger scaffolds of the *Lautoconus ventricosus* genome assembly. The x and y axes give the mapping positions of the first and second read in the read pair respectively, grouped into bins. The color of each square gives the number of read pairs within that bin. White vertical and black horizontal lines denote borders between scaffolds. Scaffolds less than 1 Mb were excluded. Figure provided by Dovetail genomics.

Fig. S3. Pseudochromosome sizes. Histogram showing the sizes (in bp) of the 35 largest scaffolds in the *Lautoconus ventricosus* genome assembly.

Fig. S4. Relative expression of venom-related transcripts in the venom gland (CV8; blue) and the foot (CV10 and CV19; orange). Histograms showing the relative expression, normalized in transcripts per kilobase million (TPM), of conotoxin precursor, hormone, and other venom-related protein transcripts.

Fig. S5. Annotation of venom-related genes in the 35 pseudochromosomes of the *L. ventricosus* genome assembly. A pie chart shows the number of venom-related genes identified based on BLAST searches with the *L. ventricosus* venom gland transcriptome (CV8) and the venom gland transcriptomes of closely related cone species endemic to Cabo Verde (Abalde et al. 2020) as queries (dark and light colors, respectively). The number of automatically and manually completed genes as well as of partial genes (with > 1 exon) and single exons is shown. A second pie chart shows how several genes were manually completed with sequences from smaller scaffolds, contigs, and reads. A total of 12 genes were completed by forcing the combination of misassembled, scattered hits i.e., distantly located (sometimes separated by other genes) or reciprocally inverted within a pseudochromosome.

Fig. S6. Distribution of complete venom-related genes in the 35 pseudochromosomes. The regression analysis indicates that the number of venom-related genes was independent of the length (in bp) of the pseudochromosome.

Fig. S7. Correspondence between 3-exon conotoxin precursor genes and protein domains. The boundaries of the three exons of the genes and the three domains (signal, propeptide, and mature)of the encoded protein were compared. An example in which these boundaries did not coincide is shown.

Fig. S8. Intron lengths of 3-exon conotoxin precursor genes. Violin plots estimated using the package Seaborn (https://seaborn.pydata.org/), showing the probability density of the length (in bp) of introns 1 and 2.

Fig. S9. Whole genome duplication in the genome of *Lautoconus ventricosus*. Conserved synteny between each of the 14 *Pomacea canaliculata* pseudochromosomes (red numbers) and the 35 *L. ventricosus* pseudochromosomes (black numbers) was inferred using pairs of 1:1 and 1:2 orthologs obtained with Orthofinder v2.3.11. Synteny plots were generated with the shinyCircos.

Fig. S10. Significant expansion and contraction of gene families in gastropods. A dated tree was built from the current consensus on gastropod phylogeny (Ponder and Lindberg 2019) and median divergence times from the timetree.org database (Kumar et al. 2017). A total of 11,990 orthogroups were inferred with Orthofinder v2.3.11 using all annotated proteins from six gastropod genomes. Gene family significant expansions and contractions were inferred using CAFE v5.0. Those orthogroups containing genes annotated as related to transposable elements were discarded from further study. The remaining orthogroups were mapped onto the phylogeny.
