# Supplementary material for: The genome of the venomous snail Lautoconus ventricosus sheds light on the origin of conotoxin diversity
Source: Gigascience. 2021 May 25;10(5):giab037. doi: 10.1093/gigascience/giab037 (PMC8152183; doi:10.1093/gigascience/giab037)
Supplement: giab037_Supplemental_Files [file giab037_supplemental_files.zip › Supp FigS7.pdf]

Fig. S7. Correspondence between 3-exon conotoxin precursor genes and protein domains

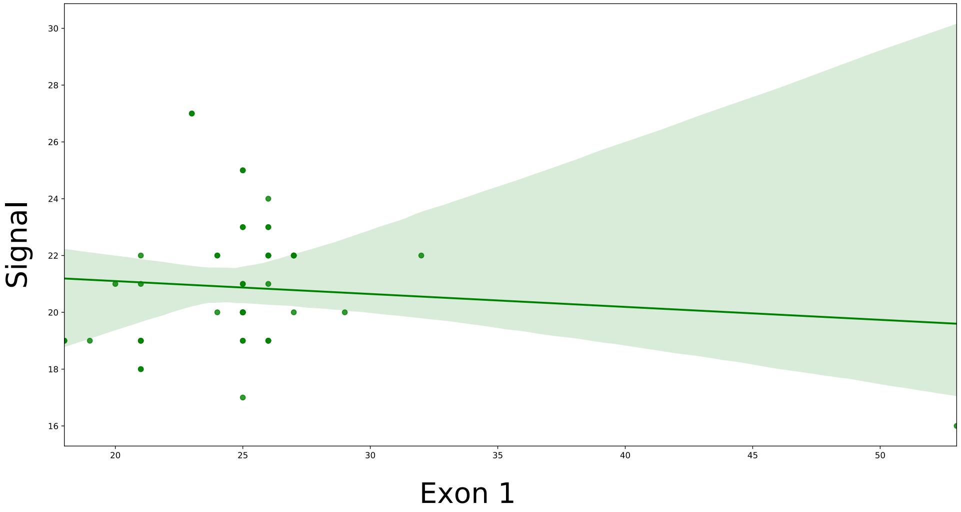

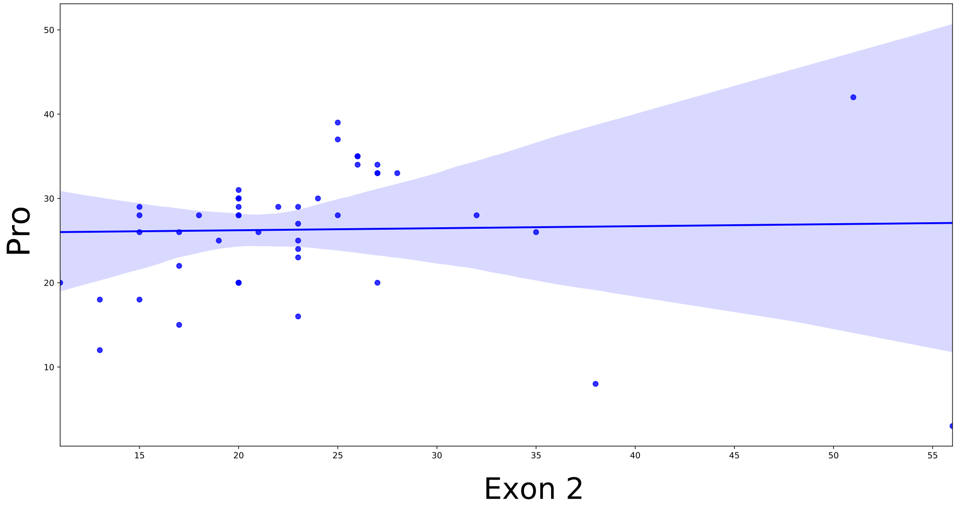

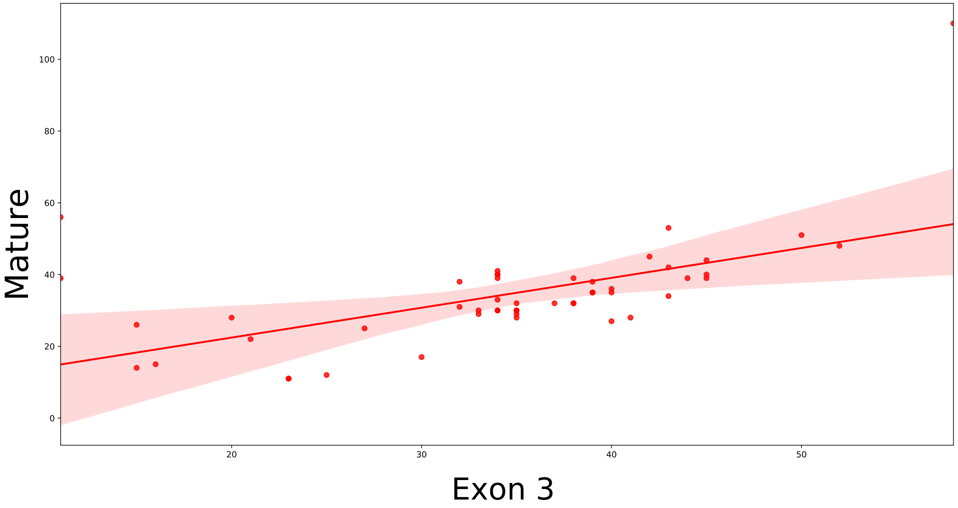

[Superfamily O1-2]

CV8_085

MMKLTCVLIIAVLFLTACQLTTAETRDKQEDPVVRSSDEMQRSEDPKLAKRCGGFGAYCDIGNHNCCSGKCFGFPELAVCT*

EXON 1 EXON 2 EXON 3

GENM_231
