# Supplementary figures and images for: The genome of the venomous snail Lautoconus ventricosus sheds light on the origin of conotoxin diversity
Source: Gigascience. 2021 May 25;10(5):giab037. doi: 10.1093/gigascience/giab037 (PMC8152183; doi:10.1093/gigascience/giab037)

**Fig. S1. DNA sources in the *Lautoconus ventricosus* genome**

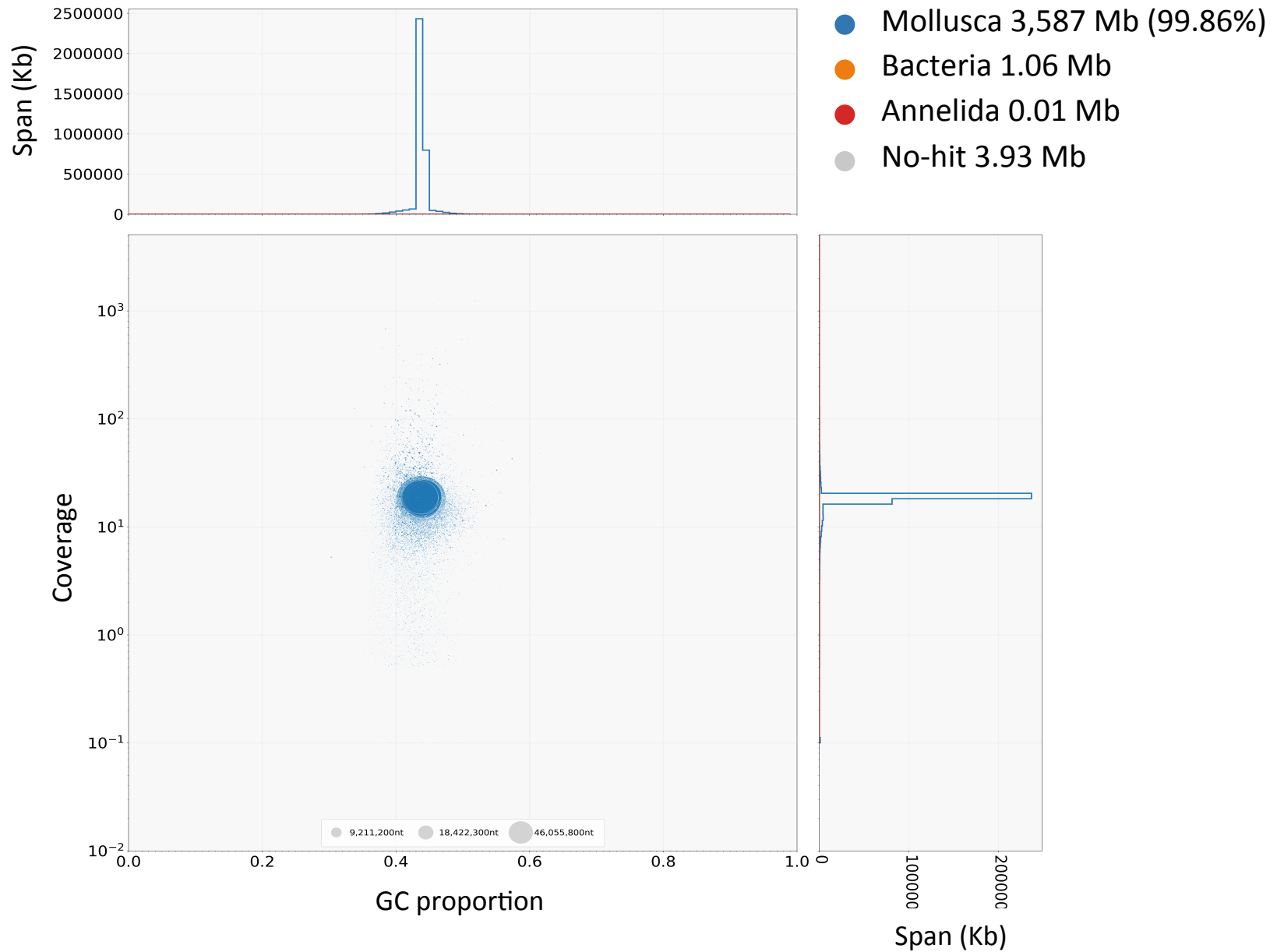

Supplement: giab037_Supplemental_Files [file giab037_supplemental_files.zip › Supp FigS1.pdf]

**Fig. S10. Significant expansion and contraction of gene families in gastropods**

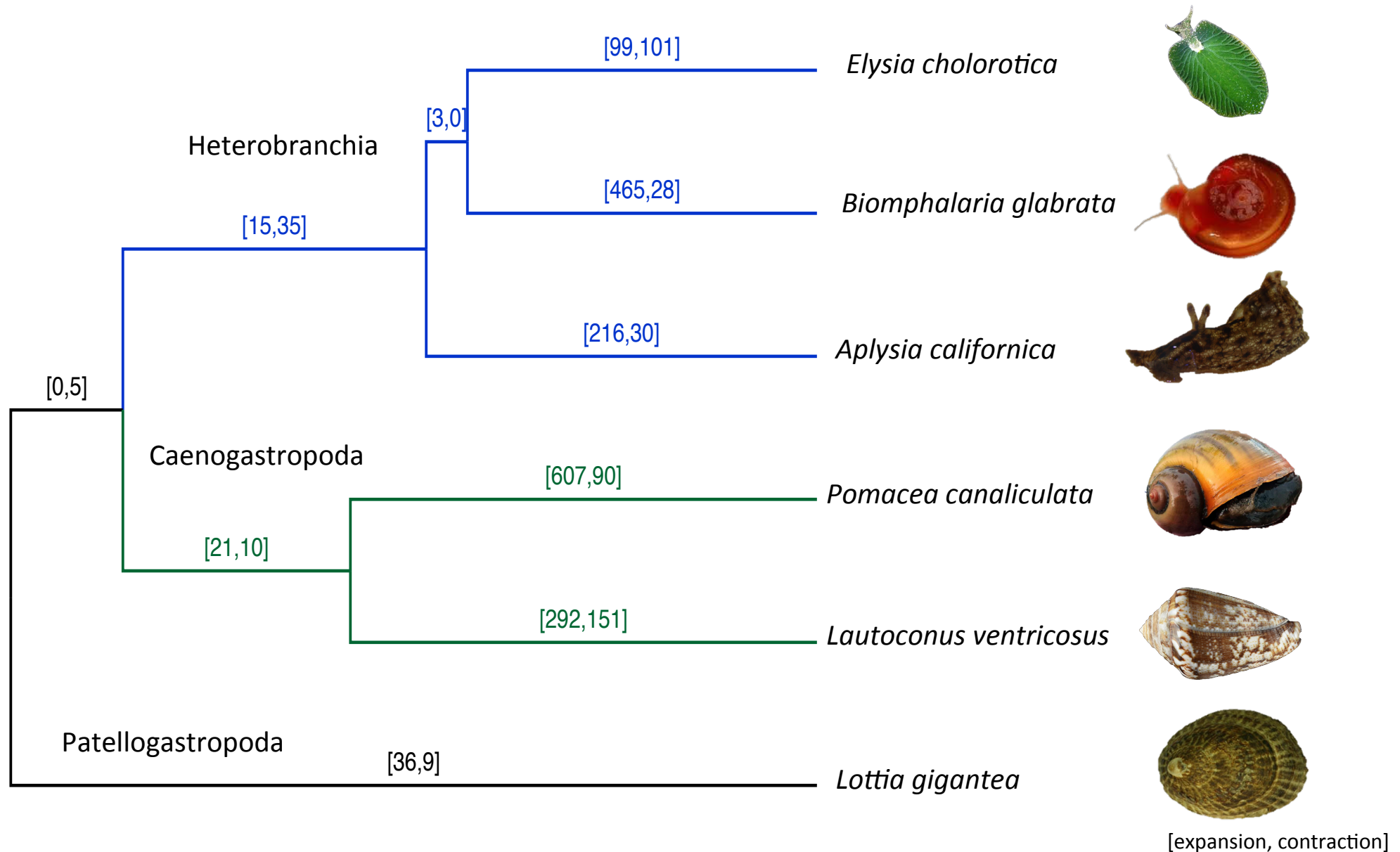

Supplement: giab037_Supplemental_Files [file giab037_supplemental_files.zip › Supp figS10.pdf]

**Fig. S2. Link density histogram (35 scaffolds)**

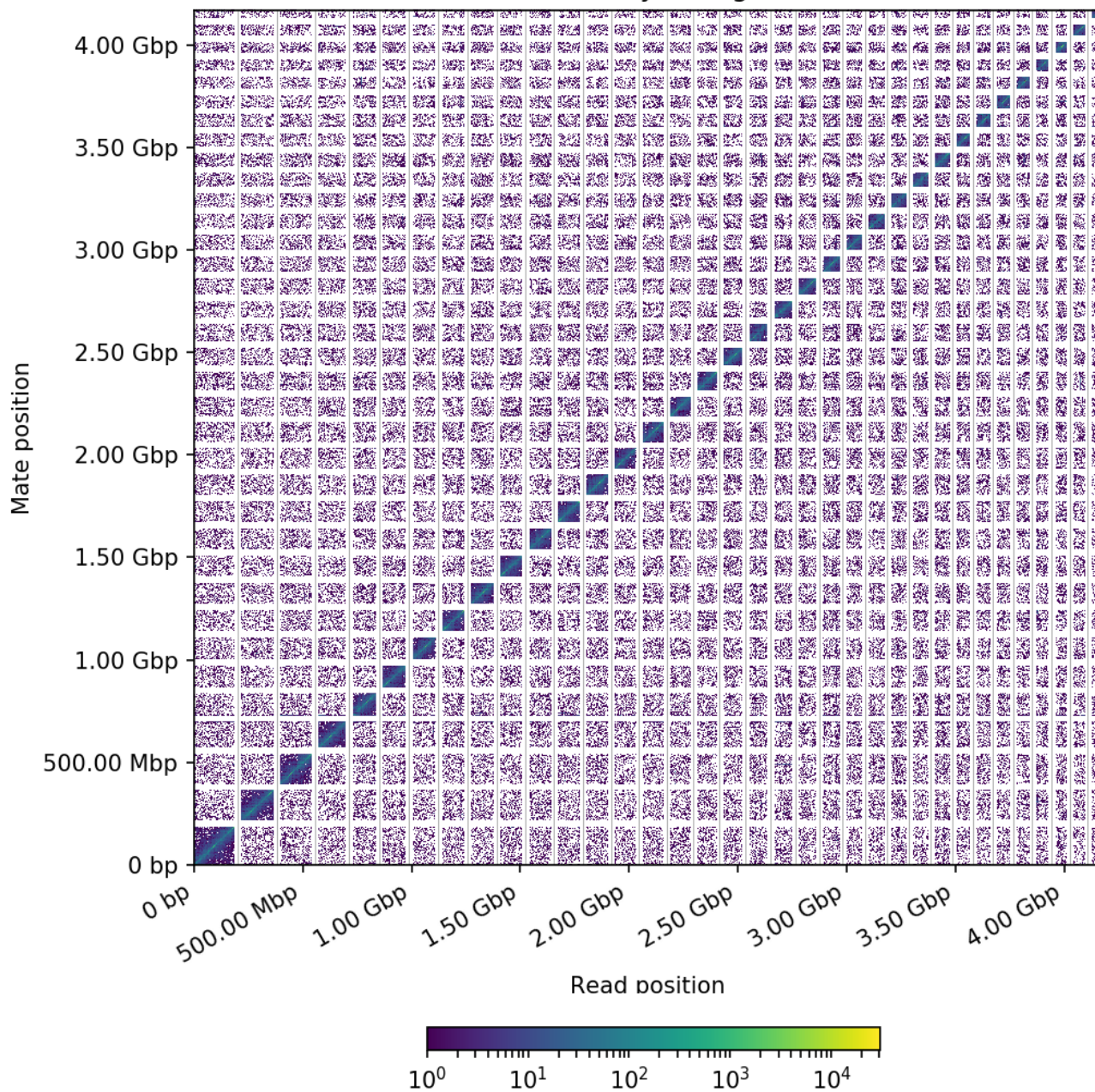

Supplement: giab037_Supplemental_Files [file giab037_supplemental_files.zip › Supp FigS2.pdf]

**Fig. S3. Pseudochromosome sizes**

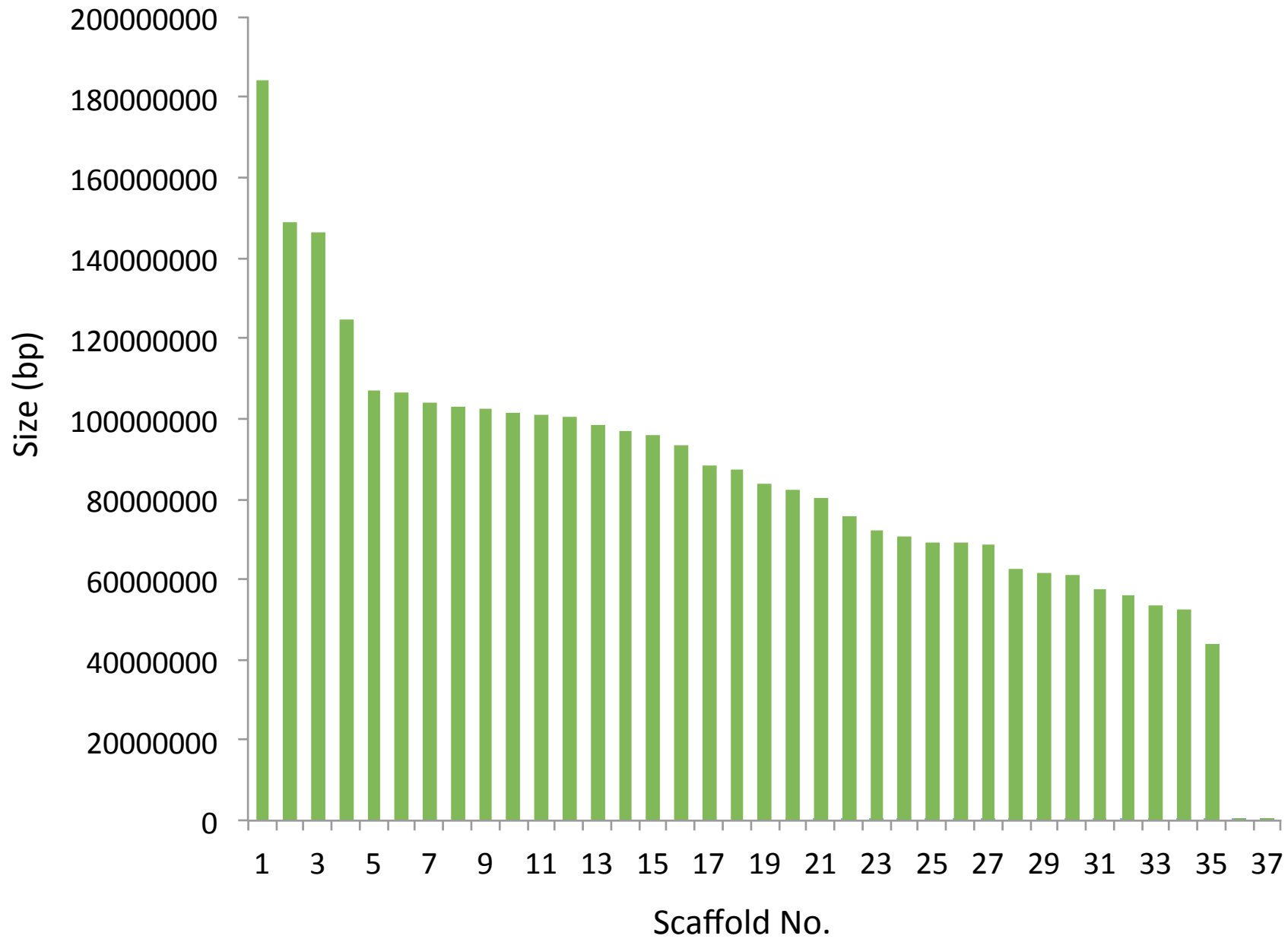

Supplement: giab037_Supplemental_Files [file giab037_supplemental_files.zip › Supp FigS3.pdf]

Fig. S4. Relative expression of venom-related transcripts in the venom gland and the foot

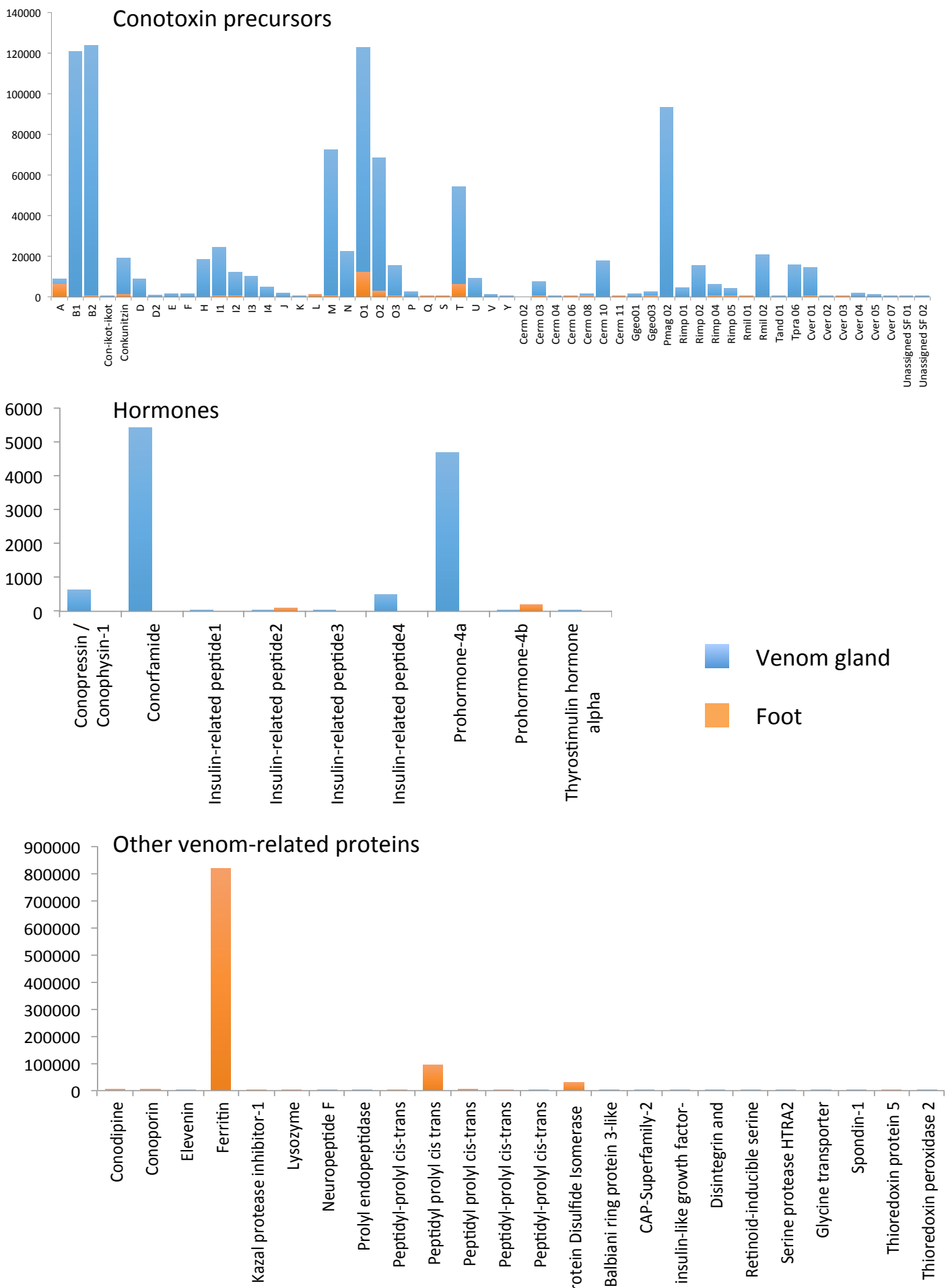

Supplement: giab037_Supplemental_Files [file giab037_supplemental_files.zip › Supp FigS4.pdf]

**Fig. S5. Annotation of venom-related genes in the *L. ventricosus* genome**

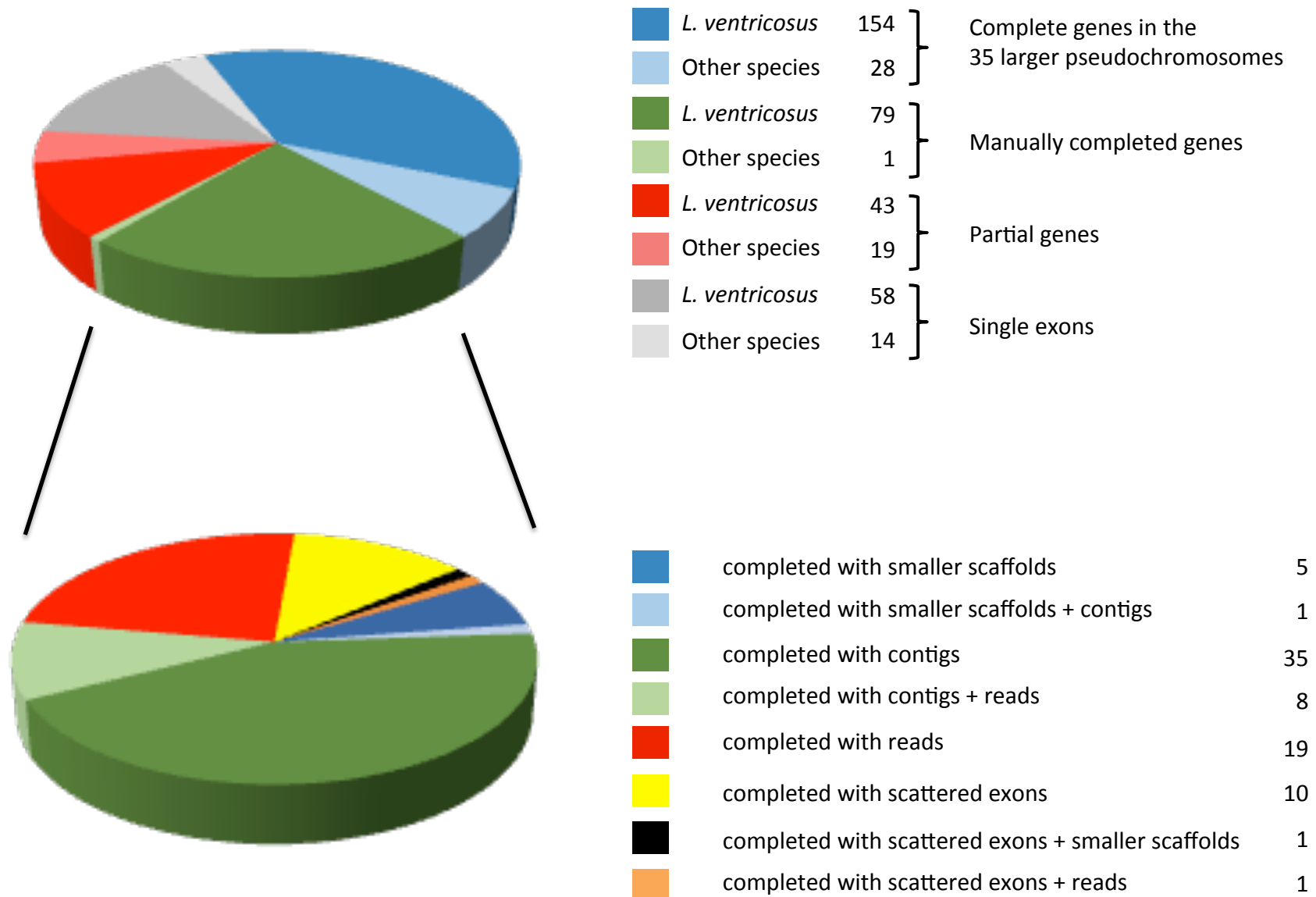

Supplement: giab037_Supplemental_Files [file giab037_supplemental_files.zip › Supp FigS5.pdf]

**Fig. S6. Distribution of complete venom-related genes per pseudochromosome**

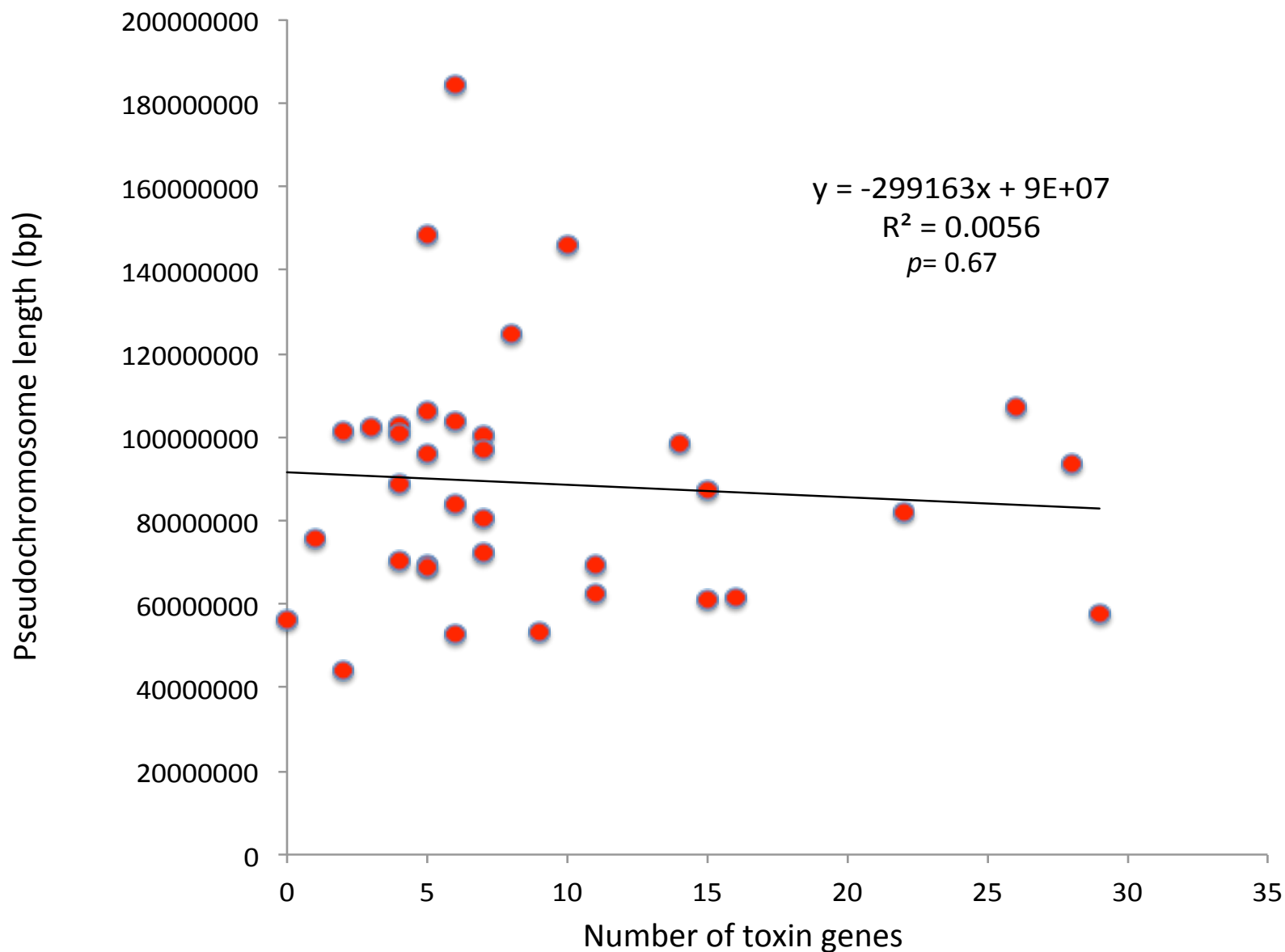

Supplement: giab037_Supplemental_Files [file giab037_supplemental_files.zip › Supp FigS6.pdf]

**Fig. S8. Intron lengths of 3-exon conotoxin precursor genes**

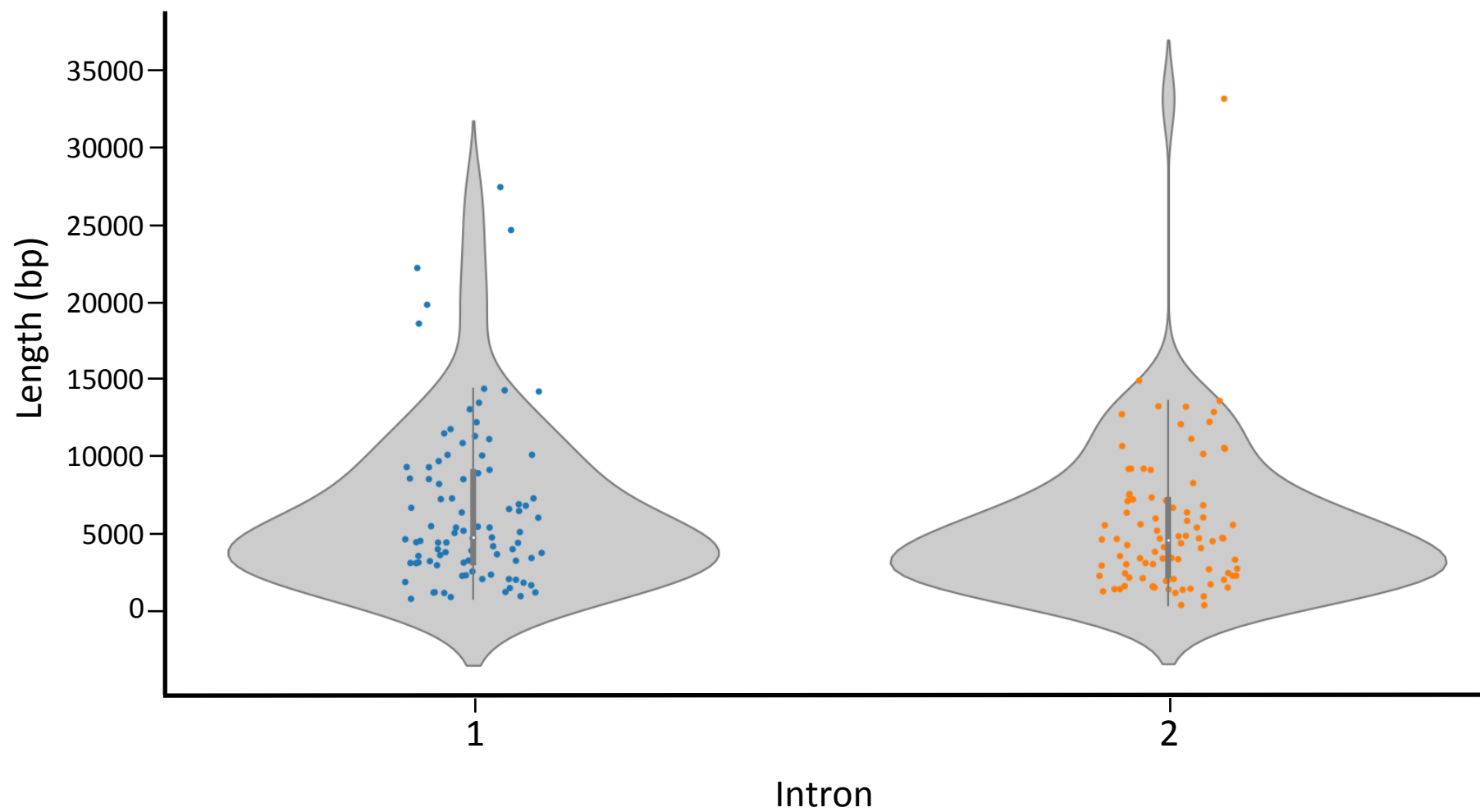

Supplement: giab037_Supplemental_Files [file giab037_supplemental_files.zip › Supp FigS8.pdf]

**Fig. S9. Whole genome duplication in the genome of *Lautoconus ventricosus***

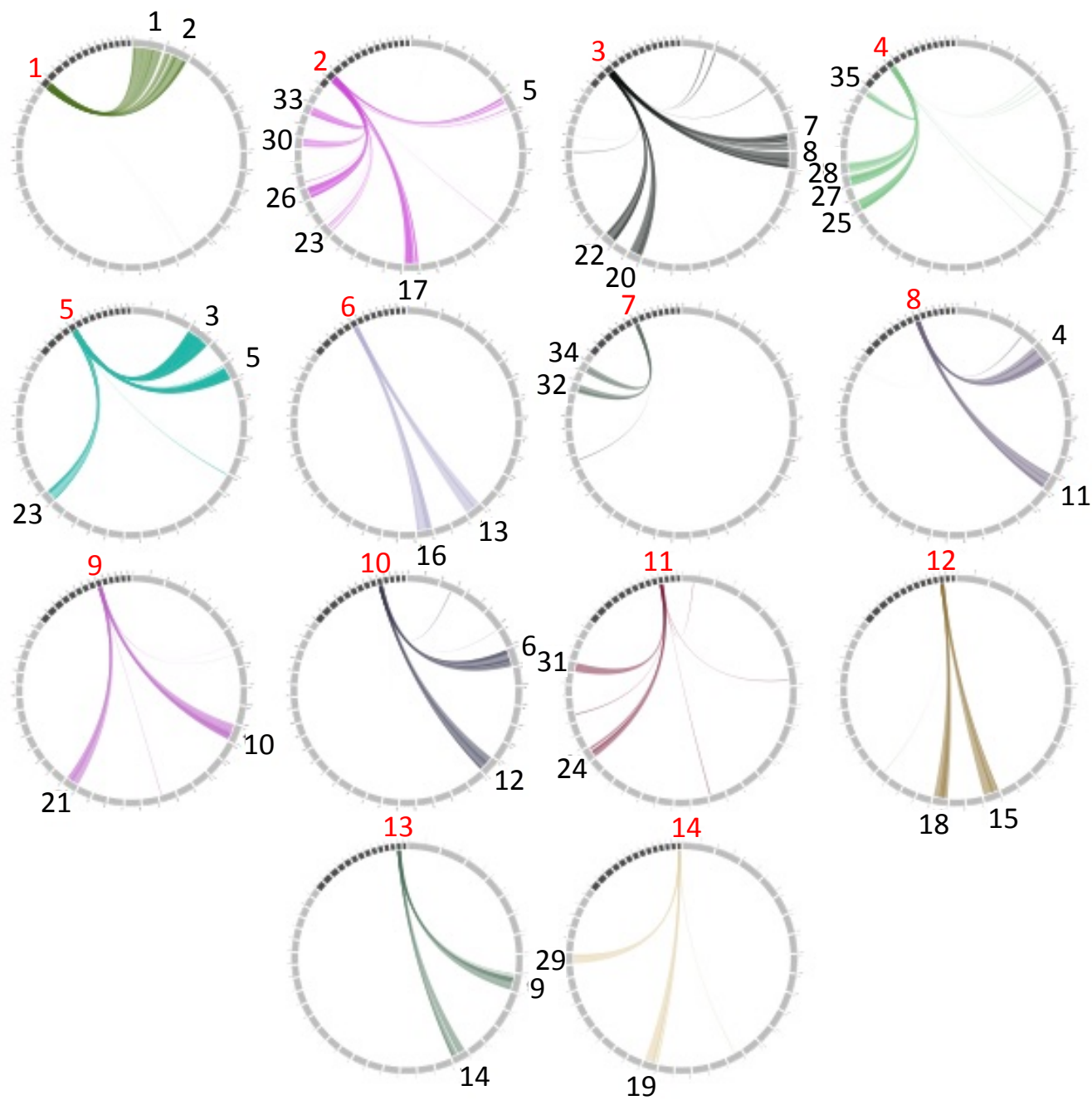

Supplement: giab037_Supplemental_Files [file giab037_supplemental_files.zip › Supp figS9.pdf]
