# Supplementary material for: The genome of the venomous snail Lautoconus ventricosus sheds light on the origin of conotoxin diversity
Source: Gigascience. 2021 May 25;10(5):giab037. doi: 10.1093/gigascience/giab037 (PMC8152183; doi:10.1093/gigascience/giab037)
Supplement: giab037_Response_to_Reviewer_Comments_Original_Submission [file giab037_response_to_reviewer_comments_original_submission.pdf]

Author's Response To Reviewer Comments

Close

RESPONSE TO REVIEWERS

Editor
I'd encourage you to upload additional supporting data, such as software input/output files, "intermediate assemblies", annotations etc, as mentioned by the referee, to our FTP server. We will release the supporting data set with a stable Digital Object Identifier via our repository GigaDB, alongside the paper.

Following the suggestion of the reviewer we have uploaded in GigaDB, the original PacBio assembly and bioinformatics commands (the final assembly, annotation files, predicted transcript and protein sequences were already in GigaDB). We have also updated the Readme and md5sum files with the new data.

Reviewer #1
This manuscript, entitled « The genome of the venomous snail Lautoconus ventricosus sheds light on the origin of conotoxin diversity » by Pardos-Blas et al., provides a high quality chromosome-level genome of a Conidae. It is very unfortunate for the authors that another cone snail genome has been published the very next day I received this paper for review (Peng C et al., Cell Discovery 2021). In any case, while taking a bit of the novelty out, I see these two papers as complementary rather than directly competing. Here, the genome data were complemented with venom gland transcriptome data and overall, the authors have performed a detailed analysis of their genome and transcriptome data. I enjoyed reading this manuscript, which is very well written, and nicely illustrated. Congratulations to the authors for a remarkable achievement. I recommend publication as I only have some minor comments.

We thank the reviewer for the positive feedback. As the referee mentions, during the review process, the genome assembly of Dendroconus betulinus was published. We now acknowledge this paper after the conclusions and mention the congruency found between their and our results. The following paragraph was added:

"During the review process of this article, the genome assembly of the vermivorous cone snail Dendroconus betulinus was reported [76], showing results highly congruent with our findings. The genome of this species was of similar size and assembled also into 35 major scaffolds, and up to 133 conotoxin precursor genes were identified and located in the different scaffolds [76]. As in the case of L. ventricosus, the ratio of conotoxin precursor genes and transcripts in D. betulinus was close to one, and thus it was inferred that the high diversity of functional conotoxins is achieved at the post-translational level [76]. The assembly of two chromosome-level genomes of cone snails opens the door to fruitful comparative genomic studies aimed at further understanding the origin and evolution of conotoxin diversity."

- Just a general comment: I wonder why the authors did not use the same specimen for both genomics and transcriptomics ? Cone snail venoms are notoriously hypervariable, including significant intra-specific variations. This is particularly true for Conus ventricosus (as demonstrated by Romeo C et al., J Sep Sci, 2008, this should be cited), and therefore I am wondering how much of this variation may account for the discrepancy between the number of conotoxin genes vs transcripts. The authors may want to comment on this.

The venom gland transcriptomes were obtained from specimens captured in 2012 in Olhão, Portugal. At that time the possibility of sequencing the genome was not anticipated and the body was preserved in ethanol 100%, which does not render HMW DNA. The PacBio reads and the llumina/ HiC data were obtained from specimens captured in 2017 also in Olhão, Portugal. We used large quantities of tissue (including the venom gland) to purify HMW DNA and during three years we solved many technical problems to obtain the genome. Focus on that, we neither preserve apart the venom glands nor used

them to obtain transcriptomes, which should have been the best strategy, as we know now. The reviewer is right and not only conotoxins may vary among individuals (although many reported variations are not at the sequence level but at the post-translational modifications of the peptide) but also there are important levels of heterozigosity for all genes. It is possible that part of the discrepancy between the number of conotoxin genes versus transcripts may be due to inter-individual variation, which has been reported among L. ventricosus populations (Romeo et al., 2008). Now, we mention that possibility in the main text and also cite the suggested paper.

- I agree with the title that this work sheds light on conotoxin diversity, but to me, another unanswered question remains: the origin(s) of conotoxin genes. For hormone-like venom peptides (conoinsulin, conopressin…etc), duplication/neofunctionalization of the endogenous hormone gene can be put forward as the most likely event for their presence in the venom gland, but to my knowledge, nothing « endogenous » ressembles the disulfide-rich conotoxins. Where do they come from?

As we mention in the text "To explain the evolutionary origin … it has been suggested that toxin genes could emerge either through gene duplication and adaptive neofunctionalization of physiological genes in the venom gland coupled with reduction of expression levels in other tissues or alternatively by subfunctionalization through neutral evolution and restriction to the venom gland. This statement includes conotoxins and hormones. Given the fast evolutionary rates of conotoxins it is not surprising that it is very difficult to identify the original endogenous proteins that evolved into conotoxins, but the fact that their function is to inhibit ion channels in the neuromuscular system of the prey, point to an origin from (or a convergence to) neuropeptides.

- Interestingly, the authors find conotoxins genes in the foot transcriptome, although expressed at low levels. They comment page 3 that « this is the first report of conotoxin expression outside the venom gland in cone snails ». Not quite true, as Biggs JS et al. (Toxicon. 2008) have reported alpha-conotoxins specifically expressed in the salivary gland of Conus pulicarius, and more recently, Dutertre S et al. (Nat Comm, 2014) have reported conotoxin expression in the radular sac of Conus geographus. Nonetheless, this finding raises several questions. First, are those conotoxin transcripts the same as those found in the venom gland (Dutertre S et al. found different transcripts in the radular sac, possibly pseudogenes) ? Second, I understand that it is highly speculative, but could the authors discuss the putative role of these transcripts in the foot? They are expressed at low levels, but could they have an antimicrobial role (like many molluscs, the foot of cone snail produces mucus, and such antimicrobial agents have been found in other gastropods)? Finally, some hormone-like transcripts were found more highly expressed in the foot compared to the venom gland, could these represent the endogenous hormones?

The reviewer is right, and the expression of few conotoxin transcripts had been reported in other cone species. We now cite the two cases mentioned by the reviewer. In addition, we have added a Venn diagram to Supplementary table S2 to show the exact transcripts that were shared by all transcriptomes or by each of the foot transcriptomes and the venom gland transcriptome. Nevertheless, given the variability of the conotoxin precursors, in the main text we discuss comparisons at the superfamily level rather than transcript-by-transcript. Finally, following the suggestion of the reviewer, we now mention the possibility that the conotoxin transcripts in the foot may have antimicrobial activity as reported for some conotoxins found in other cone species (Figueroa-Montiel et al. 2018 and Bernáldez-Sarabia et al. 2019). As suggested by the reviewer, we now state: "these hormones and proteins may be endogenous, having a physiological function common to different tissues and not restricted to the venom gland".

Typos and other small errors:

- page 3 : I would invert the order (smallest to largest) of the sentence : « …from 184 to 44 Mb ».
- page 4, last para : « The gene structures of found… ».
- page 6, top left : « The comparison…illustrate… » should be « illustrates ».
- page 7, second para : « …have been involved IN the differenciation… ».
- page 7, third para : « …have separate sexes OR be hermaphrodite. »

All changed as suggested!

Reviewer #2
In this study, Ramón Pardos-Blas et al. report a chromosome-level geome assembly for the Mediterranean cone snail. This is an impressive achievement given the large (3.59 Gb) size of this genome. I think this genome will be a valuable resource for the molluscan genomics community, especially for those interested in gastropods (as only one high-quality caenogastropod genome is

available) and venom evolution. I do have a number of mostly minor questions and comments that I think should be addressed.

We thank Kevin Kocot for his insightful feedback

It is stated that the genome annotations contain 89.2% of the BUSCO metazoan_odb10 genes but it is not stated what the BUSCO score of the scaffolded genome assembly itself is in the main text. This would be helpful in assessing the completeness of the gene models relative to the genome assembly. Likewise please also report all of the BUSCO metrics (e.g., percent fragmented and duplicated) for both the scaffolded genome and gene models in the main text.

We thank the reviewer for pointing this issue: Actually, we made a mistake here and what we were reporting was the BUSCO metric for the scaffolded genome and not for the gene models. We have corrected that and provided the metrics for the genome assembly and the gene models including percentages of complete duplicated and fragments. For the genome: complete single-copy, 82%; complete duplicated, 2.9%; fragmented, 4.3%. For the gene models: complete single-copy, 31.2%; complete duplicated, 0.4%; fragmented, 21.7%.

The somewhat low BUSCO score for the gene models is attributed to "likely due to the much larger genome size of L. ventricosus" (with respect to other gastropods with higher BUSCO scores). However, the rationale behind this statement is not explained.

This statement was deleted

On page 3, it is stated that "The main methodological limitation that may explain the missing loci would be the error rate associated to the PacBio CLR sequencing technology (15% [47]), which despite partially corrected by coverage, would hamper BLAST similarity searches." Was any attempt made to polish the genome with the short reads using e.g., Pilon or POLCA?

As explained in the materials and methods section, the assembly of the genome was performed based exclusively on the PacBio long reads from one individual. The Illumina reads from another individual were used for the Chicago and HiC scaffolding. Due to the heterozygosity between individuals, it was not possible to use these Illumina reads to polish the PacBio-based genome assembly.

What is the heterozygosity of the genome? This should be reported in the main text.

In order to estimate heterozygosity, we used GenomeScope 2.0. However, in the original paper of the software (Vurture et al., 2017), the authors warned: “Oxford Nanopore or Pacific Biosciences, which currently average 5–20% error, are not supported as an error will occur on average every 5–20 bp and thus infer with nearly every k-mer”. Alternatively, we used the Illumina raw reads of the transcriptomes (CV8, CV10, CV19) to estimate it. The final values of heterozygosity were 1.05-1.08%. This is now mentioned and discussed in the main text.

Was any attempt made to purge redundant haplotigs from the initial long-read assembly prior to scaffolding?

As explained in Materials & Methods, the long-read assembly was obtained using wtdbg2. This software follows the Overlap Layout Consensus methodology, which computes the consensus haploid sequence of each contig. Wtdbg2 produces less false duplications than other assemblers (Ruan and Li, 2020), so no attempt to purge duplicates was made. Nonetheless, motivated by the suggestion of the reviewer, we ran Purge Haplotigs (Roach et al., 2018) to detect false duplications and obtained a single peak in the read-depth histogram indicating low levels of duplicates, which were also evidenced by the BUSCO results (2.9% duplicates).

On page 3 it is stated that "The percentage of complete venom-related loci detected is considerably lower than expected according to general BUSCO results (89.2%). The extra 15% of transcripts of the transcriptome without a gene counterpart in the genome could be isoforms that could be produced naturally during expression or generated as artifacts during transcriptome assembly." Perhaps the Oyster River Protocol or this (admittedly unpublished) approach should be tested? https://www.biorxiv.org/content/10.1101/2021.02.18.431773v1

Following the suggestion of the reviewer, we ran TransPi (Rivera-Vicéns et al., 2021). This program uses various assemblers and k-mers to generate a non-redundant consensus de novo transcriptome. The resulting L. ventricosus venom gland transcriptome had BUSCO scores (C:69.0% [S:60.8%,D:8.2%], F:9.7%, M:21.3%, n:954) that were very similar to those obtained using Trinity alone (C:67.6% [S:57.5%,D:10.1%], F:12.5%, M:19.9%, n:954).

Conotoxin transcripts were detected in the foot tissue transcriptome. In the methods, please indicate if these RNA extractions were performed at the same time or not and how the transcriptome libraries were split between the two HiSeq 2000 flowcells. Is it possible there was laboratory contamination or 'bleed through' where some barcodes in the venom gland library were mis-read as barcodes for the foot library, resulting in some reads 'bleeding through' into the foot transcriptome data?

The processing of the venom gland and foot samples was done in different years, and therefore the 'bleeding through' suggested by the reviewer was impossible. The text now reads “Library construction and sequencing of the venom gland transcriptome was conducted at AllGenetics (Oleiros, Spain) in 2012 whereas foot transcriptomes were obtained at Sistemas Genómicos (Valencia, Spain) in 2016.”

I noticed ferritin is highly expressed in the foot. Any idea why?

The reviewer is right that ferritin shows high expression levels in the foot. In this regard, we have added the following comment to the text: “This is a protein that generally regulates the storage and release of iron, and has been related to the incorporation of iron into the radula in some chitons and limpets, and into the shell in the pearl oyster”

I would encourage the authors to make the initial PacBio assembly and as many of the exact commands used and key input/output files for the programs they ran available via e.g., Figshare or Dryad.

Following the suggestion of the reviewer we have uploaded in GigaDB, the original PacBio assembly and bioinformatics commands (the final assembly, annotation files, predicted transcript and protein sequences were already in GigaDB). We have also updated the Readme and md5sum files with the new data.

The methods state (on page 8) that "After scaffolding, shotgun sequences were used to close gaps between contigs." Not enough detail is provided here to assess what was done.

The procedure is explained in detail in Putnam et al. 2016. We now cite that reference at the end of the sentence. The authors say: “HiRise can use paired-end shotgun reads to close some of the gaps of unknown sequence created when scaffolds are joined based on Chicago read pairs. Groups of reads localized by SNAP alignment to the vicinity of each such gap are passed to “marauder,” the gap-closing module of meraculous, which returns a gap-closing sequence when a unique closure can be inferred by local k-mer walking.

The methods state (on page 9) "Only models that were predicted by both SNAP and AUGUSTUS were retained in the final annotation set." How was this ensured?

The reviewer is right and the sentence was misleading. We now state: “If multiple models predicted by SNAP and AUGUSTUS overlapped, only the one with the lowest AED was retained in the final annotation set”

The statement on the availability of supporting data is not clear on whether the predicted transcripts and proteins from the automated genome annotation are made available as fasta files. Please provide these as well.

We have modified the statement. “Final assembly, original PacBio assembly, as well as annotation files, predicted transcript and protein sequences, and bioinformatics supporting information were deposited in

GigaDB. Additionally, Assembly, PacBio subreads and transcriptome raw data were deposited at NCBI under the bioproject number PRJNA678883. Final assembly (JAFLJL000000000); PacBio subreads (SRR13994261-SRR13994264); RNAseq raw reads (CV8: SRR13740844, CV10: SRR13757741, CV19: SRR13770976)."

Minor points:
Correct "preys" to "prey" in the abstract.

Corrected as suggested

Confirm that all instances of "L. ventricosus" are italicized.

Confirmed!

Page 2, L column, first paragraph: "The latter…" - Does this refer to snakes?

Now, it reads "The snakes…"

Page 2, R column, first paragraph: "newly long-read assembled genome" - this is a bit awkward as written.

"Long-read" was deleted

Page 4, L column, first full paragraph: Add space between "31" and "were"

Added!

Page 5, R column, third paragraph: Please clarify what is meant by "an evolutionary successful group of land snails and slugs" here. Pulmonata? All Heterobranchia?

We now mention that the group is Stylommatophora

Page 7, L column, second paragraph: The detection of the camlbp I gene is noted but its significance is not explained in detail.

Actually, its function is explained together with that of engrailed genes in a previous sentence: "Several genes including engrailed and camlbp I have been involved in the differentiation of a shell field distinct from the mantle tissue"

Page 7, L column, third paragraph: There is an English problem here: "sexes of be hermaphrodite"

Changed to "sexes or be hermaphrodite"

Page 8, L column, RNA extraction, library preparation, and sequencing section: Correct "grinded" to "ground"

Changed as suggested

Page 8, R column, last section: I suggest changing "Genome automated annotation" to just "Genome annotation"

Changed as suggested

Figure 1: The caption does not explain the y-axis for the distributions of protein-coding genes and repetitive elements.

Now, we explain in the caption that the y-axis for the distributions of protein-coding genes and repetitive elements correspond to percentage of genes per megabase (normalized to 40 genes and to 6,000 repetitive elements).

Close
