# Supplementary material for: The genome of the venomous snail Lautoconus ventricosus sheds light on the origin of conotoxin diversity
Source: Gigascience. 2021 May 25;10(5):giab037. doi: 10.1093/gigascience/giab037 (PMC8152183; doi:10.1093/gigascience/giab037)
Supplement: giab037_Reviewer_1_Report_Original_Submission — SÃ©bastien Dutertre -- 3/8/2021 Reviewed [file giab037_reviewer_1_report_original_submission.pdf]

Reviewer Report

Title: The genome of the venomous snail Lautoconus ventricosus sheds light on the origin of conotoxin diversity

Version: Original Submission **Date:** 3/8/2021

Reviewer name: SÃ©bastien Dutertre

Reviewer Comments to Author:

This manuscript, entitled Â« The genome of the venomous snail Lautoconus ventricosus sheds light on the origin of conotoxin diversity Â» by Pardos-Blas et al., provides a high quality chromosome-level genome of a Conidae. It is very unfortunate for the authors that another cone snail genome has been published the very next day I received this paper for review (Peng C et al., Cell Discovery 2021). In anycase, while taking a bit of the novelty out, I see these two papers as complementary rather than directly competing. Here, the genome data were complemented with venom gland transcriptome data and overall, the authors have performed a detailed analysis of their genome and transcriptome data. I enjoyed reading this manuscript, which is very well written, and nicely illustrated. Congratulations to the authors for a remarkable achievement. I recommend publication as I only have some minor comments.
- Just a general comment : I wonder why the authors did not use the same specimen for both genomics and transcriptomics ? Cone snail venoms are notoriously hypervariable, including significant intra-specific variations. This is particularly true for Conus ventricosus (as demonstrated by Romeo C et al., J Sep Sci, 2008, this should be cited), and therefore I am wondering how much of this variation may account for the discrepancy between the number of conotoxin genes vs transcripts. The authors may want to comment on this.
- I agree with the title that this work sheds light on conotoxin diversity, but to me, another unanswered question remains : the origin(s) of conotoxin genes. For hormone-like venom peptides (conoinsulin, conopressin…etc), duplication/neofunctionalization of the endogenous hormone gene can be put forward as the most likely event for their presence in the venom gland, but to my knowledge, nothing Â« endogenous Â» ressembles the disulfide-rich conotoxins. Where do they come from ?
- Interestingly, the authors find conotoxins genes in the foot transcriptome, although expressed at low levels. They comment page 3 that Â« this is the first report of conotoxin expression outside the venom gland in cone snails Â». Not quite true, as Biggs JS et al. (Toxicon. 2008) have reported alpha-conotoxins specifically expressed in the salivary gland of Conus pulicarius, and more recently, Dutertre S et al. (Nat Comm, 2014) have reported conotoxin expression in the radular sac of Conus geographus. Nonetheless, this finding raises several questions. First, are those conotoxin transcripts the same as those found in the venom gland (Dutertre S et al. found different transcripts in the radular sac, possibly pseudogenes) ? Second, I understand that it is highly speculative, but could the authors discuss the putative role of these transcripts in the foot ? They are expressed at low levels, but could they have an antimicrobial role (like many molluscs, the foot of cone snail produces mucus, and such antimicrobial agents have been found in other gastropods) ? Finally, some hormone-like transcripts were found more highly expressed in the foot compared to the venom gland, could these represent the endogenous hormones ?

Typos and other small errors :
- page 3 : I would invert the order (smallest to largest) of the sentence : Â« …from 184 to 44 Mb Â».
- page 4, last para : Â« The gene structures of found… Â».
- page 6, top left : Â« The comparison…illustrate… Â» should be Â« illustrates Â».
- page 7, second para : Â« …have been involved IN the differenciation… Â».
- page 7, third para : Â« …have separate sexes OR be hermaphrodite. Â»

Methods

Are the methods appropriate to the aims of the study, are they well described, and are necessary controls included? Choose an item.

Conclusions

Are the conclusions adequately supported by the data shown? Choose an item.

Reporting Standards

Does the manuscript adhere to the journal's guidelines on minimum standards of reporting? Choose an item.

Choose an item.

Statistics

Are you able to assess all statistics in the manuscript, including the appropriateness of statistical tests used? Choose an item.

Quality of Written English

Please indicate the quality of language in the manuscript: Choose an item.

Declaration of Competing Interests

Please complete a declaration of competing interests, considering the following questions:

- Have you in the past five years received reimbursements, fees, funding, or salary from an organisation that may in any way gain or lose financially from the publication of this manuscript, either now or in the future?
- Do you hold any stocks or shares in an organisation that may in any way gain or lose financially from the publication of this manuscript, either now or in the future?
- Do you hold or are you currently applying for any patents relating to the content of the manuscript?

- Have you received reimbursements, fees, funding, or salary from an organization that holds or has applied for patents relating to the content of the manuscript?
- Do you have any other financial competing interests?
- Do you have any non-financial competing interests in relation to this paper?

If you can answer no to all of the above, write 'I declare that I have no competing interests' below. If your reply is yes to any, please give details below.

I declare that I have no competing interests.

I agree to the open peer review policy of the journal. I understand that my name will be included on my report to the authors and, if the manuscript is accepted for publication, my named report including any attachments I upload will be posted on the website along with the authors' responses. I agree for my report to be made available under an Open Access Creative Commons CC-BY license (http://creativecommons.org/licenses/by/4.0/). I understand that any comments which I do not wish to be included in my named report can be included as confidential comments to the editors, which will not be published.

Choose an item.

To further support our reviewers, we have joined with Publons, where you can gain additional credit to further highlight your hard work (see: https://publons.com/journal/530/gigascience). On publication of this paper, your review will be automatically added to Publons, you can then choose whether or not to claim your Publons credit. I understand this statement.

Yes Choose an item.
