# Supplementary material for: The genome of the venomous snail Lautoconus ventricosus sheds light on the origin of conotoxin diversity
Source: Gigascience. 2021 May 25;10(5):giab037. doi: 10.1093/gigascience/giab037 (PMC8152183; doi:10.1093/gigascience/giab037)
Supplement: giab037_Reviewer_2_Report_Original_Submission — Kevin Kocot -- 3/8/2021 Reviewed [file giab037_reviewer_2_report_original_submission.pdf]

Reviewer Report

Title: The genome of the venomous snail Lautoconus ventricosus sheds light on the origin of conotoxin diversity

Version: Original Submission **Date:** 3/8/2021

Reviewer name: Kevin Kocot

Reviewer Comments to Author:

In this study, RamÃ³n Pardos-Blas et al. report a chromosome-level geome assembly for the Mediterranean cone snail. This is an impressive achievement given the large (3.59 Gb) size of this genome. I think this genome will be a valuable resource for the molluscan genomics community, especially for those interested in gastropods (as only one high-quality caenogastropod genome is available) and venom evolution. I do have a number of mostly minor questions and comments that I think should be addressed.

It is stated that the genome annotations contain 89.2% of the BUSCO metazoan_odb10 genes but it is not stated what the BUSCO score of the scaffolded genome assembly itself is in the main text. This would be helpful in assessing the completeness of the gene models relative to the genome assembly. Likewise please also report all of the BUSCO metrics (e.g., percent fragmented and duplicated) for both the scaffolded genome and gene models in the main text.

The somewhat low BUSCO score for the gene models is attributed to "likely due to the much larger genome size of L. ventricosus" (with respect to other gastropods with higher BUSCO scores). However, the rationale behind this statement is not explained.

On page 3, it is stated that "The main methodological limitation that may explain the missing loci would be the error rate associated to the PacBio CLR sequencing technology (15% [47]), which despite partially corrected by coverage, would hamper BLAST similarity searches." Was any attempt made to polish the genome with the short reads using e.g., Pilon or POLCA?

What is the heterozygosity of the genome? This should be reported in the main text.

Was any attempt made to purge redundant haplotigs from the initial long-read assembly prior to scaffolding?

On page 3 it is stated that "The percentage of complete venom-related loci detected is considerably lower than expected according to general BUSCO results (89.2%). The extra 15% of transcripts of the transcriptome without a gene counterpart in the genome could be isoforms that could be produced naturally during expression or generated as artifacts during transcriptome assembly." Perhaps the Oyster River Protocol or this (admittedly unpublished) approach should be tested?
https://www.biorxiv.org/content/10.1101/2021.02.18.431773v1

Conotoxin transcripts were detected in the foot tissue transcriptome. In the methods, please indicate if these RNA extractions were performed at the same time or not and how the transcriptome libraries were split between the two HiSeq 2000 flowcells. Is it possible there was laboratory contamination or 'bleed through' where some barcodes in the venom gland library were mis-read as barcodes for the foot library, resulting in some reads 'bleeding through' into the foot transcriptome data?

I noticed ferritin is highly expressed in the foot. Any idea why?

I would encourage the authors to make the initial PacBio assembly and as many of the exact commands used and key input/output files for the programs they ran available via e.g., Figshare or Dryad.

The methods state (on page 8) that "After scaffolding, shotgun sequences were used to close gaps between contigs." Not enough detail is provided here to assess what was done.

The methods state (on page 9) "Only models that were predicted by both SNAP and AUGUSTUS were retained in the final annotation set." How was this ensured?

The statement on the availability of supporting data is not clear on whether the predicted transcripts and proteins from the automated genome annotation are made available as fasta files. Please provide these as well.

Minor points:

Correct "preys" to "prey" in the abstract.

Confirm that all instances of "L. ventricosus" are italicized.

Page 2, L column, first paragraph: "The latter…" - Does this refer to snakes?

Page 2, R column, first paragraph: "newly long-read assembled genome" - this is a bit awkward as written.

Page 4, L column, first full paragraph: Add space between "31" and "were"

Page 5, R column, third paragraph: Please clarify what is meant by "an evolutionary successful group of land snails and slugs" here. Pulmonata? All Heterobranchia?

Page 7, L column, second paragraph: The detection of the camlbp I gene is noted but its significance is not explained in detail.

Page 7, L column, third paragraph: There is an English problem here: "sexes of be hermaphrodite"

Page 8, L column, RNA extraction, library preparation, and sequencing section: Correct "grinded" to "ground"

Page 8, R column, last section: I suggest changing "Genome automated annotation" to just "Genome annotation"

Figure 1: The caption does not explain the y-axis for the distributions of protein-coding genes and repetitive elements.

I hope these comments are helpful in improving the manuscript.

Sincerely,

Kevin Kocot

Methods

Are the methods appropriate to the aims of the study, are they well described, and are necessary controls included? Choose an item.

Conclusions

Are the conclusions adequately supported by the data shown? Choose an item.

Reporting Standards

Does the manuscript adhere to the journal's guidelines on minimum standards of reporting? Choose an item.

Choose an item.

Statistics

Are you able to assess all statistics in the manuscript, including the appropriateness of statistical tests used? Choose an item.

Quality of Written English

Please indicate the quality of language in the manuscript: Choose an item.

Declaration of Competing Interests

Please complete a declaration of competing interests, considering the following questions:

- Have you in the past five years received reimbursements, fees, funding, or salary from an organisation that may in any way gain or lose financially from the publication of this manuscript, either now or in the future?
- Do you hold any stocks or shares in an organisation that may in any way gain or lose financially from the publication of this manuscript, either now or in the future?
- Do you hold or are you currently applying for any patents relating to the content of the manuscript?
- Have you received reimbursements, fees, funding, or salary from an organization that holds or has applied for patents relating to the content of the manuscript?
- Do you have any other financial competing interests?
- Do you have any non-financial competing interests in relation to this paper?

If you can answer no to all of the above, write 'I declare that I have no competing interests' below. If your reply is yes to any, please give details below.

I declare that I have no competing interests.

I agree to the open peer review policy of the journal. I understand that my name will be included on my report to the authors and, if the manuscript is accepted for publication, my named report including any attachments I upload will be posted on the website along with the authors' responses. I agree for my report to be made available under an Open Access Creative Commons CC-BY license (http://creativecommons.org/licenses/by/4.0/). I understand that any comments which I do not wish to be included in my named report can be included as confidential comments to the editors, which will not be published.

Choose an item.

To further support our reviewers, we have joined with Publons, where you can gain additional credit to further highlight your hard work (see: https://publons.com/journal/530/gigascience). On publication of this paper, your review will be automatically added to Publons, you can then choose whether or not to claim your Publons credit. I understand this statement.

Yes Choose an item.
